# Altered choline level in atherosclerotic lesions: Upregulation of choline transporter-like protein 1 in human coronary unstable plaque

**DOI:** 10.1371/journal.pone.0281730

**Published:** 2023-02-17

**Authors:** Eriko Nakamura, Kazunari Maekawa, Yoichi Saito, Tomoko Matsumoto, Mikako Ogawa, Yoshihiro Komohara, Yujiro Asada, Atsushi Yamashita

**Affiliations:** 1 Department of Pathology, Faculty of Medicine, University of Miyazaki, Miyazaki, Japan; 2 Bioengineering Lab, Faculty of Advanced Science and Technology, Kumamoto University, Kumamoto, Japan; 3 Center for Collaborative Research and Community Cooperation, University of Miyazaki, Miyazaki, Japan; 4 Laboratory of Bioanalysis and Molecular Imaging, Graduate School of Pharmaceutical Science, Hokkaido University, Sapporo, Hokkaido, Japan; 5 Department of Cell Pathology, Graduate School of Medical Sciences, Faculty of Life Sciences, Kumamoto University, Kumamoto, Japan; 6 Department of Pathology, Miyazaki Medical Association Hospital, Miyazaki, Japan; University of Canterbury, NEW ZEALAND

## Abstract

Inflammatory activity and hypoxia in atherosclerotic plaques are associated with plaque instability and thrombotic complications. Recent studies show that vascular cell metabolism affects atherogenesis and thrombogenicity. This study aimed to identify the metabolites in macrophage-rich unstable plaques that modulate atherogenesis and serve as potential markers of plaque instability. Atherosclerotic plaques were induced by balloon injury in the iliofemoral arteries of rabbits fed on a conventional or 0.5% cholesterol diet. At 3 months post-balloon injury, the arteries and cardiac tissues were subjected to histological, quantitative real-time polymerase chain reaction, and metabolomic analyses. The identified metabolite-related proteins were immunohistochemically analyzed in stable and unstable plaques from human coronary arteries. The factors modulating the identified metabolites were examined in macrophages derived from human peripheral blood mononuclear cells. Metabolomic analysis revealed that choline and guanine levels in macrophage-rich arteries were upregulated compared with those in non-injured arteries and cardiac tissues. Vascular choline levels, but not guanine levels, were positively correlated with the areas immunopositive for macrophages and *tumor necrosis factor (TNF)-α* and *matrix metalloproteinase (MMP) 9* mRNA levels in injured arteries. In human coronary arteries, choline transporter-like protein (CTL) 1 was mainly localized to macrophages within plaques. The area that was immunopositive for CTL1 in unstable plaques was significantly higher than that in stable plaques. Intracellular choline levels were upregulated upon stimulation with TNF-α but were downregulated under hypoxia in cultured macrophages. Administration of choline upregulated the expression of *TNF-α* and *CTL1* mRNA in cultured macrophages. The transfection of *CTL1* small interfering RNA decreased *CTL1*, *TNF-α*, and *MMP9* mRNA levels in cultured macrophages. These results suggest that choline metabolism is altered in macrophage-rich atherosclerotic lesions and unstable plaques. Thus, CTL1 may be potential markers of plaque instability.

## Introduction

Atherosclerotic complications are the major etiological factors for the onset of cardiovascular disease (CVD), which is the leading cause of mortality worldwide [1]. Coronary atherosclerosis is associated with a prolonged asymptomatic period but rapidly becomes symptomatic with plaque disruption and thrombus formation, resulting in acute myocardial infarction. Therefore, early diagnosis and evaluation of coronary atherosclerosis at a stage when the disease is responsive to therapy is important.

Inflammatory activity in coronary atherosclerotic plaques is among the most important factors associated with plaque instability and future CVD onset [2]. Serum C-reactive protein levels are a predictive marker for CVDs. Downregulated C-reactive protein decreases the probability of cardiac events in individuals without hyperlipidemia [3]. Although clinical evidence is strong for serum markers of atherosclerotic diseases, marker levels may not always reflect the inflammatory milieu in atherosclerotic plaques.

Recent metabolomic studies have demonstrated that vascular cell metabolism affects and reflects atherogenesis [4, 5]. Previously, we reported that glycolysis is altered in atherosclerotic lesions under insulin-dependent diabetic conditions [6]. Meanwhile, the kynurenine pathway promotes the thrombogenic potential of activated macrophages in atherosclerotic lesions [7]. These studies have elucidated a correlation between vascular cell metabolism and atherosclerosis. However, few studies have analyzed specific metabolites in atherosclerotic lesions.

This study aimed to identify the metabolites in macrophage-rich unstable plaques that modulate atherogenesis and are potential markers of plaque instability. We identified choline as an abundant metabolite in macrophage-rich atherosclerotic arteries using a rabbit atherosclerosis model. Additionally, we analyzed the localization of choline transporter-like protein 1 (CTL1, a choline transporter) and choline kinase α (ChoKα, a choline anabolic enzyme) in human coronary plaques and their correlation with plaque instability. Furthermore, the factors modulating cellular choline levels and the effects of choline and CTL1 expression on cultured macrophages/foam cells and smooth muscle cells (SMCs) were identified.

## Methods

### Rabbit model of atherosclerosis

This study was approved by the Animal Care Committee of the University of Miyazaki (No. 2010–511). Male Japanese white rabbits (n = 10) with a body weight of 2.5–3.0 kg were fed a conventional or 0.5% cholesterol diet. A balloon catheter was used to induce atherosclerotic lesions in the right iliofemoral artery. At 3 monthss post-balloon injury, samples were subjected to metabolomic, histological, and quantitative real-time polymerase chain reaction (qRT-PCR) analyses.

All surgical procedures were performed under sterile conditions. General anesthesia was administered by subcutaneous injection of an anesthetic mixture comprising medetomidine (0.16 mg/kg body weight, ZENOAQ, Nippon Zenyaku Kogyo Co., Ltd., Fukushima, Japan), midazolam (0.8 mg/kg body weight, Fuji Pharma Co., Ltd., Tokyo, Japan), and butorphanol (0.04 mg/kg body weight, Meiji Seika Pharma Co., Ltd., Tokyo, Japan). An angioplasty balloon catheter (diameter, 2.5 mm; length, 15 mm; QUANTUM, Boston Scientific, Marlborough, MA, USA) was inserted into the right femoral artery 1 week post-feeding with a conventional or 0.5% cholesterol diet (n = 5 in each group) to induce atherosclerotic lesions. The catheter was inflated to 1.5 atm and retracted three times to denude the endothelium [8]. At 3 months post-balloon injury induction, the rabbits were fasted for 6 h and were injected with heparin (500 U/kg body weight; intravenous (i.v.)). The rabbits were euthanized with an overdose of pentobarbital (60 mg/kg body weight, i.v.). Next, the rabbits were perfused with 50 mL of saline and the bilateral iliofemoral arteries and cardiac tissues were excised. The iliofemoral artery and left ventricle of the heart were cut into three pieces. One piece was placed in a cryotube, frozen in liquid nitrogen, and stored at −80°C until metabolomic analysis. Meanwhile, one piece was fixed in 4% paraformaldehyde for histological analysis and the final piece was immersed in RNAlater solution (Qiagen, Hilden, Germany) for qRT-PCR analysis.

### Metabolomic analysis of rabbit iliofemoral arteries and cardiac tissue

Metabolomic analysis was performed using non-injured and injured arteries and cardiac tissues (n = 5 in each group) obtained from rabbits fed on a conventional or 0.5% cholesterol diet (n = 5 in each group). Metabolites were extracted from arteries and cardiac tissues. Arterial extraction and metabolomic analysis were performed by Human Metabolome Technologies, Inc. (HMT) (Tsuruoka, Yamagata, Japan). Briefly, the metabolomic profiles were investigated using capillary electrophoresis time-of-flight mass spectrometry (CE-TOFMS) (Agilent Technologies, Santa Clara, CA, USA). Measurements in positive and negative ion modes were performed under previously reported conditions [9]. The spectrometer scanned the samples from 50 to 1,000 m/z. The raw data obtained using CE-TOFMS were automatically processed using MasterHands automatic integration software (Keio University Tsuruoka, Tsuruoka, Yamagata, Japan) to obtain peak information, including m/z, peak area, and migration time. Principal component analysis and hierarchical clustering analysis with heatmapping were performed using proprietary HMT statistical analysis software.

### Histological and immunohistochemical analyses of rabbit iliofemoral arteries and cardiac tissues

Excised iliofemoral arteries and left cardiac ventricles were fixed in 4% paraformaldehyde for 12 h at 4°C and embedded in paraffin. Sections (3 μm thickness) were stained with hematoxylin-eosin/Victoria blue (HE/VB) and subjected to immunohistochemistry using antibodies against rabbit macrophage antigen (mouse monoclonal, RAM11, DAKO, Santa Clara, CA, USA), α-smooth muscle actin (α-SMA), a marker of SMC (mouse monoclonal, 1A4, DAKO), and α cardiac actin, a cardiomyocyte marker (mouse monoclonal, ACTC1, Proteintech, Rosemont, IL, USA).

The immunopositive area was analyzed using a WinROOF color imaging morphometry system (Mitani, Fukui, Japan) [10]. Data are expressed as the ratio of the immunopositive area to the vascular area (%).

### RNA preparation and qRT-PCR analysis for the rabbit model

After the iliofemoral arteries were excised, neointimal tissue was peeled from the media and adventitia and divided into 5-mm segments. Total RNA was isolated using TRIzol reagent (Invitrogen, Carlsbad, CA, USA) and PureLink RNA mini kits (Life Technologies). RNA was reverse transcribed into complementary DNA (cDNA) using Primescript RT Master Mix Kits (Takara Bio, Kusatsu, Japan). The amount of RNA was quantified using a spectrophotometer (ND-1000, Thermo Scientific, Rockford, IL, USA). The mRNA expression levels of *interleukin (IL)-6*, *matrix metalloproteinase (MMP) 9*, *tissue factor (TF)*, *tissue necrosis factor (TNF)-α*, and *hydroxymethylbilane (HMBS)* were determined using TB Green Premix Taq (Takara Bio) as described in S1 Table. The mRNA levels of the target genes were normalized to those of *HMBS*, and qRT-PCR analysis was performed using a LightCycler 96 system (Roche Diagnostics GmbH, Mannheim, Germany).

### Histological and immunohistochemical analyses of human coronary arteries

The ethics committee of the University of Miyazaki approved the study protocol (Approval No. 2015–186 O). Because this study is a retrospective study on autopsy cases, we applied opt-out method to obtain consent. All data were fully anonymized.

Human coronary arteries were obtained from 17 autopsy cases (six cases of non-cardiac death and 11 cases of myocardial infarction) at the University of Miyazaki Hospital. Samples were fixed in formalin and sectioned. Cross sections (3 μm thickness) of the coronary arteries were obtained from the proximal portion of the right and left coronary arteries in each case. Coronary lesions were histologically classified into early (diffuse intimal thickening), stable (pathological intimal thickening, fibrous cap atheroma, or fibrocalcific plaque), or unstable [thin (< 65 μm) fibrous cap atheroma, intraplaque hemorrhage, or plaque rupture] lesions, as defined by Virmani et al. [11]. The sections were immunohistochemically analyzed using antibodies against α-SMA (mouse monoclonal, 1A4, DAKO), CD68 (mouse monoclonal, PG-M1, DAKO), CTL1 (rabbit polyclonal, SLC44A1, Atlas Antibodies, Stockholm, Sweden), and ChoKα (rabbit polyclonal, Proteintech). Immunoreactive signals were visualized using an EnVision system (DAKO). Microscopic digital images of sections subjected to α-SMC and macrophage immunohistochemistry were captured with a digital camera (DP-74, OLYMPUS, Tokyo, Japan) under a 1.25× objective lens. Microscopic digital images of samples subjected to CTL1 and ChoKα immunohistochemistry were captured in the most densely stained area in each arterial section using a 40× objective lens. This imaging was performed because the CTL1 or ChoKα immunoreactive areas were localized to areas with macrophage accumulation or were difficult to measure in low-magnification images. Immunopositive areas in the coronary arteries were assessed using a WinROOF color imaging morphometry system [10].

### Isolation of human peripheral blood mononuclear cells (PBMCs) and cell culture experiments

The ethics committee of the University of Miyazaki approved the study protocol (Approval No. O-0224). We obtained written informed consents from healthy volunteers. Human peripheral blood mononuclear cells (PBMCs) were isolated from the venous blood of the healthy volunteers using density-gradient centrifugation with Ficoll-Paque PLUS (GE Healthcare, Chicago, IL, USA). Human PBMCs were seeded into 100 × 20 mm Primaria cell culture dishes (CORNING, Inc., Durham, NC, USA) and maintained in a subconfluent state (5 × 10^5^ cells/mL) in Roswell Park Memorial Institute (RPMI)-1640 medium. The mixture was supplemented with macrophage-colony stimulating factor (50 ng/mL; PEPROTECH, Rocky Hill, NJ, USA), 10% human heat-inactivated serum, and 1% Zell Shield (Minerva Biolabs, Berlin, Germany) incubated at 37°C in a humidified incubator containing 5% CO_2_ for 7 days to allow differentiation into macrophages [12]. Afterward, non-adherent cells and media were removed. Adherent cells, which were regarded as macrophages, were detached from the dishes using Accutase reagent (Innovate Cell Technologies, Inc., San Diego, CA, USA). The macrophages were uniformly reseeded into 6-well plates (3 × 10^5^ cells/mL) and cultured in RPMI-1640 (20 μM choline, 2 mL) supplemented with 10 μM choline, 10% human heat-inactivated serum, and 1% Zell Shield.

The cells were stimulated with TNF-α (10 ng/mL, Sigma-Aldrich), interferon (IFN)γ (50 ng/mL, R&D Systems, Minneapolis, MN, USA), and IL-4 (20 ng/mL, Sigma-Aldrich) for 3 days, cultured under hypoxia (1% O_2_) for 1 d or treated with oxidized low-density lipoprotein (oxLDL, 50 μg/mL) for 2 days to measure cellular choline levels.

To examine the effect of choline concentration on expression of *CTL1*, *TNF-α*, *IL-6*, and *MMP9* mRNA, differentiated macrophages were uniformly reseeded into 6-well plates (3 × 10^5^ cells/ mL) and cultured in RPMI-1640 (20 μM choline) supplemented with 0 μM (control), 10 μM, or 80 μM choline for up to 3 days at 37°C in a humidified incubator containing 5% CO_2_. Primers for human *CTL1*, *TNF-α*, *IL-6*, and *MMP9* genes are described in S2 Table.

### Preparation of oxLDL and foam cell induction

The concentration of purified human LDL (density; 5.7 mg/mL, 2 mg, Millipore) was adjusted to 0.1 mg/mL protein using phosphate-buffered saline (PBS, pH 7.4). To perform Cu^2+^-mediated oxidation, the samples were dialyzed against 5 μM CuSO_4_/PBS at 37°C for 24 h. Oxidation was terminated by adding ethylenediaminetetraacetic acid (EDTA) to a final concentration of 1 mM. The samples were dialyzed against PBS for 24 h at 4°C (Biotech Grade Dialysis Membranes, Spectra/Por). Oxidized low-density lipoprotein was concentrated, filtered through an Amicon Ultra-15 Ultracel-100 kDa centrifugal filter, sterilized, and filtered through a Millex-HV membrane [13]. Concentration of LDL was measured using Pierce bicinchoninic acid (BCA) protein assay kits (Thermo Scientific, Rockford, IL, USA).

Human peripheral blood mononuclear cell-derived macrophages were plated in 6-well tissue culture plates at a density of 3 × 10^5^ cells/mL. Subconfluent cells were incubated with oxLDL (50 μg/mL) in serum-free medium for 2 days. Foam cell formation was confirmed using Oil Red O staining.

### Liquid chromatography-tandem mass spectrometry (LC-MS/MS) analysis of intracellular choline concentrations

Non-adherent PBMCs were removed by washing with PBS. Stimulated and differentiated macrophages were retrieved and detached using a scraper and methanol (1000 μL). The samples were vortexed using a Bioruptor U-250 (Cosmo Bio, Tokyo, Japan) and centrifuged at 2300 *g* at 4°C for 5 min. The supernatant was stored at −80°C. The pellet comprising cellular proteins was lysed using radioimmunoprecipitation assay buffer (RIPA, Nacalai Tesque, Kyoto, Japan) containing 1% Halt protease and phosphatase inhibitors (Life Technologies). Protein concentrations were determined using Pierce BCA Protein Assay kits (Thermo Scientific). Cellular choline levels were measured using liquid chromatography-tandem mass spectrometry. The LC conditions were as follows: column, Ascentis Express 150 mm × 2.1 mm HILIC column with a particle size of 2.7 μm (Sigma, St Louis, MO, USA); temperature, 25°C; mobile phase, 2% solution A (ammonium formate; pH 3) and 98% solution B (acetonitrile). Calibration curves were generated using choline chloride (Sigma-Aldrich) [14]. Samples (10 μL) were injected into the column and detected using a Q Exactive mass spectrometer (Life Technologies, Carlsbad, CA, USA).

### CTL1 inhibition in macrophages using small interfering RNA (siRNA)

Differentiated macrophages were uniformly seeded into 12-well plates (3 × 10^5^ cells/ mL) and cultured in RPMI-1640. The cells were treated with 80 μM choline for 3 days at 37°C in a humidified incubator containing 5% CO_2._ Macrophages were transfected by incubating siRNA targeting CTL1 (Horizon, Cambridge, UK) or negative control siRNA (Horizon) at a final concentration of 1 μM. siRNAs were mixed with Accell siRNA delivery media (Horizon) for 3 days at 37°C in a humidified incubator containing 5% CO_2_, according to the manufacturer’s instructions. Then, *CTL1*, *TNF-α*, *IL-6*, and *MMP9* mRNA levels were assayed 3 days after the transfection using qPCR as described below.

### Vascular smooth muscle cell culture

Primary human coronary artery SMCs (HCASMCs) (Lonza, Basel, Switzerland) were cultured in SMC basal medium (SmBM; the cocktail medium was used throughout this study, Lonza) supplemented with an SMC growth medium-2 BulletKit (Lonza). After trypsinization, cells were detached, counted, and equally reseeded in 6-well plates (1 × 10^5^ cells/mL). Cells were then cultured in reduced serum medium (final fetal bovine serum [FBS] concentration, 0.1%) for 16 h. After incubation for 16 h, the medium was replaced. The cells were treated with 0 μM (control), 50 μM, or 100 μM choline and cultured for up to 3 days at 37°C in a humidified incubator containing 5% CO_2_. mRNA levels of *CTL1*, *TNF-α*, *IL-6*, and *MMP9* were assayed using qPCR as described below.

### RNA isolation and qRT-PCR analysis of cultured cells

Cells were cultured as described above. The cell culture supernatant was removed and the cells were washed twice with 2 mL ice cold PBS. TRIzol reagent (Invitrogen, Carlsbad, CA, USA) was added to the cells and total RNA was extracted using PureLink RNA Mini Kits. The amount of RNA was quantified using a NanoDrop 1000 (ND-1000; Thermo Scientific, Rockford, IL) spectrophotometer. cDNA was synthesized using PrimeScript RT reagent kits (Perfect Real Time; Takara Bio, Shiga, Japan). The mRNA expression of *CTL1*, *TNF-α*, *IL-6*, *MMP9*, *and β-actin* were determined using TB Green Premix Taq (Takara Bio). Primer sequences are shown in S2 Table. Real-time PCR was performed using a LightCycler 96 instrument (Roche Diagnostics GmbH, Mannheim, Germany). The mRNA of the target genes were normalized to those of *β-actin*.

### Statistical analysis

All statistical analyses were performed using GraphPad Prism 7 (GraphPad Software, Inc., San Diego, CA, USA). Data are expressed as the mean ± standard deviation or as individual data points. Data were compared using Welch’s t-tests, Mann-Whitney U tests, Kruskal-Wallis tests, followed by Dunn’s multiple comparisons test, or two-way ANOVA followed by Tukey’s multiple comparison tests. Correlations between factors were evaluated using Spearman’s tests. Differences were considered statistically significant at p < 0.05.

## Results

### Differential effects of conventional and 0.5% cholesterol diets on neointimal formation in injured rabbit iliofemoral arteries

**Fig 1:**
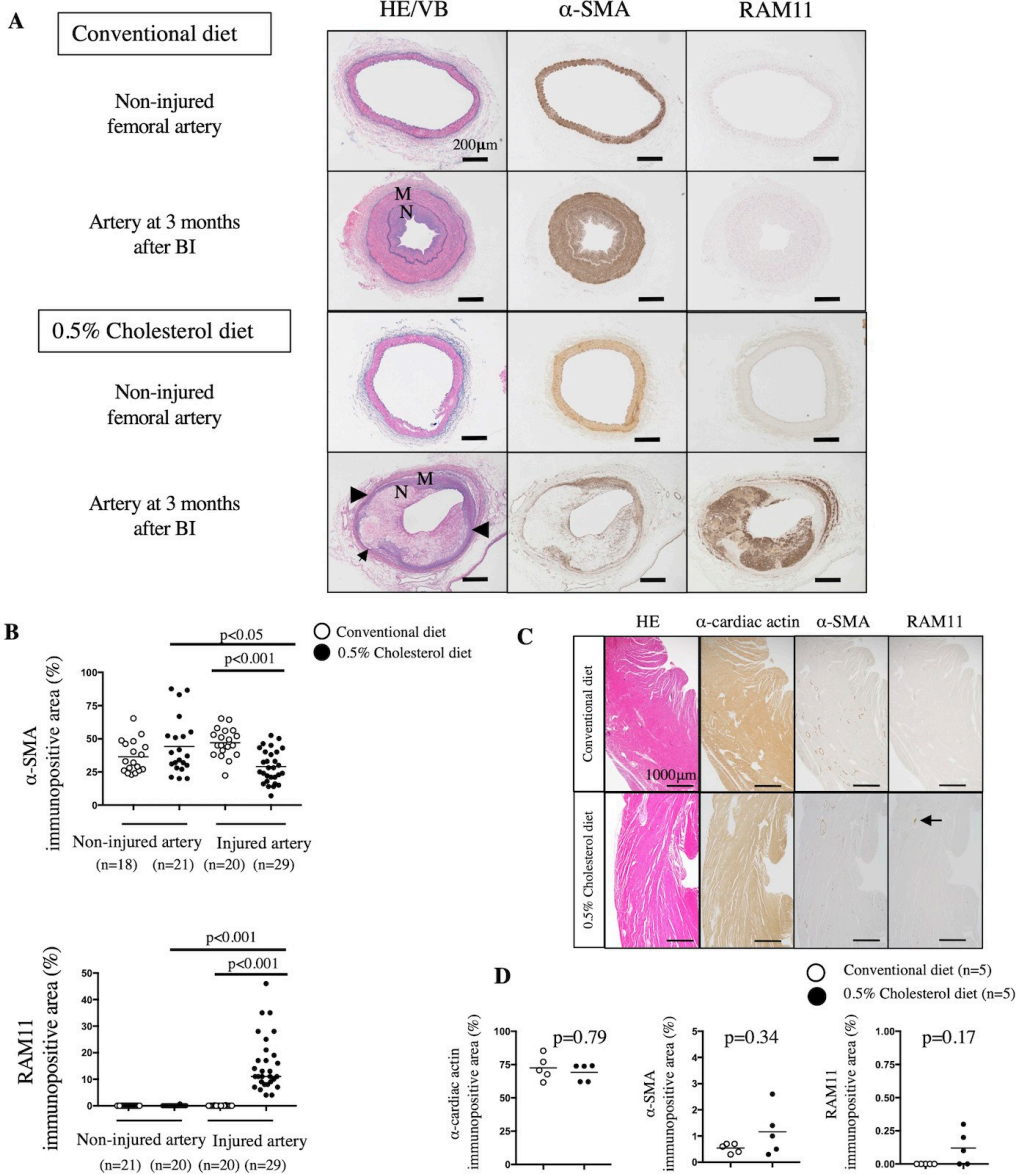
Representative histological images of rabbit iliofemoral arteries, cardiac tissues, and their cellular components. (A) Representative histological images of rabbit iliofemoral arteries stained with hematoxylin-eosin/Victoria blue (HE/VB), antibodies for α-smooth muscle actin (SMA), or rabbit macrophage-specific antibodies (RAM11). The injured arteries exhibited the formation of concentric neointima rich in smooth muscle cells (SMCs) in rabbits fed a conventional diet. In rabbits fed a 0.5% cholesterol diet, injured arteries exhibited the formation of a large neointima comprising macrophages, SMCs, and extracellular matrix. Medial thinning (arrowheads) and disruption of the elastic lamina (arrow) were observed. Neointimal formation was not observed in the non-injured arteries of rabbits fed a conventional or 0.5% cholesterol diet. N, neointima; M, media; BI, balloon injury. Scale bars represent 200 μm. (B) Immunopositive areas of α-SMA (an SMC marker) and RAM11 (a rabbit macrophage marker) in the non-injured or injured arteries of rabbits fed a conventional or 0.5% cholesterol diet. α-SMA immunopositive area in non-injured arteries from the conventional diet group (36.4 ± 11.9%) or the 0.5% cholesterol diet group (44.2 ± 20.8%), α-SMA immunopositive area in injured arteries from the conventional diet group (47.0 ± 10.3%) or the 0.5% cholesterol diet group (29.1 ± 12.0%). RAM11 immunopositive area in a non-injured artery from the conventional diet group (0 ± 0%) or the 0.5% cholesterol diet group (0.03 ± 0.15%), RAM11 immunopositive area in the injured artery of conventional diet (0.02 ± 0.05%) or 0.5% cholesterol diet (15.7 ± 10.1%). Data are represented as mean ± standard deviation, and individual data points and median for each group. Data were analyzed by Kruskal-Wallis tests followed by Dunn’s multiple comparison tests. (C) Representative histological images of rabbit cardiac tissues stained with HE, α-cardiac actin (a cardiomyocyte marker), α-SMA, or RAM11 antibodies. Cardiomyocytes are the major cellular components of cardiac tissues. Vascular SMCs were observed in the myometrium. Small foci of macrophage infiltration (arrow) were observed in the small arterial walls of rabbits fed a 0.5% cholesterol diet. Scale bars represent 1000 μm. (D) Immunopositive areas of α-cardiac actin, α-SMA, and RAM11 in the cardiac tissues of rabbits fed a conventional or a 0.5% cholesterol diet. α-cardiac immunopositive area in cardiac tissue from the conventional diet group (72 ± 8.1%) or the 0.5% cholesterol diet group (69.2 ± 5.8%). α-SMA immunopositive area in cardiac tissue from the conventional diet group (0.5 ± 0.2%) or the 0.5% cholesterol diet group (1.2 ± 0.8%). RAM11 immunopositive area in cardiac tissue from the conventional diet group (0 ± 0%) or the 0.5% cholesterol diet group (0.1 ± 0.1%). Data are represented as mean ± standard deviation, and individual data points and median for each group. Data were analyzed using Mann-Whitney U tests.

Balloon injury induced the formation of a neointima layer comprised of SMCs and extracellular matrix in rabbits fed a conventional diet but induced the formation of a large neointima layer comprised of abundant macrophages, SMCs, and extracellular matrix in rabbits fed a 0.5% cholesterol diet (Fig 1A). In contrast, neointimal formation was not observed in the non-injured arteries of rabbits fed either the conventional or 0.5% cholesterol diet. The α-SMA-immunopositive area in the injured arteries of rabbits fed a 0.5% cholesterol diet was significantly smaller than that in the non-injured arteries of rabbits fed a conventional or 0.5% cholesterol diet or the injured arteries of rabbits fed a conventional diet (Fig 1B). Meanwhile, the RAM11-immunopositive area in the injured arteries of rabbits fed a 0.5% cholesterol diet was significantly larger than that in the non-injured arteries of rabbits fed a 0.5% cholesterol diet or the injured arteries of rabbits fed a conventional diet (Fig 1B). Thus, injured iliofemoral arteries in rabbits fed a conventional diet were defined as arteries with SMC-rich neointima, whereas those in rabbits fed a 0.5% cholesterol diet were defined as arteries with macrophage-rich neointima. α-cardiac actin-immunopositive cardiomyocytes are the major cellular components of the left cardiac ventricle in rabbits. The immunopositive areas of α-cardiac actin, α-SMA, and RAM11 were not significantly different between rabbits fed a conventional diet and those fed a 0.5% cholesterol diet (Fig 1C and 1D).

### mRNA levels of *TNF-*α, *MMP9*, *TF*, and *IL-6* in arteries with SMC- and macrophage-rich neointima

To characterize arteries with SMC- and macrophage-rich neointima, we evaluated the mRNA levels of pro-inflammatory cytokines (*IL-6* and *TNF****-***α), prothrombotic factor (*TF*), and plaque instability marker (*MMP9*) using qRT-PCR. Adventitia was removed before mRNA extraction because this layer abundantly expresses *TF* [15].

**Fig 2:**
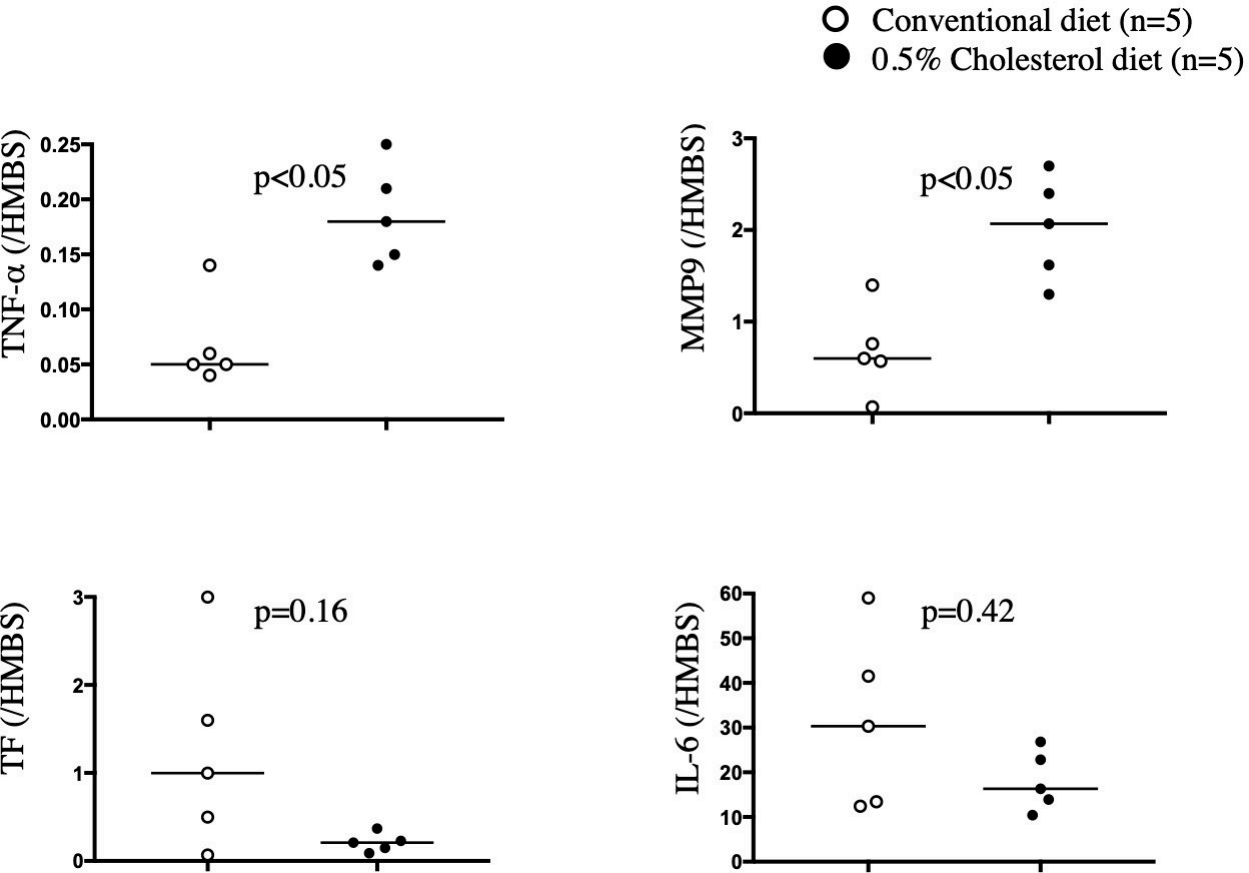
*TNF-*α, *MMP9*, *TF*, and *IL-6* mRNA levels in the neointima of injured arteries. Neointimal RNA from rabbit iliofemoral arteries was isolated and reverse transcribed into cDNA. qRT-PCR was performed for *interleukin (IL)-6*, *matrix metalloproteinase (MMP) 9*, *tissue factor (TF)*, *tissue necrosis factor (TNF)-α*, and *hydroxymethylbilane (HMBS)*. Target gene expression levels were normalized to those of *HMBS*. Neointimal *TNF-α* mRNA level in the conventional diet group (0.07 ± 0.04) or the 0.5% cholesterol diet group (0.2 ± 0.04). Neointimal *TF* mRNA levels from the conventional diet group (1.2 ± 1.0) or the 0.5% cholesterol diet group (0.2 ± 0.09). Neointimal *MMP9* mRNA levels from the conventional diet group (0.7 ± 0.4) or the 0.5% cholesterol diet group (2 ± 0.5). Neointimal *IL-6* mRNA level in the conventional diet group (31.3 ± 17.6) or the 0.5% cholesterol diet group (18 ± 6). Data are represented as mean ± standard deviation, and individual data points and median for each group. Data were analyzed using Mann-Whitney U tests.

The mRNA levels of *TNF****-***α and *MMP9* in arteries with macrophage-rich neointima were significantly higher than those in arteries with SMC-rich neointima. *Tissue factor* and *IL-6* mRNA levels were not significantly different between arteries with SMC-rich neointima and those with macrophage-rich neointima (Fig 2).

### Metabolomic analysis of injured and non-injured arteries and cardiac tissues of rabbits fed a conventional diet or a 0.5% cholesterol diet

To comprehensively evaluate the metabolic status, non-injured and injured arteries and cardiac tissues from rabbits fed a conventional or a 0.5% cholesterol diet were subjected to metabolomic analysis using CE-TOFMS at 3 months post-balloon injury. Metabolomic analysis identified 260 cationic and anionic metabolites.

**Fig 3:**
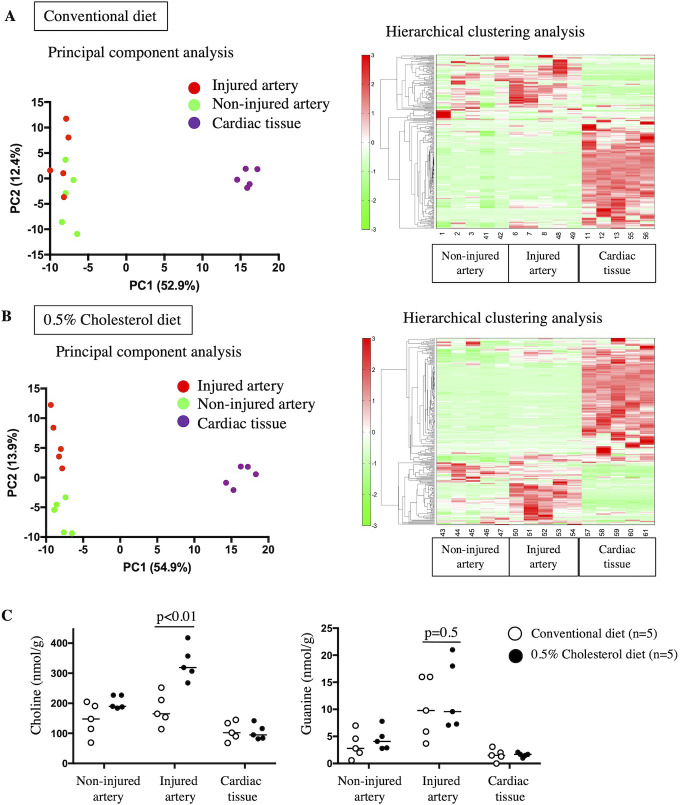
Metabolic analysis of arteries and cardiac tissues of rabbits fed a conventional or 0.5% cholesterol diet. (A) Principal component (PC) and hierarchical clustering analyses showed that the metabolic features of arteries were distinct from those of cardiac tissues in rabbits fed a conventional diet. The metabolic abundance was not significantly different between the non-injured and injured arteries. The data used for PC and hierarchical clustering analyses are shown in S3 and S4 Tables, respectively. The red and green densities indicate high and low metabolite concentrations, respectively. (B) PC and hierarchical clustering analyses distinguished the metabolic features of arteries from those of cardiac tissues in rabbits fed a 0.5% cholesterol diet. The dashed box indicates the abundant metabolites in the injured arteries relative to those in non-injured arteries and cardiac tissues. The data used for PC or hierarchical clustering analysis are shown in S5 and S6 Tables, respectively. The red and green densities indicate high and low metabolite concentrations, respectively. (C) Arterial and cardiac choline and guanine levels in rabbits fed a conventional or 0.5% cholesterol diet. Choline levels in non-injured arteries from the conventional diet group (145.6 ± 49.8 nmol/g) or the 0.5% cholesterol diet group (203.2 ± 19.5 nmol/g). Choline levels in injured arteries from the conventional diet group (179.2 ± 47.7 nmol/g) or the 0.5% cholesterol diet group (333.8 ± 50.8 nmol/g). Choline levels in cardiac tissue from the conventional diet group (107.8 ± 28.3 nmol/g) or the 0.5% cholesterol diet group (103.8 ± 22.6 nmol/g). Guanine levels in non-injured arteries from the conventional diet group (3.4 ± 2.2 nmol/g) or the 0.5% cholesterol diet group (4.5 ± 1.8 nmol/g). Guanine levels in injured arteries in the conventional diet group (10.3 ± 5.1 nmol/g) or the 0.5% cholesterol diet group (12.6 ± 5.8 nmol/g). Guanine levels in cardiac tissue from the conventional diet group (1.6 ± 1.0 nmol/g) or the 0.5% cholesterol diet group (1.6 ± 0.4 nmol/g). The data are represented as mean ± standard deviation, and individual data points and median for each group. Data were analyzed using Mann-Whitney U tests.

Fig 3A and S3 and S4 Tables show the principal component analysis and hierarchical clustering analysis of the metabolome of the arteries and cardiac tissues of rabbits fed a conventional diet. The hierarchical tree indicates the relative distance between the metabolite peaks. The metabolite levels in the arteries were significantly different from those in the cardiac tissues. Principal component and hierarchical analyses revealed that metabolites in arteries and cardiac tissues were separated along principal component 1, whereas those in injured and non-injured arteries were not markedly separated.

Fig 3B and S5 and S6 Tables show the principal component and hierarchical clustering analyses of arteries and cardiac tissues of rabbits fed a 0.5% cholesterol diet. Principal component analysis revealed distinct metabolic features of injured arteries, non-injured arteries, and cardiac tissues. Several metabolites, especially central carbon metabolites (lactic acid, glycerol 3-phosphate, glucose 6-phosphate, and fructose 1,6-diphosphate), amino acids (Asn, Gln, Glu, and Ala), and purines (adenosine monophosphate, inosine monophosphate, and adenosine diphosphate), in the cardiac tissues were higher than those in arteries of rabbits fed a 0.5% cholesterol diet (S7 Table). Hierarchical clustering analysis revealed a metabolite cluster that was abundant in injured arteries relative to non-injured arteries and cardiac tissues. The relative levels of the following seven metabolites (detectable in more than four of the five injured and non-injured arteries) in the injured arteries were higher than those in the non-injured arteries and cardiac tissues: quinolinic acid, O-succinyl-l-homoserine, betaine aldehyde + H_2_O, 5-hydroxylysine, thiaproline, guanine, and choline (S8 Table).

Among these metabolites, choline and guanine were selected as quantifiable metabolites in all samples (S7 and S9 Tables). Additionally, injured arterial choline levels in rabbits fed a 0.5% cholesterol diet were significantly higher than those in rabbits fed a conventional diet (Fig 3C). However, the guanine levels in injured arteries in rabbits fed a conventional diet were not significantly different from those in rabbits fed a 0.5% cholesterol diet.

### Correlation of arterial choline and guanine levels with cell type and gene expression in injured rabbit arteries

**Fig 4:**
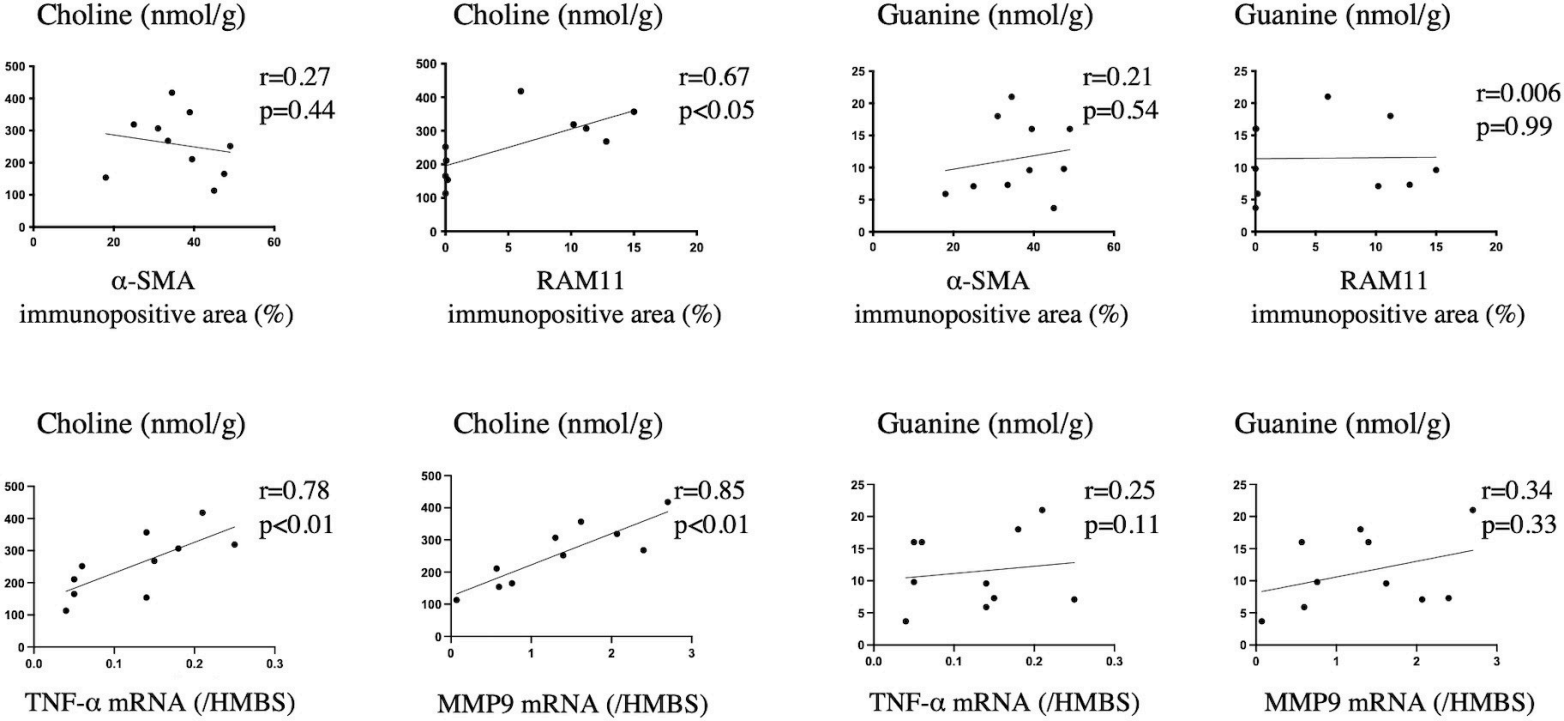
Correlation of arterial choline and guanine levels with vascular components. Correlation of arterial choline and guanine levels with α-SMA- and RAM11- immunopositive areas and *TNF-α* and *MMP9* mRNA levels (n = 10 each; Spearman’s test).

Fig 4 shows the correlation between arterial choline and guanine levels with SMC-immunopositive and macrophage-immunopositive areas and *TNF-α* and *MMP9* mRNA levels in injured arteries. Arterial choline levels were positively correlated with the macrophage-immunopositive area (r = 0.67, p < 0.05, n = 10) and *TNF-α* (r = 0.78, p < 0.01, n = 10) and *MMP9* mRNA levels (r = 0.85, p < 0.01, n = 10). However, the arterial guanine levels did not correlate with these factors.

### Expression of CTL1 and ChoKα in human coronary arteries

To determine CTL1 (a choline transporter) and ChoKα (a choline anabolic enzyme) expression in human coronary arteries, the proximal portions of the right and left coronary arteries from autopsy cases were subjected to histological analysis. The clinical characteristics of the autopsy cases are shown in S10 Table. Among the 134 coronary artery sections, 22, 87, and 25 cases were classified as early, stable, and unstable, respectively.

**Fig 5:**
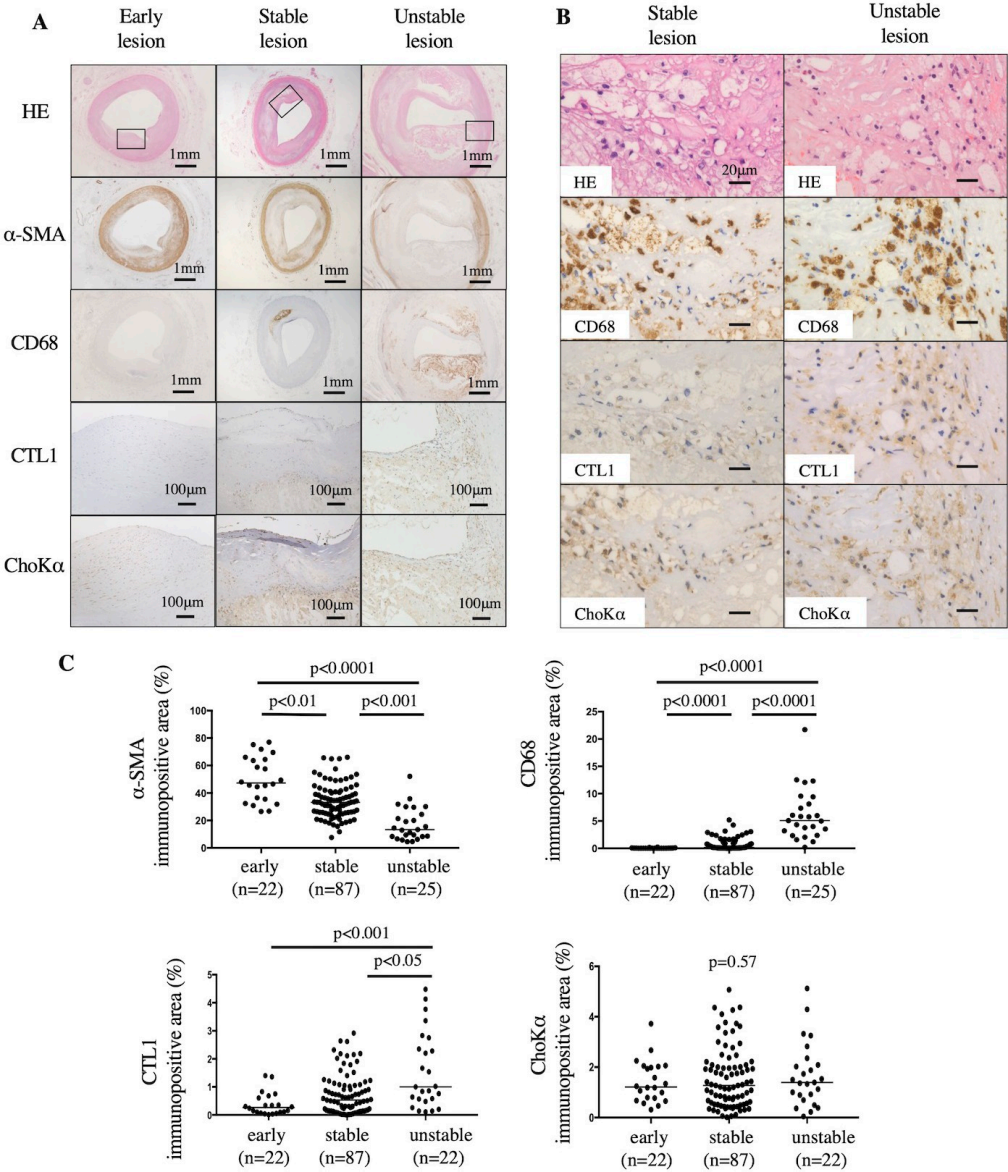
CTL1 and ChoKα expression levels in the human coronary artery. (A) Representative histological images of human coronary arteries stained with hematoxylin and eosin (HE) and antibodies for α-smooth muscle actin (SMA), macrophages (CD68), choline transporter-like protein 1 (CTL1), and choline kinase α (ChoKα). Early lesions exhibited diffuse immunoreactivity for α-SMA and ChoKα in areas with intimal thickening. Stable lesions exhibited immunoreactivity for CD68 and ChoKα in the lipid core of the fibrous cap atheroma and focal immunoreactivity for α-SMA in the plaque. Unstable lesions exhibited immunoreactivity for CD68, CTL1, and ChoKα in thin-cap fibroatheromas and sparse α-SMA immunoreactivity in plaques. Squares in each image (HE-stained section) correspond to immunohistochemical images of CTL1 and ChoKα. Scale bars represent 1 mm or 100 μm. (B) High-magnification images of HE and immunohistochemistry for CD68, CTL1, and ChoKα in stable and unstable plaques. CTL1 expression in CD68-positive macrophages was higher in unstable lesions than in stable lesions. ChoKα expression in macrophage-rich areas was comparable between stable and unstable lesions. Scale bars represent 20 μm. (C) Immunopositive areas for α-SMA, CD68, CTL1, and ChoKα in human coronary arteries. α-SMA and CD68 immunopositive areas were measured in the whole arterial wall. CTL1- and ChoKα-immunopositive areas were measured in the most densely stained area in each plaque. α-SMA immunopositive area in early lesions (50.2 ± 15.9%), stable lesions (34.2 ± 12.5%), or unstable lesions (17.4 ± 11.8%). CD68 immunopositive area in early lesions (0.04 ± 0.05%), stable lesions (0.6 ± 1.0%), or unstable lesions (6.2 ± 4.7%). CTL1 immunopositive areas in early lesions (0.4 ± 0.4%), stable lesions (0.7 ± 0.7%), or unstable lesions (1.6 ± 1.3%). ChoKα immunopositive area in early lesions (1.4 ± 0.7%), stable lesions (1.5 ± 1.2%), or unstable lesions (1.9 ± 1.2%). Data are represented as mean ± standard deviation, and individual data points and median for each group. Data were analyzed using Kruskal-Wallis tests followed by Dunn’s multiple comparison tests.

Immunohistochemistry revealed that the early lesions predominantly comprised α-SMA-positive SMCs. Stable lesions exhibited the formation of fibrous cap atheroma with or without calcification and the accumulation of CD68-positive macrophages under the thick fibrous cap. Unstable lesions were large atherosclerotic plaques rich in macrophages (Fig 5A). Choline transporter-like protein 1 expression was observed predominantly in macrophage-rich areas of unstable plaques, whereas ChoKα expression was detected in spindle and macrophage-like cells in all lesion types (Fig 5A and 5B). In coronary plaques, CTL1 immunopositive areas from unstable lesions were significantly larger than those in early (p < 0.001) or stable lesions (p < 0.05) (Fig 5C). In contrast, ChoKα-immunopositive areas in coronary plaques were not significantly different among the lesions (Fig 5C).

### Effects of atherosclerotic microenvironment on choline levels in PBMC-derived macrophages

**Fig 6:**
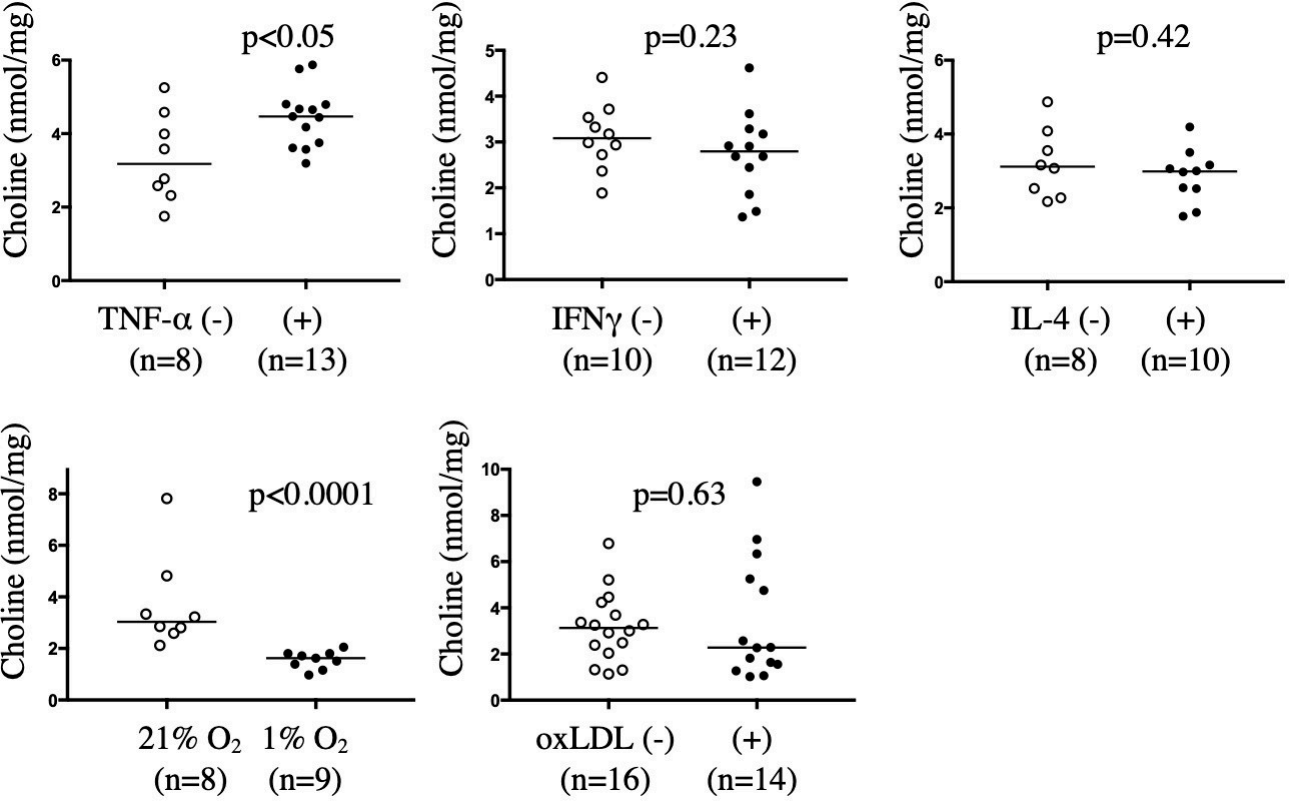
Effect of TNF-α, INFγ, IL-4, hypoxia, and oxidized low-density lipoprotein (oxLDL) on choline levels in peripheral blood mononuclear cell (PBMC)-derived macrophages. Choline levels in cultured macrophages were measured 3 days post-stimulation with TNF-α (10 ng/mL), IFNγ (50 ng/mL), and IL-4 (20 ng/mL), 1 day after exposure to hypoxia, or 2 days after administration of oxLDL (50 μg/mL). Choline levels in non-stimulated macrophages (3.4 ± 1.1 nmol/mg) or TNF-α-stimulated macrophages (4.4 ± 0.8 nmol/mg). Choline levels in non-stimulated macrophages (3.1 ± 0.7 nmol/mg) or IFNγ-stimulated macrophages (2.7 ± 0.9 nmol/mg). Choline levels in non-stimulated macrophages (3.2 ± 0.9 nmol/mg) or IL-4-stimulated macrophages (2.9 ± 0.7 nmol/mg). Choline levels in normoxic macrophages (21% O_2_, 3.7 ± 1.7 nmol/mg) or hypoxic macrophages (1% O_2_, 1.6 ± 0.3 nmol/mg). Choline levels in non-stimulated macrophages (3.2 ± 1.5 nmol/mg) or oxLDL-stimulated macrophages (3.4 ± 2.6 nmol/mg). Data are represented as mean ± standard deviation, and individual data points and median for each group. Data were analyzed using Mann-Whitney U tests.

The effects of atherosclerotic stimuli on choline levels were assessed using human PBMC-derived macrophages *in vitro*. Intracellular choline levels in macrophages stimulated with TNF-α, INFγ, IL-4, oxLDL, or exposed to hypoxia (1% O_2_) were measured using LC-MS/MS. Intracellular choline levels were significantly upregulated in macrophages stimulated with TNF-α but were downregulated in macrophages exposed to hypoxia. Administration of INFγ, IL-4, or oxLDL did not affect the intracellular choline levels (Fig 6).

### Effects of choline and CTL1 expression on *CTL1*, *TNF-α*, *IL-6*, and *MMP9* mRNA levels in PBMC-derived macrophages and human coronary artery smooth muscle cells (HCASMCs)

**Fig 7:**
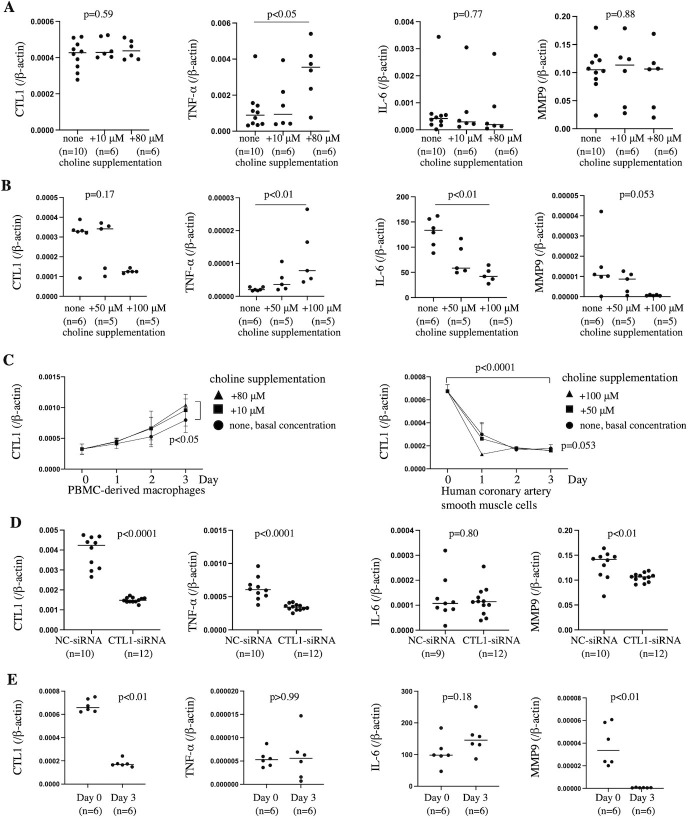
Effects of choline and CTL1 expression on *CTL1*, *TNF-α*, *IL-6*, and *MMP9* mRNA levels in PBMC-derived macrophages and HCASMCs. Total RNA from PBMC-derived macrophages and HCASMCs was isolated and reverse-transcribed into cDNA. Quantitative RT-PCR was performed for *CTL1*, *TNF-α*, *IL-6*, *MMP9*, and *β-actin*. Target gene expression levels were normalized to those of *β-actin*. (A) Effects of choline on *CTL1*, *TNF-α*, *IL-6*, and *MMP9* mRNA levels in PBMC-derived macrophages on day 1. *CTL1* mRNA levels without choline supplementation (0.0004 ± 0.00007), 10 μM choline supplementation (0.0004 ± 0.00005), or 80 μM choline supplementation (0.0004 ± 0.00004). *TNF-α* mRNA levels without choline supplementation (0.001 ± 0.001), 10 μM choline supplementation (0.002 ± 0.001), or 80 μM choline supplementation (0.003 ± 0.002). *IL-6* mRNA levels without choline (0.0007 ± 0.0009), 10 μM choline supplementation (0.0008 ± 0.001), or 80 μM choline supplementation (0.0007 ± 0.001). *MMP9* mRNA levels without choline supplementation (0.10 ± 0.04), on 10 μM choline supplementation (0.10 ± 0.05) or on 80 μM choline supplementation (0.10 ± 0.05). The data are represented as mean ± standard deviation, and individual data points and median for each group. Data were analyzed using Kruskal-Wallis tests followed by Dunn’s multiple comparisons tests. (B) Effects of choline on *CTL1*, *TNF-α*, *IL-6*, and *MMP9* mRNA levels in HCASMCs on day 1. *CTL1* mRNA levels without choline supplementation (0.0003 ± 0.0001), 50 μM choline supplementation (0.0003 ± 0.0001), or 100 μM choline supplementation (0.0001 ± 0.000009). *TNF-α* mRNA levels without choline supplementation (0.000002 ± 0.0000005), 50 μM choline supplementation (0.000005 ± 0.000003), or 100 μM choline supplementation (0.00001 ± 0.000008). *IL-6* mRNA levels without choline supplementation (129.6 ± 26.2), on 50 μM choline supplementation (75.1 ± 26.9) or on 100 μM choline supplementation (44.6 ± 12.8). *MMP9* mRNA levels without choline supplementation (0.00001 ± 0.00001), with 50 μM choline supplementation (0.000007 ± 0.000005), or with 100 μM choline supplementation (0.0000007 ± 0.0000003). The data are represented as mean ± standard deviation, and individual data points and median for each group. Data were analyzed using Kruskal-Wallis tests followed by Dunn’s multiple comparisons tests. (C) Effects of choline on *CTL1* mRNA levels in PBMC-derived macrophages and HCASMCs. These data are represented as mean ± standard deviation for each group and were analyzed using two-way ANOVA followed by Tukey’s multiple comparison tests. (D) *CTL1*, *TNF-α*, *IL-6*, *MMP9* mRNA levels in PBMC-derived macrophages 3 days after 80 μM choline supplementation and 3 days after transfection of negative control (NC) or small interfering RNA (siRNA). *CTL1* mRNA level after transfection of NC-siRNA (0.004 ± 0.0008) or CTL1-siRNA (0.001 ± 0.0001). *TNF-α* mRNA level after transfection of NC-siRNA (0.0006 ± 0.0002) or CTL1-siRNA (0.0003 ± 0.00005). *IL-6* mRNA level after transfection of NC-siRNA (0.0001 ± 0.00008) or CTL1-siRNA (0.0001 ± 0.00006). *MMP9* mRNA level after transfection of NC-siRNA (0.1 ± 0.03) or CTL1-siRNA (0.1 ± 0.008). Data are represented as mean ± standard deviation, and individual data points and median for each group. Data were analyzed using Mann-Whitney U tests. (E) *TNF-α*, *IL-6*, and *MMP9* mRNA levels according to CTL1 mRNA expression in HCASMCs after 0 and 3 days. *CTL1* mRNA level at day 0 (0.0007 ± 0.00005) or at day 3 (0.0002 ± 0.00003). *TNF-α* mRNA level at day 0 (0.000006 ± 0.000002) or at day 3 (0.000006 ± 0.000005). *IL-6* mRNA level at day 0 (107.0 ± 40.7) or at day 3 (153.3 ± 50). *MMP9* mRNA level at day 0 (0.00004 ± 0.00002) or at day 3 (0.0000006 ± 0.0000002). Data are represented as mean ± standard deviation, and individual data points and median for each group. Data were analyzed using Mann-Whitney U tests.

We assessed the effects of choline on *CTL1*, *TNF-α*, *IL-6*, and *MMP9* mRNA levels in PBMC-derived macrophages and HCASMCs. We added 0, 10, and 80 μM choline to the macrophage basal medium (RPMI-1640) used to culture PBMC-derived macrophages and 0, 50, and 100 μM choline to the SMC basal medium (SmBM) used to culture HCASMCs. Supplementation with 80 μM choline increased *TNF-α* levels but did not affect the expression of *CTL1*, *IL-6*, and *MMP9* in PBMC-derived macrophages after 1 day (Fig 7A). Supplementation with 100 μM choline increased *TNF-α* levels and decreased *IL-6* levels in HCASMCs after 1 day. Supplementation with 100 μM choline tended to decrease *CTL1* and *MMP9* levels; however, the difference was not significant (Fig 7B).

The *CTL1* level increased in a time-dependent manner in PBMC-derived macrophages at all choline concentrations. Choline (80 μM) supplementation enhanced *CTL1* levels after 3 days compared with the basal concentration of choline (Fig 7C).

To examine the effects of CTL1 on mRNA expression of *TNF-α*, *IL-6*, and *MMP9* in PBMC-derived macrophages, we transiently transfected with *CTL1* siRNA in the *CTL1* upregulated macrophages. The transfection of *CTL1* siRNA significantly decreased *CTL1* mRNA levels, compared with the transfection of negative control siRNA. In this condition, the transfection of *CTL1* siRNA significantly decreased *TNF-α* and *MMP9* mRNA levels in the macrophages. The transfection of *CTL1* siRNA did not affect *IL-6* level in the macrophages (Fig 7D).

Levels of *CTL1* decreased in a time-dependent manner in HCASMCs. However, choline concentrations did not affect *CTL1* levels (Fig 7C). Therefore, we compared *TNF-α*, *IL-6*, and *MMP9* levels in HCASMCs between days 0 and 3 under basal choline concentrations. Levels of *MMP9* were lower in HCASMCs on day 3 than on day 0, similar to *CTL1* levels. *TNF-α* and *IL-6* levels did not differ between the HCASMCs on days 0 and 3 (Fig 7E).

## Discussion

Choline levels in arteries with macrophage-rich neointima were significantly higher than those in arteries with SMC-rich neointima, non-injured arteries, and cardiac tissues in rabbits. Additionally, choline levels were positively correlated with areas that were immunopositive for macrophages and *TNF*-α and *MMP9* mRNA levels. Macrophage CTL1 expression in unstable human coronary plaques was upregulated compared to that in early or stable lesions. Meanwhile, ChoKα expression was comparable among these lesions. TNF-α and hypoxia modulated intercellular choline levels in human PBMC-derived macrophages. Extracellular choline levels affect the expression of *TNF*-α and *CTL1* in macrophages and the expression of *TNF*-α and *IL-6* in HCASMCs.

Choline, an essential nutrient, is an important source of acetylcholine, cell membrane phospholipids, and methyl group metabolites [16]. Arterial choline levels were abundant in the macrophage-rich neointima and were correlated with the foamy macrophage content. Because intracytoplasmic lipid droplets contain a neutral lipid core covered with a phospholipid monolayer [17], choline may serve as a component of the phospholipid membrane of lipid droplets in foamy macrophages. Additionally, choline levels correlated with the expression of *TNF-α* and *MMP9*, but not with *TF* and *IL-6* expression. These results suggest that vascular choline levels indicate plaque inflammation and instability, but not procoagulant activity. *IL-6* is a pleiotropic cytokine with proinflammatory and anti-inflammatory properties [18].

The expression of the choline transporter CTL1 in macrophages is upregulated in unstable plaques in coronary arteries. Reportedly, this is the first study to demonstrate the upregulation of CTL1 in human coronary arteries. In contrast to the macrophage content, CTL1 expression in stable lesions was not upregulated when compared to that in early lesions. This result suggests that the factors in unstable plaques may affect CTL1 expression in macrophages. Based on our *in vitro* study, choline level may be a regulator of *CTL1* expression in macrophages, but not in SMCs. CTL1 expression in human coronary arteries and the association of choline / CTL1 levels with *TNF-*α and *MMP9* expression in rabbits and cultured macrophages suggest that macrophage choline metabolism is associated with plaque instability.

In an atherosclerotic microenvironment, TNF-α administration affects intercellular choline levels in macrophages *in vitro*. Several studies have examined choline uptake and inflammatory responses in macrophages. Snider et al. [19] demonstrated that lipopolysaccharide stimulation of mouse bone marrow-derived macrophages increases choline uptake via the upregulation of CTL1, resulting in the upregulation of phosphatidylcholine biosynthesis. Sanchez-Lopez et al. [20] reported that toll-like receptor activation by lipopolysaccharide in mouse bone marrow-derived macrophages enhances choline uptake via upregulation of CTL1 and that ChoKα-mediated choline phosphorylation contributes to IL-1β production. This study did not use lipopolysaccharide as a stimulus because it is uncommon in atherosclerotic microenvironments. The findings of this study on rabbits and PBMC-derived macrophages (Figs 4, 6 and 7A) suggest that TNF-α is a candidate for increasing cellular choline levels in the atherosclerotic microenvironment, and that increased extracellular choline levels modulate plaque inflammation via *TNF-α* expression. In addition, the macrophage CTL1 expression itself may play a role in plaque instability (Fig 7D).

Hypoxia downregulated intracellular choline levels in macrophages in this study. The effects of hypoxia on intracellular choline levels are controversial. Glunde et al. reported that hypoxia increases ChoKα expression and intracellular choline levels in human PC-3 prostate cancer cells by promoting the binding of hypoxia-inducible factor 1α to hypoxia-responsive elements [21]. However, Bansal et al. reported that hypoxia suppresses radiolabeled choline phosphorylation and intracellular accumulation in 9L glioma cell allografts [22]. Similarly, hypoxia downregulates ChoKα expression and choline phosphorylation in PC-3 cells [23]. These results suggest that hypoxia differentially affects choline levels in different cell types.

High concentrations of choline increased *TNF-α* mRNA expression and decreased *IL-6* mRNA expression in HCASMCs *in vitro*. These results suggest that choline can modulate inflammatory responses in SMCs. He et al. reported that choline inhibits angiotensin II-induced vascular SMC phenotypic switching and migration, while significantly inhibiting IL-6 protein expression [24]. Our results on *IL-6* expression were comparable to those of the previous report. In addition, *CTL1* and *MMP9* mRNA expression was similarly reduced in HCASMCs (Fig 7E). These results imply a relationship between *CTL1* expression in SMCs and matrix degradation in atherosclerotic plaques.

Choline and guanine levels in macrophage-rich neointima were significantly higher than those in SMC-rich neointima and myocardium. Ooga et al. [25] measured the levels of 335 metabolites in the plasma and tissues, including the aorta and heart, in Watanabe heritable hyperlipidemic (WHHL) and healthy control rabbits using CE-TOFMS. These authors reported that the levels of central carbon metabolites, amino acids, and purine metabolites in cardiac tissues were upregulated compared with those in the atherosclerotic arteries of WHHL rabbits. These results are consistent with those of the present study. Several clinical studies demonstrated that carotid atherosclerotic plaques can be identified using positron emission tomography imaging with ^18^F-fluorodeoxyglucose (^18^F-FDG) [26]. The uptake of ^18^F-FDG is closely correlated with plaque macrophage content [27]. However, ^18^F-FDG uptake by coronary plaques can be obscured by myocardial uptake [28]. Previous studies demonstrated the uptake of radiolabeled choline in murine models of atherosclerosis [29, 30] and reported a correlation between choline uptake and lipid and macrophage content [30]. Thus, our choline-targeting approach may be a useful imaging tool for detecting macrophage-rich plaques in coronary arteries.

## Limitations

This study has several limitations. Metabolic changes over time were not assessed in the rabbit model of atherosclerosis. Additionally, the rabbit coronary arteries were not subjected to metabolomic analyses. Furthermore, choline levels in the human coronary arteries were not examined.

## Conclusion

These results suggest alterations in choline metabolism in macrophage-rich atherosclerotic lesions and unstable plaques. Choline and CTL1 may be markers of plaque instability.

## Supporting information

S1 Table Primer sequences used for RT-PCR of rabbit genes. (PDF)

S2 Table Primer sequences used for RT-PCR of human genes. (PDF)

S3 Table Principal components 1 and 2 in rabbits fed a conventional diet. (PDF)

S4 Table Hierarchical clustering analysis of arterial and cardiac metabolites in rabbits fed a conventional diet. (PDF)

S5 Table Principal component 1 and 2 from rabbits fed a 0.5% cholesterol diet. (PDF)

S6 Table Hierarchical clustering analysis of arterial and cardiac metabolites in rabbits fed a 0.5% cholesterol diet. (PDF)

S7 Table Arterial and cardiac metabolite levels in rabbits fed a 0.5% cholesterol diet. (PDF)

S8 Table Relative amount of arterial and cardiac metabolites in rabbits fed a 0.5% cholesterol diet. (PDF)

S9 Table Arterial and cardiac metabolite levels in rabbits fed a conventional diet. (PDF)

S10 Table Clinical background of autopsy cases. (PDF)
