# Supplementary material for: Altered choline level in atherosclerotic lesions: Upregulation of choline transporter-like protein 1 in human coronary unstable plaque
Source: PLoS One. 2023 Feb 17;18(2):e0281730. doi: 10.1371/journal.pone.0281730 (PMC9937458; doi:10.1371/journal.pone.0281730)
Supplement: S1 Table — (PDF) [file pone.0281730.s001.PDF]

Supplementary Table 1. Primer sequences used for RT-PCR of rabbit genes

Gene	Primer sequence	
rabbit tissue factor (TF)	Forward	5'- GGGCAGGGCCAGGGAGATGTTCTTC -3'
	Reverse	5'- GCCCTCGCCTTTCTGCACTTG -3'
rabbit interleukin-6 (IL-6)	Forward	5'- GAAAACACCAGGGTCAGCAT -3'
	Reverse	5'- CAGCCACTGGTTTTTCTGCT-3'
rabbit tissue necrosis factor-alpha (TNF-α)	Forward	5'- CTCCTACCCGAACAAGGTCA -3'
	Reverse	5'- CGGTCACCCTTCTCCAACT -3'
rabbit matrix metaroproteinase (MMP-9)	Forward	5'- CTTCCAACTTTGACAGCGACA -3'
	Reverse	5'- GGAGTGATCCAAGCCCAGTG -3'
rabbit Hydroxymethylbilane synthase (HMBS)	Forward	5'- GGCAACGGCAATGACGAGGCT -3'
	Reverse	5'- TGTCCGTCTGGATGCGAGCCA -3'
