# Supplementary material for: Altered choline level in atherosclerotic lesions: Upregulation of choline transporter-like protein 1 in human coronary unstable plaque
Source: PLoS One. 2023 Feb 17;18(2):e0281730. doi: 10.1371/journal.pone.0281730 (PMC9937458; doi:10.1371/journal.pone.0281730)
Supplement: S2 Table — (PDF) [file pone.0281730.s002.PDF]

Supplementary Table 2. Primer sequences used for RT-PCR of human genes

Gene	Primer sequence	
human choline transporter-like 1, transcript variant 3 (CTL-1)	Forward	5'-CTTTGCGAGTGGCTACCATCA-3'
	Reverse	5'-TGAGCAGCATAATCCCAGCTAAAC-3'
human tissue necrosis factor-alpha (TNF-α)	Forward	5’-TGCTTGTTCCTCAGCCTCTT-3’
	Reverse	5’-CAGAGGGCTGATTAGAGAGAGGT-3’
human interleukin-6 (IL-6)	Forward	5’-AAGCCAGAGCTGTGCAGATGAGTA -3’
	Reverse	5’-TGTCCTGCAGCCACTGGTTC-3’
human matrix metaroproteinase (MMP-9)	Forward	5’- TGGCACCACCACAACATCAC-3’
	Reverse	5’- GCAAAGGCGTCGTCAATCA-3’
human beta-actin (β-actin)	Forward	5'-TGGCACCCAGCACAATGAA-3'
	Reverse	5'-CTAAGTCATAGTCCGCCTAGAAGCA-3'
