# Supplementary material for: Altered choline level in atherosclerotic lesions: Upregulation of choline transporter-like protein 1 in human coronary unstable plaque
Source: PLoS One. 2023 Feb 17;18(2):e0281730. doi: 10.1371/journal.pone.0281730 (PMC9937458; doi:10.1371/journal.pone.0281730)
Supplement: S3 Table — (PDF) [file pone.0281730.s003.PDF]

Supplementary table 3. Principal components 1 and 2 in rabbits fed a conventional diet

ID	HMT DB †			*m/z*	MT/RT	PC1	PC2
	Compound name	KEGG ID	HMDB ID				
A_0001	Glyoxylic acid	C00048	HMDB00119	72.993	12.45	-1.7E-02	8.8E-03
A_0003	Pyruvic acid	C00022	HMDB00243	87.008	13.71	-2.3E-02	-3.4E-03
A_0004	Lactic acid	C00186,C0025	HMDB00190,H	89.024	11.18	8.3E-02	1.3E-02
A_0005	3-Hydroxybutyric acid	C01089,C0319	HMDB00011,H	103.040	9.79	7.3E-02	2.4E-02
A_0006	2-Hydroxybutyric acid	C05984	HMDB00008	103.040	10.12	4.1E-02	5.0E-02
A_0007	Glyceric acid	C00258	HMDB00139,H	105.019	10.88	-5.3E-02	1.2E-01
A_0008	Fumaric acid	C00122	HMDB00134	115.003	27.82	8.4E-02	3.6E-03
A_0010	Hexanoic acid	C01585	HMDB00535	115.076	9.19	1.2E-02	8.5E-02
A_0011	*N*-Acetylglycine	No ID	HMDB00532	116.034	9.94	-2.7E-02	-9.8E-03
A_0012	Succinic acid	C00042	HMDB00254	117.019	22.73	7.6E-02	1.5E-02
A_0013	2-Hydroxyvaleric acid	No ID	HMDB01863	117.056	9.19	5.7E-02	2.5E-03
A_0014	Benzoic acid	C00180	HMDB01870	121.029	10.31	-2.3E-02	9.0E-02
A_0015	Isethionic acid	C05123	HMDB03903	124.991	12.00	8.5E-02	5.4E-03
A_0016	5-Oxoproline	C01879	HMDB00267	128.035	9.73	8.4E-02	2.3E-02
A_0017	4-Methyl-2-oxovaleric acid<br>3-Methyl-2-oxovaleric acid	C00233<br>C00671,C034<br>65	HMDB00695<br>HMDB00491	129.054	9.96	-3.9E-02	4.9E-02
A_0018	5-Oxohexanoic acid	C02129	No ID	129.055	9.44	-2.6E-02	3.4E-02
A_0019	Heptanoic acid	No ID	HMDB00666	129.091	8.90	-3.7E-02	1.1E-01
A_0020	*N*-Acetylalanine	No ID	HMDB00766	130.051	9.20	-6.1E-02	7.8E-02
A_0021	6-Hydroxyhexanoic acid	C06103	No ID	131.071	8.55	4.8E-02	2.3E-02
A_0022	Malic acid	C00149,C0049	HMDB00156,H	133.014	23.19	8.4E-02	4.5E-03
A_0023	Threonic acid	C01620	HMDB00943	135.031	9.19	7.3E-02	-7.3E-03
A_0024	Ethanolamine phosphate	C00346	HMDB00224	140.011	8.18	-3.7E-02	9.5E-02
A_0025	Octanoic acid	C06423	HMDB00482	143.107	8.59	-1.5E-02	8.3E-02
A_0027	Adipic acid	C06104	HMDB00448	145.050	16.14	-4.4E-02	-2.6E-02
A_0028	2-Hydroxyglutaric acid	C02630,C0108	HMDB00606,H	147.029	16.95	8.4E-02	3.8E-03
A_0029	Pelargonic acid	C01601	HMDB00847	157.123	8.29	4.1E-02	1.1E-01
A_0030	Pimelic acid	C02656	HMDB00857	159.065	14.75	-2.7E-02	3.2E-02
A_0031	8-Hydroxyoctanoic acid	No ID	No ID	159.102	8.04	6.9E-02	9.9E-03
A_0032	Terephthalic acid	C06337	HMDB02428	165.018	17.58	8.5E-02	6.3E-03
A_0033	Quinolinic acid	C03722	HMDB00232	166.014	17.08	-3.2E-02	7.1E-04
A_0034	XA0012	-	-	166.018	9.63	7.3E-02	3.2E-02
A_0035	Phosphoenolpyruvic acid	C00074	HMDB00263	166.974	23.64	-6.9E-02	8.9E-02
A_0036	Uric acid	C00366	HMDB00289	167.021	9.11	3.7E-02	7.9E-02
A_0037	Dihydroxyacetone phosphate	C00111	HMDB01473	168.990	13.25	6.9E-02	4.2E-04
A_0039	Glycerol 3-phosphate	C00093	HMDB00126	171.006	12.64	8.5E-02	7.1E-03
A_0040	Decanoic acid	C01571	HMDB00511	171.138	8.18	-2.9E-02	1.2E-01
A_0041	Isovalerylalanine	No ID	HMDB00747	172.098	8.26	-7.2E-02	2.8E-02
A_0042	*cis*-Aconitic acid	C00417	HMDB00072	173.008	30.86	-2.8E-04	-1.4E-01
A_0043	Suberic acid	C08278	HMDB00893	173.081	13.71	-7.0E-02	5.5E-02
A_0044	*N*-Acetylaspartic acid	C01042	HMDB00812	174.040	15.37	-5.0E-02	8.9E-02
A_0045	Hippuric acid	C01586	HMDB00714	178.050	8.35	6.4E-02	1.1E-02
A_0046	2-Phosphoglyceric acid	C00631	HMDB03391	184.984	21.27	-7.6E-02	4.8E-02
A_0047	3-Phosphoglyceric acid	C00197	HMDB00807	184.985	21.31	-5.3E-02	6.7E-02
A_0048	XA0017	-	-	186.113	7.98	-4.4E-02	7.7E-02

A_0049	Azelaic acid	C08261	HMDB00784	187.097	12.92	-6.8E-02	3.9E-02
A_0050	10-Hydroxydecanoic acid	C02774	No ID	187.133	7.69	7.7E-02	7.8E-03
A_0051	Isocitric acid	C00311	HMDB00193	191.018	31.70	1.5E-02	-1.5E-01
A_0052	XA0019	-	-	191.019	8.46	-2.1E-02	8.8E-02
A_0053	Citric acid	C00158	HMDB00094	191.019	28.49	-8.6E-03	-1.3E-01
A_0054	Phenaceturic acid	C05598	HMDB00821	192.066	8.12	7.0E-02	1.6E-02
A_0055	Gluconic acid	C00257	HMDB00625	195.050	8.40	-6.0E-02	-5.3E-02
A_0056	Erythrose 4-phosphate	C00279,C0360	HMDB01321	198.999	12.26	-2.9E-02	9.5E-02
A_0057	Sebacic acid	C08277	HMDB00792	201.112	12.28	-7.4E-02	4.2E-02
A_0058	Mucic acid	C00879,C0180	HMDB00639	209.029	15.16	-3.4E-02	1.3E-01
A_0059	Phosphocreatine	C02305	HMDB01511	210.028	12.84	-3.3E-02	-9.8E-02
A_0060	3-Indoxylsulfuric acid	No ID	HMDB00682	212.003	9.71	7.2E-02	4.2E-02
A_0061	*O*-Succinylhomoserine	C01118	No ID	218.069	13.01	-2.3E-02	1.3E-01
A_0062	Pantothenic acid	C00864	HMDB00210	218.103	7.77	2.2E-02	8.0E-02
A_0063	Myristoleic acid	C08322	HMDB02000	225.185	7.62	-2.7E-02	1.4E-01
A_0064	XA0027	-	-	227.200	7.37	4.6E-02	-1.9E-03
A_0065	Ribulose 5-phosphate	C00199,C0110	HMDB00618	229.011	11.36	5.7E-02	1.2E-01
A_0066	Ribose 5-phosphate	C00117	HMDB01548	229.011	10.92	-2.8E-02	1.4E-01
A_0067	XA0033	-	-	242.079	7.75	8.2E-02	4.3E-02
A_0068	Biotin	C00120	HMDB00030	243.081	7.55	8.5E-02	1.6E-03
A_0069	Glucosamine 6-phosphate	C00352	HMDB01254	258.038	8.80	4.6E-02	-1.1E-02
A_0070	Glucose 1-phosphate	C00103	HMDB01586	259.021	10.44	5.8E-02	-2.7E-02
A_0071	Glucose 6-phosphate	C00668,C0117	HMDB01401	259.021	10.12	6.7E-02	-1.3E-02
A_0072	*myo*-Inositol 2-phosphate	No ID	No ID	259.021	10.86	4.5E-02	1.5E-02
A_0073	Fructose 6-phosphate	C05345,C0008	HMDB00124	259.021	10.15	7.1E-02	-1.0E-02
A_0074	*myo*-Inositol 1-phosphate<br>*myo*-Inositol 3-phosphate	C01177<br>C04006	HMDB00213<br>HMDB06814	259.021	10.58	4.5E-02	1.1E-01
A_0075	2,3-Diphosphoglyceric acid	C01159	HMDB01294	264.951	19.84	3.2E-03	-7.2E-02
A_0076	6-Phosphogluconic acid	C00345	HMDB01316	275.016	15.61	-2.7E-02	2.2E-02
A_0077	Xanthosine	C01762	HMDB00299	283.066	7.80	-4.7E-02	1.3E-01
A_0078	Sedoheptulose 7-phosphate	C05382	HMDB01068	289.032	9.89	-3.1E-02	1.5E-01
A_0079	*N*-Acetylglucosamine 1-phosphate	C04256	HMDB01367	300.047	9.96	-5.6E-02	1.1E-01
A_0080	*N*-Acetylglucosamine 6-phosphate	C00357	HMDB01062	300.048	9.37	-3.4E-02	1.4E-01
A_0081	*N*-Acetylneuraminic acid	C00270	HMDB00230	308.098	7.26	7.8E-02	3.4E-02
A_0082	Ribulose 1,5-diphosphate	C01182	No ID	308.978	17.01	-4.3E-02	4.6E-02
A_0084	CMP	C00055	HMDB00095	322.041	9.83	7.5E-02	-1.8E-02
A_0085	UMP	C00105	HMDB00288	323.027	10.06	7.8E-02	-1.4E-02
A_0086	cAMP	C00575	HMDB00058	328.044	7.43	8.4E-02	4.0E-04
A_0087	Fructose 1,6-diphosphate	C00354	HMDB01058	338.988	15.39	7.2E-02	-1.5E-02
A_0088	cGMP	C00942	HMDB01314	344.039	7.51	6.2E-02	-4.1E-03
A_0089	AMP	C00020	HMDB00045	346.055	9.50	8.4E-02	3.0E-03
A_0090	IMP	C00130	HMDB00175	347.038	9.77	5.4E-02	-2.0E-02
A_0091	GMP	C00144	HMDB01397	362.049	9.37	8.4E-02	-9.1E-03
A_0092	CoA_divalent	C00010	HMDB01423	382.549	10.95	8.1E-02	-3.6E-03
A_0093	PRPP	C00119	HMDB00280	388.943	16.86	3.3E-02	-1.0E-02
A_0094	FAD_divalent	C00016	HMDB01248	391.571	7.98	8.5E-02	7.3E-03
A_0096	CDP	C00112	HMDB01546	402.010	11.84	3.0E-02	-9.7E-02
A_0097	UDP	C00015	HMDB00295	402.995	12.09	1.2E-02	-1.0E-01
A_0098	Acetyl CoA_divalent	C00024	HMDB01206	403.554	10.51	4.8E-02	-1.3E-02

A_0099	Cholic acid	C00695	HMDB00619	407.280	7.01	-3.1E-02	3.0E-02
A_0100	ThPP	C00068	HMDB01372	423.029	7.87	8.5E-02	8.9E-04
A_0102	3',5'-ADP	C00054	HMDB00061	426.021	14.32	-6.7E-02	2.0E-02
A_0103	ADP	C00008	HMDB01341	426.021	11.23	8.0E-02	-1.5E-02
A_0104	GDP	C00035	HMDB01201	442.016	10.99	7.3E-02	-6.0E-02
A_0105	XA0065	-	-	445.053	7.01	4.3E-02	6.4E-02
A_0106	Octanoyl CoA_divalent	C01944	HMDB01070	445.602	9.53	5.8E-02	7.7E-03
A_0107	FMN	C00061	HMDB01520	455.098	8.25	8.4E-02	-8.8E-04
A_0108	Adenylosuccinic acid	C03794	HMDB00536	462.066	14.16	7.7E-02	-1.9E-03
A_0111	CTP	C00063	HMDB00082	481.978	13.09	-1.3E-02	-9.5E-02
A_0112	UTP	C00075	HMDB00285	482.961	13.06	3.9E-02	-6.0E-02
A_0113	CDP-choline	C00307	HMDB01413	487.099	6.83	7.9E-02	3.9E-02
A_0115	ATP	C00002	HMDB00538	505.988	12.19	6.3E-02	-7.9E-03
A_0116	GTP	C00044	HMDB01273	521.983	11.83	7.4E-02	-2.5E-02
A_0117	ADP-ribose	C00301	HMDB01178	558.064	8.67	8.3E-02	5.7E-03
A_0118	UDP-glucose<br>UDP-galactose	C00029<br>C00052	HMDB00286<br>HMDB00302	565.048	8.82	7.7E-02	-2.6E-03
A_0119	UDP-glucuronic acid	C00167	HMDB00935	579.026	11.40	3.8E-02	-5.5E-02
A_0120	ADP-glucose<br>GDP-fucose	C00498<br>C00325	HMDB06557<br>HMDB01095	588.075	8.46	8.5E-02	-4.8E-03
A_0121	GDP-galactose<br>GDP-mannose	C02280<br>C00096	No ID<br>HMDB01163	604.070	8.43	7.2E-02	-9.8E-03
A_0122	UDP-*N*-acetylglucosamine	C00043	HMDB00290	606.075	8.62	7.8E-02	-1.6E-02
A_0123	CMP-*N*-acetylneuraminate	C00128	HMDB01176	613.140	8.08	8.5E-02	1.3E-03
A_0124	NAD^+	C00003	HMDB00902	662.104	6.60	1.0E-03	-2.9E-02
A_0125	$NADP^+$	C00006	HMDB00217	742.070	9.75	-5.5E-02	-5.1E-02
C_0001	Urea	C00086	HMDB00294	61.040	21.79	8.4E-02	1.7E-02
C_0002	Ethanolamine	C00189	HMDB00149	62.060	6.53	3.7E-03	1.5E-01
C_0003	XC0001	-	-	72.081	6.56	8.0E-02	-2.1E-02
C_0004	Gly	C00037	HMDB00123	76.040	8.55	-2.0E-02	1.0E-01
C_0005	Trimethylamine *N*-oxide	C01104	HMDB00925	76.076	6.79	3.9E-02	1.9E-02
C_0006	Morpholine	C14452	HMDB31581	88.075	6.83	8.5E-02	4.3E-03
C_0007	Putrescine	C00134	HMDB01414	89.108	4.84	6.7E-02	6.0E-02
C_0008	Sarcosine	C00213	HMDB00271	90.055	9.73	-6.0E-02	5.7E-02
C_0009	Ala	C00041,C0013	HMDB00161,H	90.055	9.28	8.1E-02	1.7E-02
C_0010	β-Ala	C00099	HMDB00056	90.055	7.55	-5.6E-02	1.0E-01
C_0011	Glycerol	C00116	HMDB00131	93.055	22.74	-3.7E-02	-2.1E-02
C_0012	Homoserinelactone	No ID	No ID	102.055	7.28	6.2E-02	9.2E-03
C_0013	*N*,*N*-Dimethylglycine	C01026	HMDB00092	104.072	11.27	6.1E-02	-6.2E-03
C_0014	2-Aminoisobutyric acid	C03665	HMDB01906	104.071	9.92	6.0E-02	9.8E-02
C_0015	GABA	C00334	HMDB00112	104.071	7.91	-6.3E-02	9.9E-02
C_0016	Choline	C00114	HMDB00097	104.107	7.06	-4.2E-02	1.3E-01
C_0017	Ser	C00065,C0071	HMDB00187,H	106.050	10.27	7.6E-02	5.3E-02
C_0018	Diethanolamine	C06772	HMDB04437	106.086	7.87	-1.7E-02	1.6E-02
C_0019	Hypotaurine	C00519	HMDB00965	110.027	18.68	6.7E-02	4.1E-02
C_0021	Histamine	C00388	HMDB00870	112.086	4.91	6.8E-02	-3.0E-03
C_0022	Uracil	C00106	HMDB00300	113.035	22.80	2.3E-02	1.5E-01
C_0023	Creatinine	C00791	HMDB00562	114.066	7.51	8.3E-02	1.4E-02
C_0024	Pro	C00148,C0076	HMDB00162,H	116.070	11.12	7.6E-02	5.1E-02
C_0025	Guanidoacetic acid	C00581	HMDB00128	118.061	8.48	8.2E-02	4.7E-03
C_0026	Val	C00183,C0641	HMDB00883	118.086	10.31	6.7E-02	7.4E-02

C_0027	Betaine	C00719	HMDB00043	118.086	11.59	8.5E-02	7.8E-03
C_0028	Thr	C00188,C0082	HMDB00167	120.065	10.82	7.5E-02	6.7E-02
C_0029	Homoserine	C00263	HMDB00719	120.066	10.38	6.6E-02	2.5E-02
C_0030	Betaine aldehyde_+H_2O	C00576	HMDB01252	120.102	7.66	-2.0E-02	1.4E-02
C_0031	Anserine_divalent	C01262	HMDB00194	121.068	7.04	8.3E-02	-2.3E-02
C_0032	Cys	C00097,C0073	HMDB00574,H	122.027	11.59	-4.1E-02	-4.2E-02
C_0033	2-Amino-2-(hydroxymethyl)-1,3-propanediol	C07182	No ID	122.080	8.52	-1.3E-02	-9.5E-02
C_0034	2-Phenylethylamine	C05332	HMDB12275	122.096	8.05	-3.6E-02	-6.0E-02
C_0035	Nicotinamide	C00153	HMDB01406	123.055	7.61	8.5E-02	9.5E-03
C_0036	Taurine	C00245	HMDB00251	126.022	22.73	8.5E-02	1.0E-02
C_0037	1-Methylhistamine	C05127	HMDB00898	126.102	5.06	-5.7E-02	9.0E-02
C_0039	XC0016	-	-	129.066	9.00	7.6E-02	2.1E-02
C_0040	Pipecolic acid	C00408	HMDB00070,H	130.085	10.60	3.8E-02	5.9E-03
C_0041	*N*-Methylproline	No ID	No ID	130.086	12.87	3.2E-02	-1.6E-02
C_0042	*N*-Acetylputrescine	C02714	HMDB02064	131.117	8.76	-3.1E-02	-8.4E-02
C_0043	Hydroxyproline	C01157	HMDB00725	132.065	12.38	7.5E-02	6.6E-02
C_0044	3-Guanidinopropionic acid	C03065	No ID	132.076	8.26	8.3E-02	5.1E-03
C_0045	Creatine	C00300	HMDB00064	132.410	9.06	-4.4E-02	8.0E-02
C_0046	Ile	C00407,C0641	HMDB00172	132.102	10.51	4.8E-02	1.0E-01
C_0047	Leu	C00123,C0157	HMDB00687	132.102	10.62	6.3E-02	6.0E-02
C_0048	6-Aminohexanoic acid	C02378	HMDB01901	132.102	8.59	-1.9E-02	-1.0E-01
C_0049	Gly-Gly	C02037	HMDB11733	133.059	8.64	8.4E-02	5.0E-03
C_0050	Asn	C00152,C0190	HMDB00168	133.061	10.78	8.4E-02	1.7E-02
C_0051	Ornithine	C00077,C0051	HMDB00214,H	133.097	7.02	8.3E-02	2.7E-02
C_0052	Thiaproline	No ID	No ID	134.027	14.33	-3.7E-02	9.5E-02
C_0053	Asp	C00049,C0040	HMDB00191,H	134.045	11.88	6.7E-02	5.6E-02
C_0054	Adenine	C00147	HMDB00034	136.062	7.84	5.7E-02	3.2E-02
C_0055	Hypoxanthine	C00262	HMDB00157	137.046	11.43	3.1E-02	1.5E-01
C_0057	Trigonelline	C01004	HMDB00875	138.054	10.80	4.5E-02	9.7E-03
C_0058	Tyramine	C00483	HMDB00306	138.091	8.57	-4.9E-02	-3.0E-02
C_0059	Urocanic acid	C00785	HMDB00301	139.049	8.50	-1.7E-02	-3.2E-02
C_0060	1-Methyl-4-imidazoleacetic acid	C05828	HMDB02820	141.066	8.49	6.1E-02	1.0E-01
C_0061	Hexamine	C07176	No ID	141.113	7.56	-1.8E-02	-8.6E-02
C_0062	XC0029	-	-	144.101	12.57	2.4E-02	8.5E-03
C_0063	Stachydrine	C10172	HMDB04827	144.102	11.84	8.1E-02	1.2E-02
C_0064	Acetylcholine	C01996	HMDB00895	146.116	7.84	6.5E-02	2.9E-03
C_0065	γ-Butyrobetaine	C01181	HMDB01161	146.117	8.33	8.5E-02	4.9E-03
C_0066	Spermidine	C00315	HMDB01257	146.165	4.66	7.9E-02	-5.4E-03
C_0067	Gln	C00064,C0030	HMDB00641,H	147.076	11.05	8.5E-02	6.3E-03
C_0068	Lys	C00047,C0073	HMDB00182,H	147.112	7.08	5.7E-02	8.2E-02
C_0069	*threo*-β-Methylaspartic acid	C03618	No ID	148.060	12.73	8.4E-02	-2.9E-05
C_0070	Glu	C00025,C0021	HMDB00148,H	148.060	11.25	5.4E-02	7.4E-02
C_0071	Isoglutamic acid	C05574	No ID	148.060	9.41	7.0E-02	3.6E-03
C_0072	Met	C00073,C0085	HMDB00696	150.058	11.02	6.0E-02	1.1E-01
C_0073	Triethanolamine	C06771	No ID	150.112	8.48	-2.5E-02	8.4E-02
C_0074	Guanine	C00242	HMDB00132	152.057	8.54	-4.4E-02	1.4E-01
C_0075	His	C00135,C0076	HMDB00177	156.076	7.51	8.4E-02	9.0E-03
C_0076	Ala-Ala	C00993	HMDB03459	161.091	9.53	-6.2E-02	5.0E-02
C_0077	N^6-Methyllysine	C02728	HMDB02038	161.128	7.31	8.1E-02	2.7E-02

C_0078	2-Aminoadipic acid	C00956	HMDB00510	162.076	11.28	6.0E-02	6.6E-02
C_0079	Carnitine	C00318,C0048	HMDB00062	162.112	8.76	7.9E-02	4.2E-04
C_0080	5-Hydroxylysine	C16741	HMDB00450	163.107	7.37	-2.6E-02	9.0E-02
C_0081	Phe	C00079,C0205	HMDB00159	166.085	11.40	8.1E-02	4.4E-02
C_0082	Taurocyamine	C01959	HMDB03584	168.042	22.99	8.0E-02	-2.8E-03
C_0083	Tyr-Arg_divalent	No ID	No ID	169.594	7.95	-5.3E-02	2.4E-02
C_0084	Noradrenaline	C00547	HMDB00216	170.082	9.28	-3.4E-02	-3.6E-03
C_0085	1-Methylhistidine	No ID	HMDB00001	170.092	7.73	8.4E-02	4.4E-03
C_0086	3-Methylhistidine	C01152	HMDB00479	170.092	7.71	-6.7E-02	8.7E-02
C_0087	XC0040	-	-	174.087	12.56	8.3E-02	4.1E-04
C_0088	*N*-Acetylornithine	C00437	HMDB03357	175.108	9.80	-4.2E-02	4.2E-02
C_0089	N^5-Ethylglutamine	C01047	No ID	175.107	11.74	8.4E-02	6.4E-03
C_0090	Arg	C00062,C0079	HMDB00517,H	175.119	7.32	8.3E-02	3.3E-02
C_0091	Citrulline	C00327	HMDB00904	176.103	11.37	7.8E-02	3.1E-02
C_0092	Serotonin	C00780	HMDB00259	177.102	9.00	-5.9E-04	-2.1E-02
C_0093	Glucosamine	C00329	HMDB01514	180.087	9.57	4.5E-02	-1.0E-02
C_0094	Tyr	C00082,C0153	HMDB00158	182.081	11.68	7.2E-02	7.4E-02
C_0095	Phosphorylcholine	C00588	HMDB01565	184.073	21.19	8.2E-02	4.6E-02
C_0096	N^8-Acetylspermidine	C01029	HMDB02189	188.176	6.52	-4.4E-02	1.0E-01
C_0097	N^6-Acetyllysine	C02727	HMDB00206	189.121	11.80	4.7E-02	4.7E-03
C_0098	Gly-Leu	No ID	No ID	189.123	10.13	-6.5E-02	2.1E-02
C_0099	*N*-Acetyllysine	C12989	HMDB00446	189.124	10.17	8.5E-02	3.3E-03
C_0100	N^6,N^6,N^6-Trimethyllysine	C03793	HMDB01325	189.158	7.39	-7.0E-02	5.0E-02
C_0101	Homocitrulline	C02427	HMDB00679	190.118	11.53	8.5E-02	-1.2E-03
C_0102	Gly-Asp	No ID	No ID	191.065	10.26	8.2E-02	2.9E-02
C_0103	ADMA	C03626	HMDB01539	203.149	7.88	-3.2E-03	9.4E-02
C_0104	SDMA	No ID	HMDB03334	203.150	8.03	7.8E-02	3.1E-02
C_0105	Spermine	C00750	HMDB01256	203.223	4.61	2.8E-02	-8.8E-02
C_0106	*O*-Acetylcarnitine	C02571	HMDB00201	204.122	9.28	8.2E-02	1.1E-02
C_0107	Trp	C00078,C0052	HMDB00929	205.097	11.34	7.5E-02	5.9E-02
C_0108	Carboxymethyllysine	No ID	No ID	205.120	9.51	7.6E-02	7.7E-03
C_0109	Kynurenine	C00328,C0171	HMDB00684	209.091	10.18	7.6E-02	3.6E-02
C_0110	XC0061	-	-	218.138	9.59	8.1E-02	1.1E-02
C_0111	β-Ala-Lys	C05341	No ID	218.150	7.05	6.0E-02	3.4E-03
C_0112	Cystathionine	C00542,C0229	HMDB00099	223.075	10.20	5.8E-02	9.0E-02
C_0113	Carnosine	C00386	HMDB00033	227.113	6.95	8.4E-02	5.2E-03
C_0114	2'-Deoxycytidine	C00881	HMDB00014	228.097	9.69	-6.5E-02	9.5E-02
C_0115	Butyrylcarnitine	C02862	HMDB02013	232.154	9.86	6.8E-02	2.0E-02
C_0116	XC0071	-	-	234.180	6.39	8.3E-02	-3.6E-03
C_0117	Cystine	C00491,C0142	HMDB00192	241.031	11.26	-4.5E-02	-9.3E-02
C_0118	Homocarnosine	C00884	HMDB00745	241.129	7.04	8.3E-02	-2.2E-02
C_0120	Cytidine	C00475	HMDB00089	244.093	9.96	5.7E-02	1.1E-01
C_0121	Uridine	C00299	HMDB00296	245.076	22.78	-3.2E-02	1.5E-01
C_0122	N^1-Acetylspermine	C02567	HMDB01186	245.233	5.72	-2.7E-02	1.2E-02
C_0123	Malonylcarnitine	No ID	HMDB02095	248.111	10.53	8.2E-02	8.6E-03
C_0124	Pyridoxamine-P	C00647	HMDB01555	249.063	10.81	8.5E-02	1.7E-03
C_0125	γ-Glu-Cys	C00669	HMDB01049	251.069	13.23	7.0E-02	1.6E-02
C_0126	XC0089	-	-	255.097	9.77	-4.8E-02	1.1E-01
C_0127	Dyphylline	C07819	No ID	255.107	22.83	5.7E-02	-8.9E-03
C_0128	Glycerophosphocholine	C00670	HMDB00086	258.109	22.32	7.9E-02	-2.2E-03

C_0129	Thiamine	C00378	HMDB00235	265.110	6.78	8.0E-02	7.9E-04
C_0130	Adenosine	C00212	HMDB00050	268.104	10.15	1.3E-02	3.4E-02
C_0131	Inosine	C00294	HMDB00195	269.087	20.11	-9.9E-03	1.3E-01
C_0132	Glu-Glu	C01425	No ID	277.103	11.17	-3.0E-02	5.5E-02
C_0133	Saccharopine	C00449	HMDB00279	277.139	11.07	7.0E-02	6.5E-03
C_0134	Guanosine	C00387	HMDB00133	284.098	12.89	-6.4E-02	1.0E-01
C_0135	Octanoylcarnitine	C02838	HMDB00791	288.215	10.73	6.4E-02	1.7E-02
C_0136	Ophthalmic acid	No ID	HMDB05765	290.133	13.55	-6.2E-02	8.8E-02
C_0137	Argininosuccinic acid	C03406	HMDB00052	291.129	9.73	8.4E-02	5.4E-03
C_0138	5'-Deoxy-5'-methylthioadenosine	C00170	HMDB01173	298.097	10.43	8.1E-02	4.6E-03
C_0139	Glutathione (GSSG)_divalent	C00127	HMDB03337	307.082	12.52	6.9E-02	7.0E-02
C_0140	Glutathione (GSH)	C00051	HMDB00125	308.090	13.61	7.9E-02	-3.4E-03
C_0141	XC0132	-	-	325.160	8.93	-5.5E-02	1.1E-01
C_0142	NMN	C00455	HMDB00229	335.064	21.22	-6.0E-02	8.8E-02
C_0143	TMP	C01081	HMDB02666	345.077	11.01	6.8E-02	5.6E-02
C_0144	*S*-Lactoylglutathione	C03451	HMDB01066	380.112	14.26	7.4E-02	-1.3E-03
C_0145	*S*-Adenosylhomocysteine	C00021	HMDB00939	385.129	8.97	5.8E-02	7.3E-02
C_0146	*S*-Adenosylmethionine	C00019	HMDB01185	399.143	7.32	8.4E-02	2.7E-02
C_0147	Cysteine glutathione disulfide	C05526	HMDB00656	427.095	11.96	-5.0E-02	-1.8E-02

C indicates the cation mode and A indicates the anion mode.

† Metabolites identified from HMT database based on m/z and migration time
