# Supplementary material for: Altered choline level in atherosclerotic lesions: Upregulation of choline transporter-like protein 1 in human coronary unstable plaque
Source: PLoS One. 2023 Feb 17;18(2):e0281730. doi: 10.1371/journal.pone.0281730 (PMC9937458; doi:10.1371/journal.pone.0281730)
Supplement: S4 Table — (PDF) [file pone.0281730.s004.PDF]

Supplementary table 4. Hierarchical clustering analysis of arterial and cardiac metabolites in rabbits fed a conventional diet

Line	ID	HMT DB [†]			m/z	MT/RT	Standardized Relative Area														
							non-injured artery					injured artery					heart				
		Compound name	KEGG ID	HMDB ID			1	2	3	4	5	6	7	8	9	10	11	12	13	14	15
1	A_0076	6-Phosphogluconic acid	C00345	HMDB01316	275.016	15.61	-0.720	-1.063	1.617	-0.826	0.500	-0.300	0.934	1.977	-0.216	0.307	-0.032	-0.343	-1.263	-1.263	0.692
2	A_0001	Glyoxylic acid	C00048	HMDB00119	72.993	12.45	-0.258	-0.258	-0.258	-0.258	-0.258	-0.258	-0.258	3.615	-0.258	-0.258	-0.258	-0.258	-0.258	-0.258	-0.258
3	A_0003	Pyruvic acid	C00022	HMDB00243	87.008	13.71	-0.356	-0.356	-0.356	-0.356	1.420	-0.356	-0.356	3.214	-0.356	-0.356	-0.356	-0.356	-0.356	-0.356	-0.356
4	C_0032	Cys	C00097,C0073	HMDB00574,H	122.027	11.59	-0.622	-0.622	-0.622	0.518	2.087	-0.622	-0.622	2.066	0.625	0.922	-0.622	-0.622	-0.622	-0.622	-0.622
5	C_0042	*N*-Acetylputrescine	C02714	HMDB02064	131.117	8.76	-0.479	-0.479	-0.479	2.143	2.085	-0.479	-0.479	-0.479	-0.479	1.519	-0.479	-0.479	-0.479	-0.479	-0.479
6	A_0075	2,3-Diphosphoglyceric acid	C01159	HMDB01294	264.951	19.84	-0.033	-0.928	-0.620	0.529	2.223	-0.735	-0.928	0.751	0.130	-0.689	0.204	-0.928	-0.279	-0.624	1.926
7	C_0092	Serotonin	C00780	HMDB00259	177.102	9.00	-0.923	-0.387	-0.923	-0.163	2.949	-0.260	-0.923	-0.923	1.225	0.166	-0.001	-0.100	-0.074	0.006	0.330
8	A_0011	*N*-Acetylglycine	No ID	HMDB00532	116.034	9.94	-0.378	-0.378	-0.378	-0.378	2.587	-0.378	-0.378	-0.378	2.333	-0.378	-0.378	-0.378	-0.378	-0.378	-0.378
9	C_0030	Betaine aldehyde_+H_2O	C00576	HMDB01252	120.102	7.66	-0.258	-0.258	-0.258	-0.258	-0.258	-0.258	-0.258	-0.258	3.615	-0.258	-0.258	-0.258	-0.258	-0.258	-0.258
10	A_0030	Pimelic acid	C02656	HMDB00857	159.065	14.75	-0.377	2.193	-0.377	-0.377	-0.377	-0.377	-0.377	-0.377	2.710	-0.377	-0.377	-0.377	-0.377	-0.377	-0.377
11	A_0018	5-Oxohexanoic acid	C02129	No ID	129.055	9.44	-0.377	2.693	-0.377	-0.377	-0.377	-0.377	-0.377	-0.377	2.213	-0.377	-0.377	-0.377	-0.377	-0.377	-0.377
12	A_0099	Cholic acid	C00695	HMDB00619	407.280	7.01	-0.472	2.190	1.250	-0.472	-0.472	-0.472	-0.472	-0.472	2.228	-0.472	-0.472	-0.472	-0.472	-0.472	-0.472
13	C_0011	Glycerol	C00116	HMDB00131	93.055	22.74	-0.980	-0.368	1.878	0.896	0.491	-0.283	-0.667	-0.794	2.013	0.631	-1.011	-0.146	-0.788	-1.020	0.150
14	C_0034	2-Phenylethylamine	C05332	HMDB12275	122.096	8.05	0.020	-0.962	0.890	0.535	0.630	-0.962	-0.962	0.386	2.539	0.504	-0.962	0.341	-0.962	-0.962	-0.072
15	C_0117	Cystine	C00491,C0142	HMDB00192	241.031	11.26	1.346	-0.681	-0.681	1.141	0.148	-0.681	-0.681	0.048	2.337	1.109	-0.681	-0.681	-0.681	-0.681	-0.681
16	C_0058	Tyramine	C00483	HMDB00306	138.091	8.57	0.630	1.234	-0.752	-0.752	1.236	-0.752	-0.752	0.801	2.034	0.831	-0.752	-0.752	-0.752	-0.752	-0.752
17	A_0027	Adipic acid	C06104	HMDB00448	145.050	16.14	0.089	0.565	-0.651	0.460	1.162	-0.651	-0.651	0.823	2.761	-0.651	-0.651	-0.651	-0.651	-0.651	-0.651
18	C_0088	*N*-Acetylornithine	C00437	HMDB03357	175.108	9.80	-0.628	0.425	-0.628	-0.628	0.867	-0.628	0.914	1.998	2.074	-0.628	-0.628	-0.628	-0.628	-0.628	-0.628
19	C_0147	Cysteine glutathione disulfide	C05526	HMDB00656	427.095	11.96	-0.438	-0.157	-0.539	-0.087	1.045	-0.440	-0.579	0.950	2.522	1.385	-0.732	-0.732	-0.732	-0.732	-0.732
20	A_0033	Quinolinic acid	C03722	HMDB00232	166.014	17.08	-0.445	-0.445	-0.445	-0.445	-0.445	-0.445	-0.445	1.060	2.955	1.328	-0.445	-0.445	-0.445	-0.445	-0.445
21	C_0084	Noradrenaline	C00547	HMDB00216	170.082	9.28	-0.481	-0.481	-0.481	-0.481	-0.481	-0.481	-0.481	1.882	2.165	1.730	-0.481	-0.481	-0.481	-0.481	-0.481
22	A_0017	4-Methyl-2-oxovaleric acid<br>3-Methyl-2-oxovaleric acid	C00233<br>C00671,C034<br>65	HMDB00695<br>HMDB00491	129.054	9.96	-0.569	-0.569	-0.569	-0.569	-0.569	1.250	-0.569	1.792	2.108	1.105	-0.569	-0.569	-0.569	-0.569	-0.569
23	C_0076	Ala-Ala	C00993	HMDB03459	161.091	9.53	-0.937	0.262	-0.937	0.072	0.463	0.676	0.676	1.675	1.582	1.157	-0.937	-0.937	-0.937	-0.937	-0.937
24	C_0083	Tyr-Arg_divalent	No ID	No ID	169.594	7.95	-0.782	-0.782	-0.782	0.957	0.424	0.979	0.071	0.739	2.478	0.605	-0.782	-0.782	-0.782	-0.782	-0.782
25	C_0037	1-Methylhistamine	C05127	HMDB00898	126.102	5.06	-0.766	-0.221	-0.116	-0.457	0.256	1.519	1.147	0.288	2.360	0.298	-0.862	-0.862	-0.862	-0.862	-0.862
26	A_0020	*N*-Acetylalanine	No ID	HMDB00766	130.051	9.20	-0.925	0.065	0.422	-0.101	-0.315	1.050	0.854	0.979	2.396	0.202	-0.925	-0.925	-0.925	-0.925	-0.925
27	C_0098	Gly-Leu	No ID	No ID	189.123	10.13	-0.298	0.096	0.375	-0.086	0.192	0.203	0.133	0.968	2.710	0.670	-0.993	-0.993	-0.993	-0.993	-0.993
28	A_0102	3',5'-ADP	C00054	HMDB00061	426.021	14.32	-0.175	-0.481	0.941	-0.053	0.764	0.754	-0.155	1.099	2.231	0.278	-1.040	-1.040	-1.040	-1.040	-1.040
29	A_0082	Ribulose 1,5-diphosphate	C01182	No ID	308.978	17.01	-0.678	-0.678	1.473	-0.678	1.270	1.176	-0.678	1.701	1.161	-0.678	-0.678	-0.678	-0.678	-0.678	-0.678
30	A_0048	XA0017	-	-	186.113	7.98	-0.846	0.395	1.742	-1.276	0.094	0.154	0.704	1.047	1.880	-0.461	-0.235	-0.255	-0.959	-0.784	-1.200
31	A_0041	Isovalerylalanine	No ID	HMDB00747	172.098	8.26	-1.123	0.664	1.256	0.330	0.821	-0.050	0.739	0.716	1.135	1.130	-1.123	-1.123	-1.123	-1.123	-1.123
32	A_0057	Sebacic acid	C08277	HMDB00792	201.112	12.28	-1.145	1.065	0.936	0.543	1.015	0.568	0.351	0.707	1.312	0.370	-1.145	-1.145	-1.145	-1.145	-1.145
33	A_0043	Suberic acid	C08278	HMDB00893	173.081	13.71	-1.100	1.356	1.319	0.137	0.589	0.498	0.423	0.676	1.364	0.237	-1.100	-1.100	-1.100	-1.100	-1.100
34	A_0049	Azelaic acid	C08261	HMDB00784	187.097	12.92	-1.078	1.863	1.425	0.545	0.610	0.368	0.262	0.545	0.439	0.408	-1.078	-1.078	-1.078	-1.078	-1.078
35	A_0035	Phosphoenolpyruvic acid	C00074	HMDB00263	166.974	23.64	-0.840	1.451	0.782	-0.242	0.747	1.561	0.990	-0.042	0.719	0.316	-1.089	-1.089	-1.089	-1.089	-1.089
36	A_0046	2-Phosphoglyceric acid	C00631	HMDB03391	184.984	21.27	-0.607	0.712	0.804	0.110	1.518	1.206	0.498	0.500	0.870	0.420	-1.206	-1.206	-1.206	-1.206	-1.206
37	C_0100	N^6,N^6,N^6-Trimethyllysine	C03793	HMDB01325	189.158	7.39	-0.092	-0.115	0.119	-0.383	1.766	1.533	0.754	1.031	0.474	0.500	-1.117	-1.117	-1.117	-1.117	-1.117
38	A_0047	3-Phosphoglyceric acid	C00197	HMDB00807	184.985	21.31	-1.180	0.620	0.733	-0.349	1.825	1.264	0.410	0.128	0.772	-0.036	-0.863	-0.830	-0.981	-1.832	0.318
39	C_0132	Glu-Glu	C01425	No ID	277.103	11.17	-0.476	1.633	-0.476	-0.476	2.413	1.669	-0.476	-0.476	-0.476	-0.476	-0.476	-0.476	-0.476	-0.476	-0.476
40	A_0014	Benzoic acid	C00180	HMDB01870	121.029	10.31	-0.375	2.812	-0.375	-0.375	-0.375	2.064	-0.375	-0.375	-0.375	-0.375	-0.375	-0.375	-0.375	-0.375	-0.375
41	A_0058	Mucic acid	C00879,C0180	HMDB00639	209.029	15.16	-0.442	1.707	-0.486	-0.527	-0.473	2.394	1.588	-0.416	-0.398	-0.179	-0.554	-0.554	-0.554	-0.554	-0.554
42	A_0040	Decanoic acid	C01571	HMDB00511	171.138	8.18	-0.475	2.441	-0.475	-0.475	-0.475	1.516	1.743	-0.475	-0.475	-0.475	-0.475	-0.475	-0.475	-0.475	-0.475
43	A_0019	Heptanoic acid	No ID	HMDB00666	129.091	8.90	-0.562	2.225	-0.562	-0.562	-0.562	0.831	1.658	-0.562	1.463	-0.562	-0.562	-0.562	-0.562	-0.562	-0.562
44	A_0025	Octanoic acid	C06423	HMDB00482	143.107	8.59	-0.693	2.231	-0.693	-0.693	0.105	0.695	0.618	-0.693	0.802	-0.693	-0.693	-0.693	-0.693	1.784	-0.693
45	C_0080	5-Hydroxylysine	C16741	HMDB00450	163.107	7.37	-0.377	-0.377	-0.377	-0.377	-0.377	2.721	-0.377	-0.377	2.179	-0.377	-0.377	-0.377	-0.377	-0.377	-0.377
46	A_0052	XA0019	-	-	191.019	8.46	0.100	-0.633	-0.167	-1.367	-0.931	1.967	0.333	1.332	1.214	-0.192	0.875	-1.220	-0.095	-0.079	-1.137
47	C_0096	N^8-Acetylspermidine	C01029	HMDB02189	188.176	6.52	-0.210	-0.685	-0.685	-0.685	-0.685	1.769	1.827	1.475	0.999	0.304	-0.685	-0.685	-0.685	-0.685	-0.685
48	A_0079	*N*-Acetylglucosamine 1-phosphate	C04256	HMDB01367	300.047	9.96	-0.742	-0.124	-0.323	-0.332	-0.420	1.988	1.585	1.001	1.321	0.351	-0.861	-0.861	-0.861	-0.861	-0.861
49	C_0114	2'-Deoxycytidine	C00881	HMDB00014	228.097	9.69	-0.663	0.169	-0.299	-0.550	0.554	1.859	1.008	1.157	1.337	0.444	-1.004	-1.004	-1.004	-1.004	-1.004
50	C_0142	NMN	C00455	HMDB00229	335.064	21.22	-0.575	0.335	-0.161	-0.404	0.040	2.407	0.243	1.431	0.443	0.932	-0.938	-0.938	-0.938	-0.938	-0.938
51	C_0134	Guanosine	C00387	HMDB00133	284.098	12.89	-0.981	1.362	0.485	-0.628	0.464	1.351	1.028	1.423	0.343	0.233	-1.138	-0.755	-0.993	-1.078	-1.116
52	C_0086	3-Methylhistidine	C01152	HMDB00479	170.092	7.71	-0.051	0.560	0.950	-0.676	0.002	1.931	0.880	1.374	0.339	0.081	-1.078	-1.078	-1.078	-1.078	-1.078
53	C_0136	Ophthalmic acid	No ID	HMDB05765	290.133	13.55	0.200	0.619	0.305	-0.829	-0.020	2.259	0.728	1.409	0.024	0.335	-1.006	-1.006	-1.006	-1.006	-1.006
54	C_0015	GABA	C00334	HMDB00112	104.071	7.91	-0.120	0.258	0.550	-0.845	0.217	1.810	1.541	1.353	0.154	0.121	-1.121	-0.556	-1.121	-1.121	-1.121
55	C_0141	XC0132	-	-	325.160	8.93	-0.040	0.501	0.825	-1.253	-0.219	1.744	1.529	1.422	0.054	-0.088	-0.768	-0.953	-0.861	-0.999	-0.893
56	C_0010	β-Ala	C00099	HMDB00056	90.055	7.55	-0.187	0.453	0.707	-1.267	0.420	1.939	1.341	1.301	-0.267	0.088	-0.911	-1.079	-0.824	-0.805	-0.910
57	C_0008	Sarcosine	C00213	HMDB00271	90.055	9.73	0.734	-0.250	1.319	-0.985	0.888	1.906	0.756	0.880	-0.252	-0.068	-0.985	-0.985	-0.985	-0.985	-0.985
58	A_0044	*N*-Acetylaspartic acid	C01042	HMDB00812	174.040	15.37	0.284	0.504	1.585	-1.558	0.121	1.640	0.692	0.836	0.272	-0.281	-0.817	-0.608	-0.882	-0.095	-1.693
59	C_0016	Choline	C00114	HMDB00097	104.107	7.06	-0.534	1.133	0.870	-1.392	0.068	2.003	1.235	0.188	0.391	-0.585	-0.180	0.008	-1.416	-1.009	-0.780
60	A_0077	Xanthosine	C01762	HMDB00299	283.066	7.80	-0.576	0.390	1.102	-0.751	-0.751	2.005	1.630	0.585	0.874	-0.751	-0.751	-0.751	-0.751	-0.751	-0.751
61	C_0121	Uridine	C00299	HMDB00296	245.076	22.78	-1.369	0.211	-0.231	-1.152	-0.538	2.177	1.490	1.123	0.822	-0.114	-0.754	-0.528	-0.670	-0.456	-0.011
62	A_0066	Ribose 5-phosphate	C00117	HMDB01548	229.011	10.92	-1.366	0.290	-0.144	-0.698	-0.329	1.948	1.790	0.004	1.131	-0.578	-1.366	-0.019	-0.597	-0.579	0.513
63	A_0007	Glyceric acid	C00258	HMDB00139,H	105.019	10.88	-0.845	1.162	0.051	-0.198	0.357	2.377	1.578	-0.176	0.066	-0.146	-0.845	-0.845	-0.845	-0.845	-0.845
64	C_0126	XC0089	-	-	255.097	9.77	-0.677	0.151	-0.743	-0.095	0.082	2.973	0.993	0.423	0.307	0.302	-0.743	-0.743	-0.743	-0.743	-0.743
65	A_0080	*N*-Acetylglucosamine 6-phosphate	C00357	HMDB01062	300.048	9.37	-0.817	-0.171	-0.581	-0.616	-0.523	2.467	1.920	0.017	1.049	-0.104	-0.532	-0.403	-0.512	-0.653	-0.539
66	C_0074	Guanine	C00242	HMDB00132	152.057	8.54	-0.878	0.377	-0.441	-0.597	-0.099	2.071	2.172	0.163	0.932	-0.273	-0.997	-0.386	-0.597	-0.710	-0.740
67	A_0078	Sedoheptulose 7-phosphate	C05382	HMDB01068	289.032	9.89	-0.842	0.875	-0.417	-0.631	-0.545	2.073	2.359	-0.243	0.323	-0.523	-0.371	-0.306	-0.406	-0.842	-0.504
68	A_0063	Myristoleic acid	C08322	HMDB02000	225.185	7.62	-0.442	0.621	-0.442	-0.442	-0.442	2.729	1.955	-0.442	-0.442	-0.442	-0.442	-0.442	-0.442	-0.442	-0.442
69	A_0061	*O*-Succinylhomoserine	C01118	No ID	218.069	13.01	-0.378	-0.378	-0.378	-0.378	-0.378	2.628	2.288	-0.378	-0.378	-0.378	-0.378	-0.378	-0.378	-0.378	-0.378
70	A_0056	Erythrose 4-phosphate	C00279,C0360	HMDB01321	198.999	12.26	-0.048	-0.506	1.562	-0.506	-0.506	1.499	2.549	-0.506	-0.506	-0.506	-0.506	-0.506	-0.506	-0.506	-0.506
71	C_0052	Thiaproline	No ID	No ID	134.027	14.33	0.622	-0.341	0.925	-0.625	-0.625	2.224	2.121	-0.370	-0.358	-0.451	-0.625	-0.625	-0.625	-0.625	-0.625
72	C_0004	Gly	C00037	HMDB00123	76.040	8.55	0.651	0.048	0.493	-1.932	0.154	2.773	0.478	0.350	-0.633	-0.581	-0.482	-0.630	-0.253	-0.151	-0.283
73	C_0045	Creatine	C00300	HMDB00064	132.410	9.06	0.231	1.286	1.720	-1.550	0.795	1.464	0.843	-0.074	-0.724	-0.232	-0.622	-0.866	-0.697	-0.822	-0.751
74	A_0024	Ethanolamine phosphate	C00346	HMDB00224	140.011	8.18	-0.717	0.844	1.274	-2.119	0.731	0.533	1.156	1.467	-0.388	0.240	-1.026	-0.754	-0.328	-0.472	-0.439
75	A_0062	Pantothenic acid	C00864	HMDB00210	218.103	7.77	-0.198	0.794	0.357	-2.006	0.291	0.353	1.354	-0.626	-1.437	-0.471	-1.318	0.563	0.344	0.619	1.380
76	C_0103	ADMA	C03626	HMDB01539	203.149	7.88	-0.392	0.515	0.195	-1.710	-0.040	0.860	0.563	0.473	0.084	-0.175	-1.710	1.501	0.751	-1.710	0.795
77	C_0131	Inosine	C00294	HMDB00195	269.087	20.11	-1.102	0.858	0.541	-1.377	0.030	1.345	1.027	0.473	-0.378	-0.598	-1.122	1.927	-0.093	-0.517	-1.014
78	C_0002	Ethanolamine	C00189	HMDB00149	62.060	6.53	-1.845	0.489	1.270	-1.835	-0.834	1.592	0.525	0.067	0.929	-0.745	-0.206	0.620	-0.013	-0.110	0.095
79	C_0073	Triethanolamine	C06771	No ID	150.112	8.48	-1.516	1.351	2.310	-0.689	-0.423	0.121	0.961	-0.814	0.311	0.384	-0.161	0.207	-0.467	-0.282	-1.294
80	C_0018	Diethanolamine	C06772	HMDB04437	106.086	7.87	-1.191	0.102	2.958	-0.389	-0.250	-0.508	-0.120	-0.072	-0.003	0.808	-0.217	-0.362	-0.486	0.920	-1.191
81	C_0059	Urocanic acid	C00785	HMDB00301	139.049	8.50	-0.258	-0.258	-0.258	-0.258	-0.258	-0.258	-0.258	-0.258	-0.258	3.615	-0.258	-0.258	-0.258	-0.258	-0.258
82	C_0122	N^1-Acetylspermine	C02567	HMDB01186	245.233	5.72	-0.427	2.959	-0.427	-0.427	-0.427	-0.427	-0.427	0.496	-0.427	1.675	-0.427	-0.427	-0.427	-0.427	-0.427
83	A_0055	Gluconic acid	C00257	HMDB00625	195.050	8.40	2.310	1.442	-0.117	0.488	0.484	0.303	-0.252	0.892	-0.395	-0.175	-0.996	-0.996	-0.996	-0.996	-0.996
84	A_0125	$NADP^+$	C00006	HMDB00217	742.070	9.75	1.872	-0.039	1.407	-0.461	0.759	0.050	-0.373	1.676	-0.630	0.350	-0.922	-0.922	-0.922	-0.922	-0.922
85	A_0059	Phosphocreatine	C02305	HMDB01511	210.028	12.84	2.984	-0.594	0.627	-0.281	-0.156	-0.588	-0.588	1.350	-0.117	0.243	-0.554	-0.581	-0.594	-0.594	-0.556
86	A_0042	*cis*-Aconitic acid	C00417	HMDB00072	173.008	30.86	2.968	-1.262	0.034	0.207	-0.021	-1.132	-1.262	0.730	-0.147	0.100	-0.043	-0.067	0.182	-0.277	-0.011
87	A_0053	Citric acid	C00158	HMDB00094	191.019	28.49	3.417	-0.794	-0.135	0.047	-0.017	-0.766	-0.790	0.345	-0.360	-0.023	-0.205	-0.170	-0.177	-0.332	-0.041
88	C_0048	6-Aminohexanoic acid	C02378	HMDB01901	132.102	8.59	3.329	-0.346	-0.346	-0.346	-0.346	-0.346	-0.346	-0.346	-0.346	1.168	-0.346	-0.346	-0.346	-0.346	-0.346
89	A_0111	CTP	C00063	HMDB00082	481.978	13.09	3.615	-0.258	-0.258	-0.258	-0.258	-0.258	-0.258	-0.258	-0.258	-0.258	-0.258	-0.258	-0.258	-0.258	-0.258
90	C_0033	2-Amino-2-(hydroxymethyl)-1,3-propanediol	C07182	No ID	122.080	8.52	3.615	-0.258	-0.258	-0.258	-0.258	-0.258	-0.258	-0.258	-0.258	-0.258	-0.258	-0.258	-0.258	-0.258	-0.258
91	C_0061	Hexamine	C07176	No ID	141.113	7.56	3.125	-0.377	1.586	-0.379	-0.379	-0.353	-0.373	-0.340	-0.379	-0.379	-0.379	-0.255	-0.362	-0.379	-0.379
92	A_0097	UDP	C00015	HMDB00295	402.995	12.09	3.356	-0.578	-0.080	-0.520	-0.271	-0.586	-0.573	-0.463	-0.569	-0.433	0.703	-0.289	-0.042	0.081	0.264
93	C_0105	Spermine	C00750	HMDB01256	203.223	4.61	3.040	-1.015	0.007	-0.711	-0.084	-0.524	-0.309	-0.870	-0.912	-0.563	0.490	0.463	0.477	0.004	0.507
94	A_0096	CDP	C00112	HMDB01546	402.010	11.84	2.616	-0.639	-0.011	-0.537	-0.232	-0.720	-0.667	-0.537	-0.720	-0.720	1.530	-0.720	0.156	0.099	1.105
95	A_0124	NAD^+	C00003	HMDB00902	662.104	6.60	1.275	0.661	1.068	-1.209	0.145	-0.378	-0.818	1.039	-1.227	-0.236	0.071	-1.138	1.508	-1.379	0.618
96	A_0119	UDP-glucuronic acid	C00167	HMDB00935	579.026	11.40	0.618	-1.344	1.810	-1.323	0.533	-1.187	-1.312	0.811	-0.779	-0.653	0.672	0.745	0.403	0.511	0.497
97	A_0051	Isocitric acid	C00311	HMDB00193	191.018	31.70	1.413	-1.741	0.385	1.279	-0.188	-1.741	-1.741	0.413	0.493	0.203	0.010	0.686	0.031	0.119	0.379
98	C_0057	Trigonelline	C01004	HMDB00875	138.054	10.80	-0.379	-0.379	-0.379	-0.379	-0.379	-0.379	-0.379	-0.379	-0.379	-0.379	-0.379	2.357	-0.379	2.566	-0.379
99	C_0130	Adenosine	C00212	HMDB00050	268.104	10.15	-0.557	-0.261	-0.542	-0.614	-0.334	-0.081	-0.090	1.213	-0.420	0.621	-0.553	3.116	-0.372	-0.534	-0.593
100	A_0098	Acetyl CoA_divalent	C00024	HMDB01206	403.554	10.51	-0.497	-0.557	-0.394	-0.386	-0.011	-0.557	-0.557	-0.338	-0.468	-0.134	-0.330	2.118	-0.335	-0.273	2.718
101	C_0093	Glucosamine	C00329	HMDB01514	180.087	9.57	-0.383	-0.383	-0.337	-0.383	-0.313	-0.383	-0.383	-0.367	-0.314	-0.335	-0.383	0.779	-0.007	-0.255	3.448
102	A_0069	Glucosamine 6-phosphate	C00352	HMDB01254	258.038	8.80	-0.369	-0.369	-0.369	-0.369	-0.369	-0.369	-0.369	-0.369	-0.369	-0.369	-0.369	0.412	0.065	0.077	3.510
103	C_0041	*N*-Methylproline	No ID	No ID	130.086	12.87	-0.258	-0.258	-0.258	-0.258	-0.258	-0.258	-0.258	-0.258	-0.258	-0.258	-0.258	-0.258	-0.258	-0.258	3.615
104	A_0112	UTP	C00075	HMDB00285	482.961	13.06	1.459	-0.665	-0.284	-0.665	-0.277	-0.406	-0.665	-0.278	-0.487	-0.665	2.431	0.215	-0.665	-0.665	1.620
105	C_0040	Pipecolic acid	C00408	HMDB00070,H	130.085	10.60	0.066	-0.460	-0.460	-0.460	-0.460	0.632	-0.460	-0.460	-0.460	-0.460	3.438	-0.229	-0.129	-0.125	0.024
106	C_0062	XC0029	-	-	144.101	12.57	0.322	-0.386	-0.386	-0.386	-0.386	0.961	-0.386	-0.386	-0.386	-0.386	3.345	-0.386	-0.386	-0.386	-0.386
107	C_0005	Trimethylamine *N*-oxide	C01104	HMDB00925	76.076	6.79	0.178	-0.611	-0.045	-0.611	-0.611	0.904	-0.345	-0.611	-0.611	-0.611	3.170	-0.296	0.225	0.484	-0.611

108	A_0093	PRPP	C00119	HMDB00280	388.943	16.86	-0.258	-0.258	-0.258	-0.258	-0.258	-0.258	-0.258	-0.258	-0.258	-0.258	3.615	-0.258	-0.258	-0.258	-0.258
109	A_0064	XA0027	-	-	227.200	7.37	-0.357	-0.357	-0.357	-0.357	-0.357	-0.357	-0.357	-0.357	-0.357	-0.357	3.210	-0.357	1.428	-0.357	-0.357
110	C_0021	Histamine	C00388	HMDB00870	112.086	4.91	-0.686	-0.791	-0.792	-0.624	0.299	-0.636	-0.470	-0.750	-0.280	-0.683	2.831	0.812	1.050	0.243	0.477
111	C_0064	Acetylcholine	C01996	HMDB00895	146.116	7.84	-0.513	-0.513	-0.513	-0.513	-0.513	-0.513	-0.513	-0.513	-0.513	-0.513	2.935	1.012	0.985	0.708	-0.513
112	C_0097	*N*[6]-Acetyllysine	C02727	HMDB00206	189.121	11.80	-0.379	-0.379	-0.379	-0.379	-0.379	-0.379	-0.379	-0.379	-0.379	-0.379	2.455	2.471	-0.379	-0.379	-0.379
113	A_0045	Hippuric acid	C01586	HMDB00714	178.050	8.35	-0.501	-0.501	-0.501	-0.501	-0.501	-0.501	-0.501	-0.501	-0.501	-0.501	2.124	2.264	1.278	-0.317	-0.339
114	A_0105	XA0065	-	-	445.053	7.01	-0.425	0.973	0.939	-2.291	0.201	-0.343	0.159	0.026	-1.418	-1.127	0.113	0.758	0.920	1.347	0.167
115	C_0078	2-Aminoadipic acid	C00956	HMDB00510	162.076	11.28	-0.754	-0.549	1.035	-1.750	-0.909	0.337	-0.625	0.198	-0.718	-0.935	0.403	1.112	1.691	1.405	0.058
116	C_0112	Cystathionine	C00542,C0229	HMDB00099	223.075	10.20	-1.074	-0.396	0.501	-1.842	-0.343	0.759	-0.120	0.400	-0.602	-1.842	1.207	0.522	0.830	0.998	1.002
117	C_0053	Asp	C00049,C0040	HMDB00191,H	134.045	11.88	-0.687	-0.528	-0.276	-1.676	-0.532	0.468	-0.386	0.304	-0.716	-1.326	1.868	0.719	0.204	1.368	1.196
118	C_0019	Hypotaurine	C00519	HMDB00965	110.027	18.68	-0.220	-0.547	-0.344	-1.463	-0.574	0.626	-0.042	-0.639	-1.142	-0.965	1.693	0.288	0.152	1.507	1.669
119	C_0070	Glu	C00025,C0021	HMDB00148,H	148.060	11.25	-0.410	-0.066	-0.144	-1.873	-0.706	0.796	0.384	-0.191	-0.837	-1.177	1.371	0.603	-0.200	0.327	2.123
120	C_0007	Putrescine	C00134	HMDB01414	89.108	4.84	-1.057	-0.003	-0.218	-1.781	-0.423	0.012	0.198	-0.344	-0.949	-0.779	1.414	0.349	0.599	0.821	2.160
121	C_0143	TMP	C01081	HMDB02666	345.077	11.01	-1.096	-0.630	-0.781	-1.198	-0.952	-0.049	0.740	-0.268	-0.821	-0.426	0.953	0.808	0.359	1.113	2.246
122	C_0071	Isoglutamic acid	C05574	No ID	148.060	9.41	-0.603	-0.588	-0.556	-0.759	-0.589	-0.377	-0.279	-0.607	-0.623	-0.617	1.268	0.774	0.121	0.582	2.853
123	A_0037	Dihydroxyacetone phosphate	C00111	HMDB01473	168.990	13.25	-0.748	-0.660	-0.567	-0.583	-0.550	-0.486	-0.318	-0.593	-0.435	-0.615	0.733	0.460	0.384	0.992	2.984
124	A_0090	IMP	C00130	HMDB00175	347.038	9.77	-0.386	-0.457	-0.316	-0.457	-0.435	-0.460	-0.460	-0.460	-0.460	-0.455	0.884	-0.103	-0.281	0.539	3.306
125	A_0070	Glucose 1-phosphate	C00103	HMDB01586	259.021	10.44	-0.311	-0.714	-0.067	-0.620	-0.434	-0.648	-0.573	-0.330	-0.577	-0.397	0.795	-0.427	0.040	1.246	3.016
126	A_0071	Glucose 6-phosphate	C00668,C0117	HMDB01401	259.021	10.12	-0.607	-0.595	-0.373	-0.566	-0.511	-0.588	-0.526	-0.533	-0.556	-0.563	0.878	-0.096	0.296	1.564	2.777
127	A_0073	Fructose 6-phosphate	C05345,C0008	HMDB00124	259.021	10.15	-0.610	-0.590	-0.468	-0.560	-0.610	-0.580	-0.532	-0.610	-0.550	-0.610	0.994	-0.069	0.462	1.944	2.392
128	A_0087	Fructose 1,6-diphosphate	C00354	HMDB01058	338.988	15.39	-0.563	-0.624	-0.554	-0.603	-0.523	-0.609	-0.613	-0.554	-0.585	-0.590	1.841	-0.195	0.532	1.451	2.189
129	A_0116	GTP	C00044	HMDB01273	521.983	11.83	-0.089	-0.712	-0.563	-0.693	-0.558	-0.651	-0.751	-0.473	-0.697	-0.694	1.769	0.607	0.640	0.386	2.478
130	A_0115	ATP	C00002	HMDB00538	505.988	12.19	-0.586	-0.523	-0.438	-0.606	-0.364	-0.390	-0.541	-0.416	-0.536	-0.601	2.728	-0.121	0.326	0.105	1.962
131	A_0072	*myo*-Inositol 2-phosphate	No ID	No ID	259.021	10.86	-0.753	-0.129	-0.753	-0.753	-0.244	0.224	-0.165	-0.753	-0.194	-0.156	2.601	-0.753	-0.753	0.911	1.670
132	A_0084	CMP	C00055	HMDB00095	322.041	9.83	-0.488	-0.658	0.256	-0.800	-0.424	-0.831	-0.799	-0.702	-0.777	-0.730	2.336	0.290	0.733	1.435	1.160
133	A_0085	UMP	C00105	HMDB00288	323.027	10.06	-0.368	-0.675	-0.280	-0.719	-0.622	-0.743	-0.745	-0.701	-0.714	-0.692	2.165	0.451	0.982	1.801	0.861
134	C_0079	Carnitine	C00318,C0048	HMDB00062	162.112	8.76	-0.609	-0.646	-0.574	-0.672	-0.661	-0.628	-0.625	-0.599	-0.633	-0.654	2.459	0.785	1.336	1.078	0.642
135	A_0103	ADP	C00008	HMDB01341	426.021	11.23	-0.432	-0.695	-0.440	-0.661	-0.538	-0.751	-0.752	-0.651	-0.720	-0.663	2.383	0.470	1.279	1.066	1.108
136	A_0092	CoA_divalent	C00010	HMDB01423	382.549	10.95	-0.618	-0.694	-0.525	-0.664	-0.637	-0.681	-0.686	-0.571	-0.680	-0.660	2.144	0.470	1.649	1.109	1.043
137	A_0023	Threonic acid	C01620	HMDB00943	135.031	9.19	-0.576	-0.576	-0.576	-0.576	-0.576	-0.576	-0.576	-0.576	-0.576	-0.576	2.030	-0.576	1.599	1.285	1.422
138	C_0144	*S*-Lactoylglutathione	C03451	HMDB01066	380.112	14.26	-0.588	-0.588	-0.588	-0.588	-0.588	-0.588	-0.588	-0.588	-0.588	-0.588	2.097	-0.039	1.574	1.877	0.374
139	A_0060	3-Indoxylsulfuric acid	No ID	HMDB00682	212.003	9.71	-0.930	-0.559	-0.792	-1.144	-0.547	-0.094	-0.199	-0.652	-0.335	-0.590	1.783	0.487	1.356	2.094	0.121
140	A_0034	XA0012	-	-	166.018	9.63	-0.874	-0.523	-0.612	-0.773	-0.711	0.060	-0.530	-0.567	-0.602	-0.675	2.342	0.281	1.848	0.976	0.359
141	C_0043	Hydroxyproline	C01157	HMDB00725	132.065	12.38	-1.151	-0.550	-0.578	-1.546	-0.658	0.378	-0.100	-0.448	-0.597	-0.699	1.961	0.948	1.050	0.680	1.308
142	C_0104	SDMA	No ID	HMDB03334	203.150	8.03	-0.940	-0.255	-0.940	-0.940	-0.940	0.254	-0.940	-0.299	-0.940	-0.365	1.400	0.943	0.906	1.504	1.554
143	C_0109	Kynurenine	C00328,C0171	HMDB00684	209.091	10.18	-1.112	-0.283	-1.112	-0.951	-0.532	-0.446	-0.310	-0.572	-0.312	-0.616	0.472	1.545	1.000	2.027	1.201
144	C_0028	Thr	C00188,C0082	HMDB00167	120.065	10.82	-1.026	-0.217	-0.402	-1.612	-0.723	-0.166	0.039	-0.261	-0.666	-0.934	0.384	1.817	1.356	1.250	1.163
145	C_0091	Citrulline	C00327	HMDB00904	176.103	11.37	-1.061	-0.800	-0.224	-1.358	-0.525	-0.386	-0.645	-0.180	-0.404	-0.605	1.107	0.469	2.008	1.117	1.488
146	A_0081	*N*-Acetylneuraminic acid	C00270	HMDB00230	308.098	7.26	-1.094	-0.728	-0.737	-0.965	-0.896	-0.408	-0.611	-0.652	0.494	-0.753	1.037	1.387	1.511	1.116	1.301
147	C_0023	Creatinine	C00791	HMDB00562	114.066	7.51	-0.787	-0.619	-0.708	-0.965	-0.523	-0.377	-0.678	-0.716	-0.693	-0.532	1.194	1.419	1.238	0.793	1.952
148	A_0016	5-Oxoproline	C01879	HMDB00267	128.035	9.73	-0.854	-0.543	-0.584	-0.864	-0.740	-0.372	-0.423	-0.632	-0.795	-0.834	1.512	1.372	1.292	0.774	1.691
149	C_0077	*N*[6]-Methyllysine	C02728	HMDB02038	161.128	7.31	-1.017	-0.654	-0.645	-1.142	-0.440	-0.169	-0.673	-0.591	-0.702	-0.459	1.822	1.266	1.100	0.765	1.540
150	A_0086	cAMP	C00575	HMDB00058	328.044	7.43	-0.672	-0.672	-0.672	-0.672	-0.672	-0.672	-0.672	-0.672	-0.672	-0.672	1.912	1.260	1.287	0.999	1.261
151	A_0107	FMN	C00061	HMDB01520	455.098	8.25	-0.674	-0.674	-0.674	-0.674	-0.674	-0.674	-0.674	-0.674	-0.674	-0.674	1.826	1.183	1.035	1.465	1.229
152	C_0101	Homocitrulline	C02427	HMDB00679	190.118	11.53	-0.678	-0.678	-0.678	-0.678	-0.678	-0.678	-0.678	-0.678	-0.678	-0.678	1.604	1.002	1.298	1.339	1.534
153	C_0116	XC0071	-	-	234.180	6.39	-0.670	-0.670	-0.670	-0.670	-0.670	-0.670	-0.670	-0.670	-0.670	-0.670	1.518	0.897	1.004	1.531	1.748
154	C_0069	*threo*-β-Methylaspartic acid	C03618	No ID	148.060	12.73	-0.678	-0.678	-0.678	-0.678	-0.678	-0.678	-0.678	-0.678	-0.678	-0.678	1.231	1.223	1.129	1.702	1.492
155	A_0022	Malic acid	C00149,C0049	HMDB00156,H	133.014	23.19	-0.724	-0.683	-0.687	-0.729	-0.637	-0.571	-0.685	-0.675	-0.680	-0.694	1.111	1.234	1.173	1.587	1.659
156	A_0008	Fumaric acid	C00122	HMDB00134	115.003	27.82	-0.724	-0.668	-0.679	-0.703	-0.665	-0.607	-0.671	-0.677	-0.671	-0.689	1.067	1.165	1.290	1.470	1.763
157	A_0039	Glycerol 3-phosphate	C00093	HMDB00126	171.006	12.64	-0.715	-0.626	-0.633	-0.760	-0.646	-0.556	-0.735	-0.691	-0.693	-0.711	1.026	1.485	1.342	1.252	1.661
158	A_0015	Isethionic acid	C05123	HMDB03903	124.991	12.00	-0.795	-0.663	-0.509	-0.721	-0.602	-0.630	-0.663	-0.597	-0.795	-0.795	1.277	1.221	1.312	1.247	1.712
159	A_0094	FAD_divalent	C00016	HMDB01248	391.571	7.98	-0.794	-0.681	-0.633	-0.753	-0.675	-0.597	-0.686	-0.575	-0.685	-0.714	1.242	1.183	1.412	1.330	1.626
160	A_0068	Biotin	C00120	HMDB00030	243.081	7.55	-0.682	-0.682	-0.682	-0.682	-0.682	-0.682	-0.682	-0.682	-0.682	-0.682	1.394	1.284	1.450	1.264	1.432
161	A_0123	CMP-*N*-acetylneuraminate	C00128	HMDB01176	613.140	8.08	-0.679	-0.679	-0.679	-0.679	-0.679	-0.679	-0.679	-0.679	-0.679	-0.679	1.442	1.309	1.466	1.010	1.558
162	C_0067	Gln	C00064,C0030	HMDB00641,H	147.076	11.05	-0.675	-0.674	-0.626	-0.776	-0.689	-0.589	-0.656	-0.663	-0.719	-0.737	1.499	1.414	1.305	1.126	1.460
163	A_0100	ThPP	C00068	HMDB01372	423.029	7.87	-0.680	-0.680	-0.680	-0.680	-0.680	-0.680	-0.680	-0.680	-0.680	-0.680	1.512	1.457	1.188	1.177	1.470
164	C_0035	Nicotinamide	C00153	HMDB01406	123.055	7.61	-0.774	-0.683	-0.673	-0.750	-0.703	-0.609	-0.557	-0.699	-0.637	-0.731	1.477	1.319	1.289	1.373	1.359
165	C_0124	Pyridoxamine-P	C00647	HMDB01555	249.063	10.81	-0.682	-0.682	-0.682	-0.682	-0.682	-0.682	-0.682	-0.682	-0.682	-0.682	1.358	1.455	1.202	1.461	1.345
166	C_0036	Taurine	C00245	HMDB00251	126.022	22.73	-0.604	-0.669	-0.526	-0.940	-0.698	-0.513	-0.575	-0.655	-0.807	-0.776	1.383	1.323	1.094	1.552	1.413
167	A_0120	ADP-glucose<br>GDP-fucose	C00498<br>C00325	HMDB06557<br>HMDB01095	588.075	8.46	-0.469	-0.803	-0.243	-0.954	-0.587	-0.814	-0.811	-0.473	-0.783	-0.765	1.265	1.362	1.447	1.121	1.507
168	A_0091	GMP	C00144	HMDB01397	362.049	9.37	-0.490	-0.717	-0.332	-0.773	-0.600	-0.784	-0.787	-0.759	-0.750	-0.725	1.270	0.948	1.422	1.453	1.623
169	C_0113	Carnosine	C00386	HMDB00033	227.113	6.95	-0.671	-0.703	-0.697	-0.699	-0.675	-0.621	-0.665	-0.640	-0.682	-0.666	0.900	1.156	1.790	1.394	1.479
170	C_0099	*N*-Acetyllysine	C12989	HMDB00446	189.124	10.17	-0.680	-0.680	-0.680	-0.680	-0.680	-0.680	-0.680	-0.680	-0.680	-0.680	1.197	1.287	1.550	1.555	1.211
171	C_0006	Morpholine	C14452	HMDB31581	88.075	6.83	-0.678	-0.678	-0.678	-0.678	-0.678	-0.678	-0.678	-0.678	-0.678	-0.678	1.144	1.521	1.416	1.608	1.092
172	C_0137	Argininosuccinic acid	C03406	HMDB00052	291.129	9.73	-0.672	-0.672	-0.672	-0.672	-0.672	-0.672	-0.672	-0.672	-0.672	-0.672	0.778	1.575	1.556	1.541	1.267
173	C_0027	Betaine	C00719	HMDB00043	118.086	11.59	-0.682	-0.689	-0.677	-0.741	-0.645	-0.626	-0.672	-0.684	-0.687	-0.661	1.044	1.645	1.583	1.291	1.202
174	C_0049	Gly-Gly	C02037	HMDB11733	133.059	8.64	-0.671	-0.671	-0.671	-0.671	-0.671	-0.671	-0.671	-0.671	-0.671	-0.671	0.942	1.884	1.221	1.410	1.256
175	C_0075	His	C00135,C0076	HMDB00177	156.076	7.51	-0.720	-0.661	-0.670	-0.767	-0.680	-0.592	-0.634	-0.655	-0.652	-0.688	1.351	1.737	0.948	1.588	1.095
176	C_0050	Asn	C00152,C0190	HMDB00168	133.061	10.78	-0.778	-0.615	-0.640	-0.881	-0.703	-0.487	-0.587	-0.662	-0.642	-0.768	1.326	1.558	1.166	1.575	1.137
177	C_0065	γ-Butyrobetaine	C01181	HMDB01161	146.117	8.33	-0.675	-0.673	-0.663	-0.718	-0.663	-0.644	-0.671	-0.696	-0.695	-0.680	1.350	1.726	1.150	1.372	1.181
178	A_0032	Terephthalic acid	C06337	HMDB02428	165.018	17.58	-0.721	-0.682	-0.544	-0.716	-0.724	-0.722	-0.605	-0.739	-0.613	-0.695	1.451	1.738	1.270	1.176	1.126
179	C_0044	3-Guanidinopropionic acid	C03065	No ID	132.076	8.26	-0.665	-0.665	-0.665	-0.665	-0.665	-0.665	-0.665	-0.665	-0.665	-0.665	1.635	1.721	1.225	1.417	0.654
180	A_0117	ADP-ribose	C00301	HMDB01178	558.064	8.67	-0.748	-0.705	-0.401	-0.734	-0.650	-0.733	-0.634	-0.618	-0.679	-0.779	1.191	1.290	1.314	1.922	0.963
181	C_0146	*S*-Adenosylmethionine	C00019	HMDB01185	399.143	7.32	-0.875	-0.529	-0.395	-1.198	-0.556	-0.483	-0.512	-0.537	-0.837	-0.755	1.290	1.381	1.389	1.507	1.110
182	C_0095	Phosphorylcholine	C00588	HMDB01565	184.073	21.19	-0.927	-0.511	-0.586	-1.419	-0.700	-0.164	-0.359	-0.354	-0.696	-0.825	1.323	1.447	1.295	1.200	1.275
183	A_0067	XA0033	-	-	242.079	7.75	-0.998	-0.444	-0.540	-1.353	-0.692	-0.263	-0.405	-0.435	-0.652	-0.803	1.275	1.477	1.281	1.211	1.340
184	C_0090	Arg	C00062,C0079	HMDB00517,H	175.119	7.32	-0.896	-0.579	-0.570	-1.136	-0.764	-0.338	-0.443	-0.474	-0.663	-0.755	1.185	1.805	1.200	1.031	1.397
185	C_0051	Ornithine	C00077,C0051	HMDB00214,H	133.097	7.02	-0.899	-0.569	-0.612	-0.971	-0.770	-0.507	-0.427	-0.576	-0.639	-0.715	1.218	1.823	1.310	1.271	1.063
186	C_0001	Urea	C00086	HMDB00294	61.040	21.79	-0.711	-0.507	-0.698	-0.923	-0.603	-0.452	-0.640	-0.807	-0.724	-0.657	1.352	1.630	1.573	1.016	1.150
187	C_0085	1-Methylhistidine	No ID	HMDB00001	170.092	7.73	-0.670	-0.670	-0.670	-0.670	-0.670	-0.670	-0.670	-0.670	-0.670	-0.670	1.693	1.381	1.693	0.967	0.968
188	A_0028	2-Hydroxyglutaric acid	C02630,C0108	HMDB00606,H	147.029	16.95	-0.667	-0.667	-0.667	-0.667	-0.667	-0.667	-0.667	-0.667	-0.667	-0.667	1.692	1.123	1.840	1.049	0.969
189	C_0138	5'-Deoxy-5'-<br>methylthioadenosine	C00170	HMDB01173	298.097	10.43	-0.648	-0.648	-0.648	-0.648	-0.648	-0.648	-0.648	-0.648	-0.648	-0.648	1.785	1.008	1.709	1.588	0.393
190	C_0110	XC0061	-	-	218.138	9.59	-0.657	-0.657	-0.657	-0.657	-0.657	-0.596	-0.657	-0.591	-0.657	-0.657	1.370	1.442	1.938	1.437	0.257
191	A_0004	Lactic acid	C00186,C0025	HMDB00190,H	89.024	11.18	-0.771	-0.734	-0.660	-0.760	-0.604	-0.533	-0.654	-0.587	-0.647	-0.667	1.274	1.044	1.955	1.551	0.792
192	A_0089	AMP	C00020	HMDB00045	346.055	9.50	-0.602	-0.657	-0.660	-0.687	-0.674	-0.683	-0.679	-0.691	-0.692	-0.682	1.335	1.181	1.724	1.625	0.841
193	C_0118	Homocarnosine	C00884	HMDB00745	241.129	7.04	-0.208	-0.814	-0.705	-0.690	-0.622	-0.812	-0.869	-0.806	-0.835	-0.268	0.980	1.072	1.534	1.476	1.569
194	C_0031	Anserine_divalent	C01262	HMDB00194	121.068	7.04	-0.208	-0.815	-0.720	-0.705	-0.604	-0.796	-0.884	-0.813	-0.839	-0.254	1.045	1.125	1.312	1.536	1.621
195	C_0025	Guanidoacetic acid	C00581	HMDB00128	118.061	8.48	-0.764	-0.764	-0.764	-0.764	-0.457	-0.597	-0.764	-0.655	-0.538	-0.452	0.724	1.527	1.644	0.767	1.856
196	C_0089	*N*[5]-Ethylglutamine	C01047	No ID	175.107	11.74	-0.669	-0.669	-0.669	-0.669	-0.669	-0.669	-0.669	-0.669	-0.669	-0.669	1.018	1.868	1.570	0.961	1.271
197	C_0123	Malonylcarnitine	No ID	HMDB02095	248.111	10.53	-0.653	-0.653	-0.653	-0.653	-0.653	-0.653	-0.653	-0.653	-0.653	-0.653	0.701	1.723	1.998	0.896	1.216
198	C_0106	*O*-Acetylcarnitine	C02571	HMDB00201	204.122	9.28	-0.641	-0.640	-0.623	-0.720	-0.644	-0.607	-0.650	-0.676	-0.659	-0.654	0.517	1.550	2.052	1.197	1.198
199	C_0063	Stachydrine	C10172	HMDB04827	144.102	11.84	-0.698	-0.683	-0.575	-0.729	-0.637	-0.627	-0.667	-0.580	-0.614	-0.665	0.521	1.474	2.085	1.450	0.945
200	C_0009	Ala	C00041,C0013	HMDB00161,H	90.055	9.28	-0.659	-0.599	-0.604	-0.758	-0.689	-0.544	-0.588	-0.614	-0.676	-0.716	0.785	1.503	1.947	1.693	0.519
201	A_0113	CDP-choline	C00307	HMDB01413	487.099	6.83	-0.918	-0.422	-0.719	-1.056	-0.802	-0.227	-0.341	-0.383	-0.743	-0.742	1.434	1.348	1.091	1.998	0.483
202	C_0102	Gly-Asp	No ID	No ID	191.065	10.26	-0.715	-0.440	-0.177	-1.210	-0.663	-0.364	-0.311	-0.606	-1.210	-0.803	1.169	1.119	1.374	1.667	1.169
203	C_0140	Glutathione (GSH)	C00051	HMDB00125	308.090	13.61	-0.695	-0.932	0.016	-0.858	-0.253	-0.806	-0.743	-0.386	-0.868	-0.797	0.429	1.224	1.226	1.743	1.698
204	C_0066	Spermidine	C00315	HMDB01257	146.165	4.66	-0.142	-0.936	0.057	-1.228	-0.285	-0.710	-0.500	-0.926	-0.935	-0.582	0.625	1.675	1.095	0.951	1.841
205	C_0129	Thiamine	C00378	HMDB00235	265.110	6.78	-0.645	-0.645	-0.645	-0.645	-0.645	-0.645	-0.645	-0.645	-0.645	-0.645	0.398	1.372	1.243	1.317	2.124
206	C_0082	Taurocyamine	C01959	HMDB03584	168.042	22.99	-0.642	-0.642	-0.642	-0.642	-0.642	-0.642	-0.642	-0.642	-0.642	-0.642	0.804	1.422	0.735	1.143	2.315
207	A_0108	Adenylosuccinic acid	C03794	HMDB00536	462.066	14.16	-0.621	-0.624	-0.624	-0.624	-0.616	-0.624	-0.624	-0.624	-0.624	-0.618	0.690	1.783	0.517	0.918	2.315
208	C_0128	Glycerophosphocholine	C00670	HMDB00086	258.109	22.32	-0.563	-0.659	-0.612	-0.657	-0.641	-0.658	-0.640	-0.624	-0.649	-0.651	1.257	1.999	0.596	0.582	1.920
209	C_0087	XC0040	-	-	174.087	12.56	-0.663	-0.663	-0.663	-0.663	-0.663	-0.663	-0.663	-0.663	-0.663	-0.663	1.254	1.821	0.793	1.020	1.745
210	C_0003	XC0001	-	-	72.081	6.56	-0.411	-0.193	-0.894	-0.894	-0.121	-0.894	-0.894	-0.894	-0.894	-0.271	1.809	0.944	0.721	1.287	1.602
211	A_0118	UDP-glucose<br>UDP-galactose	C00029<br>C00052	HMDB00286<br>HMDB00302	565.048	8.82	-0.518	-0.385	0.238	-1.317	-0.415	-0.905	-0.738	-0.296	-0.920	-0.813	1.517	0.985	0.756	0.748	2.063
212	A_0122	UDP-*N*-acetylglucosamine	C00043	HMDB00290	606.075	8.62	-0.153	-0.392	0.395	-1.251	-0.605	-1.093	-1.102	-0.165	-0.901	-0.907	1.196	1.175	1.131	1.279	1.392
213	A_0121	GDP-galactose<br>GDP-mannose	C02280<br>C00096	No ID<br>HMDB01163	604.070	8.43	-0.385	-0.724	0.647	-1.248	-0.033	-0.956	-1.193	0.095	-1.079	-0.761	0.639	1.277	1.613	1.502	0.606
214	A_0104	GDP	C00035	HMDB01201	442.016	10.99	1.312	-0.943	-0.490	-0.796	-0.524	-0.972	-0.977	-0.659	-0.894	-0.709	1.113	1.100	1.511	0.886	1.042
215	A_0012	Succinic acid	C00042	HMDB00254	117.019	22.73	-0.611	-0.591	-0.593	-0.607	-0.605	-0.570	-0.593	-0.576	-0.592	-0.606	1.218	1.695	2.408	0.552	0.071
216	C_0039	XC0016	-	-	129.066	9.00	-0.690	-0.698	-0.087	-0.904	-0.682	-0.526	-0.593	-0.342	-0.734	-0.661	1.122	1.214	2.613	0.486	0.482
217	C_0024	Pro	C00148,C0076	HMDB00162,H	116.070	11.12	-1.001	-0.820	-0.525	-1.216	-0.758	-0.072	-0.290	-0.217	-0.508	-0.658	1.594	1.289	2.023	0.896	0.262
218	C_0017	Ser	C00065,C0071	HMDB00187,H	106.050	10.27	-0.725	-0.546	-0.505	-1.312	-0.751	0.212	-0.331	-0.504	-0.653	-0.959	1.608	1.041	1.734	1.468	0.223
219	C_0081	Phe	C00079,C0205	HMDB00159	166.085	11.40	-0.972	-0.651	-0.580	-1.184	-0.769	-0.306	-0.313	-0.406	-0.495	-0.764	0.781	1.606	1.895	1.136	1.021

220	C_0107	Trp	C00078,C0052	HMDB00929	205.097	11.34	-1.116	-0.536	-0.985	-1.178	-0.695	0.013	-0.297	-0.232	-0.429	-0.555	0.683	1.675	2.139	0.723	0.789
221	C_0094	Tyr	C00082,C0153	HMDB00158	182.081	11.68	-1.157	-0.546	-0.599	-1.352	-0.904	0.136	-0.068	-0.218	-0.202	-0.848	0.876	1.286	2.266	0.872	0.457
222	C_0026	Val	C00183,C0641	HMDB00883	118.086	10.31	-0.989	-0.667	-0.308	-1.478	-0.858	0.094	-0.087	0.019	-0.211	-0.819	0.293	1.522	2.357	0.907	0.225
223	C_0047	Leu	C00123,C0157	HMDB00687	132.102	10.62	-0.918	-0.538	-0.370	-1.096	-0.704	-0.075	-0.267	-0.178	-0.158	-0.707	-0.190	1.794	2.471	1.016	-0.080
224	C_0068	Lys	C00047,C0073	HMDB00182,H	147.112	7.08	-0.976	-0.557	-0.093	-1.621	-0.757	0.286	-0.096	0.385	-0.438	-0.626	-0.343	1.718	2.182	0.935	0.001
225	C_0046	Ile	C00407,C0641	HMDB00172	132.102	10.51	-1.307	-0.317	-0.146	-1.358	-0.624	0.409	0.228	0.019	0.039	-0.791	-0.437	1.533	2.437	0.688	-0.374
226	C_0072	Met	C00073,C0085	HMDB00696	150.058	11.02	-1.777	-0.417	-0.776	-1.505	-0.714	0.485	0.304	-0.079	0.366	-0.733	0.481	1.315	1.902	0.728	0.420
227	C_0014	2-Aminoisobutyric acid	C03665	HMDB01906	104.071	9.92	-0.870	-0.481	0.119	-1.986	-0.785	0.609	0.519	0.235	-0.972	-1.014	1.239	1.027	1.724	0.357	0.281
228	C_0060	1-Methyl-4-imidazoleacetic acid	C05828	HMDB02820	141.066	8.49	-0.955	-0.818	-0.075	-1.605	-0.907	0.998	0.633	-0.398	-0.730	-0.919	0.810	1.437	1.746	0.327	0.455
229	A_0006	2-Hydroxybutyric acid	C05984	HMDB00008	103.040	10.12	-0.531	-0.236	-0.531	-0.531	-0.531	0.209	-0.189	-0.531	-0.231	-0.111	-0.531	2.463	2.341	-0.531	-0.531
230	A_0021	6-Hydroxyhexanoic acid	C06103	No ID	131.071	8.55	-0.375	-0.375	-0.375	-0.375	-0.375	-0.375	-0.375	-0.375	-0.375	-0.375	-0.375	2.806	2.073	-0.375	-0.375
231	C_0054	Adenine	C00147	HMDB00034	136.062	7.84	-0.636	-0.475	-0.392	-0.658	-0.459	-0.415	-0.290	-0.243	-0.548	-0.456	-0.054	3.114	1.280	0.552	-0.322
232	C_0135	Octanoylcarnitine	C02838	HMDB00791	288.215	10.73	-0.512	-0.512	-0.512	-0.512	-0.512	-0.512	-0.512	-0.512	-0.512	-0.512	0.094	3.067	1.331	0.309	0.318
233	C_0125	γ-Glu-Cys	C00669	HMDB01049	251.069	13.23	-0.561	-0.561	-0.561	-0.561	-0.561	-0.561	-0.561	-0.561	-0.561	-0.561	0.168	2.798	1.378	1.019	0.245
234	C_0115	Butyrylcarnitine	C02862	HMDB02013	232.154	9.86	-0.548	-0.528	-0.525	-0.548	-0.541	-0.509	-0.537	-0.548	-0.532	-0.548	0.306	2.565	2.085	0.293	0.114
235	A_0005	3-Hydroxybutyric acid	C01089,C0319	HMDB00011,H	103.040	9.79	-0.552	-0.673	-0.657	-0.695	-0.524	-0.232	-0.625	-0.687	-0.609	-0.567	0.277	2.280	2.100	0.786	0.377
236	A_0054	Phenaceturic acid	C05598	HMDB00821	192.066	8.12	-0.561	-0.561	-0.561	-0.561	-0.561	-0.561	-0.561	-0.561	-0.561	-0.561	0.689	1.961	1.888	1.630	-0.561
237	C_0108	Carboxymethyllysine	No ID	No ID	205.120	9.51	-0.613	-0.613	-0.613	-0.613	-0.613	-0.613	-0.613	-0.613	-0.613	-0.613	1.025	2.659	0.603	1.075	0.772
238	A_0050	10-Hydroxydecanoic acid	C02774	No ID	187.133	7.69	-0.620	-0.620	-0.620	-0.620	-0.620	-0.620	-0.620	-0.620	-0.620	-0.620	0.098	2.168	1.038	1.736	1.161
239	A_0031	8-Hydroxyoctanoic acid	No ID	No ID	159.102	8.04	-0.558	-0.558	-0.558	-0.558	-0.558	-0.558	-0.558	-0.558	-0.558	-0.558	-0.558	2.440	1.074	1.166	1.454
240	A_0106	Octanoyl CoA_divalent	C01944	HMDB01070	445.602	9.53	-0.456	-0.456	-0.456	-0.456	-0.456	-0.456	-0.456	-0.456	-0.456	-0.456	-0.456	2.565	0.870	-0.456	2.041
241	C_0012	Homoserinelactone	No ID	No ID	102.055	7.28	-0.483	-0.483	-0.483	-0.483	-0.483	-0.483	-0.483	-0.483	-0.483	-0.483	-0.483	1.908	1.866	-0.483	2.020
242	C_0133	Saccharopine	C00449	HMDB00279	277.139	11.07	-0.551	-0.551	-0.551	-0.551	-0.551	-0.551	-0.551	-0.551	-0.551	-0.551	-0.030	1.004	2.451	0.105	1.982
243	A_0088	cGMP	C00942	HMDB01314	344.039	7.51	-0.476	-0.476	-0.476	-0.476	-0.476	-0.476	-0.476	-0.476	-0.476	-0.476	1.396	-0.476	2.267	-0.476	2.048
244	C_0013	*N*,*N*-Dimethylglycine	C01026	HMDB00092	104.072	11.27	-0.475	-0.475	-0.475	-0.475	-0.475	-0.475	-0.475	-0.475	-0.475	-0.475	2.405	-0.475	1.817	-0.475	1.483
245	A_0013	2-Hydroxyvaleric acid	No ID	HMDB01863	117.056	9.19	-0.439	-0.439	-0.439	-0.439	-0.439	-0.439	-0.439	-0.439	-0.439	-0.439	1.991	-0.439	2.723	-0.439	0.556
246	C_0127	Dyphylline	C07819	No ID	255.107	22.83	-0.651	-1.290	-1.078	-0.205	-0.592	-0.038	-0.792	-0.992	0.630	0.375	1.614	-0.654	1.853	0.700	1.119
247	C_0029	Homoserine	C00263	HMDB00719	120.066	10.38	-0.692	-0.692	-0.692	-0.692	-0.692	0.286	-0.692	-0.030	-0.692	-0.692	1.035	-0.692	2.053	1.725	1.159
248	C_0111	β-Ala-Lys	C05341	No ID	218.150	7.05	-0.479	-0.479	-0.479	-0.479	-0.479	-0.479	-0.479	-0.479	-0.479	-0.479	1.549	-0.479	2.029	2.174	-0.479
249	A_0036	Uric acid	C00366	HMDB00289	167.021	9.11	-1.636	-0.304	-0.588	-0.269	-0.538	0.892	-0.334	0.446	-0.142	-0.592	1.682	0.442	1.416	1.159	-1.636
250	A_0074	*myo*-Inositol 1-phosphate<br>*myo*-Inositol 3-phosphate	C01177<br>C04006	HMDB00213<br>HMDB06814	259.021	10.58	-1.327	0.997	-0.821	-1.112	-0.818	1.126	0.702	-0.945	-0.427	-0.921	1.801	0.001	1.161	0.625	-0.041
251	A_0029	Pelargonic acid	C01601	HMDB00847	157.123	8.29	-1.689	1.768	-0.787	-1.168	-0.765	0.441	0.619	-0.505	-0.280	-0.960	1.148	0.134	1.091	1.043	-0.089
252	C_0139	Glutathione (GSSG)_divalent	C00127	HMDB03337	307.082	12.52	-1.224	0.708	-1.105	-1.251	-0.821	0.045	-0.154	-0.366	-0.536	-0.840	1.876	0.758	1.508	0.830	0.573
253	C_0145	*S*-Adenosylhomocysteine	C00021	HMDB00939	385.129	8.97	-1.344	-0.628	-1.110	-1.352	-0.766	0.791	-0.301	-0.682	0.770	-0.171	1.243	0.743	0.874	0.067	1.866
254	C_0120	Cytidine	C00475	HMDB00089	244.093	9.96	-1.703	-0.223	-0.740	-1.464	-0.769	0.957	0.457	0.024	-0.389	-0.764	0.433	1.290	0.633	0.389	1.871
255	A_0065	Ribulose 5-phosphate	C00199,C0110	HMDB00618	229.011	11.36	-1.830	-0.286	-0.735	-1.405	-0.543	0.952	0.865	-0.596	0.069	-1.113	0.878	0.671	1.150	0.722	1.200
256	C_0022	Uracil	C00106	HMDB00300	113.035	22.80	-1.664	-0.473	-0.885	-1.306	-0.651	1.756	1.549	0.172	0.244	-0.690	-0.093	1.158	0.632	-0.240	0.490
257	C_0055	Hypoxanthine	C00262	HMDB00157	137.046	11.43	-1.313	-0.189	-0.732	-1.173	-0.645	1.755	1.620	-0.775	0.007	-1.041	0.115	1.360	0.797	0.133	0.081
258	A_0010	Hexanoic acid	C01585	HMDB00535	115.076	9.19	-0.558	0.769	-0.558	-0.558	-0.558	0.779	0.598	-0.558	0.405	-0.558	-0.558	-0.558	3.028	-0.558	-0.558

C indicates the cation mode and A indicates the anion mode.

† Metabolites identified from HMT database based on m/z and migration time
