# Supplementary material for: Altered choline level in atherosclerotic lesions: Upregulation of choline transporter-like protein 1 in human coronary unstable plaque
Source: PLoS One. 2023 Feb 17;18(2):e0281730. doi: 10.1371/journal.pone.0281730 (PMC9937458; doi:10.1371/journal.pone.0281730)
Supplement: S5 Table — (PDF) [file pone.0281730.s005.PDF]

Supplementary table 5. Principal component 1 and 2 from rabbits fed a 0.5% cholesterol diet

ID	HMT DB [†]			*m/z*	MT/RT	PC1	PC2
	Compound name	KEGG ID	HMDB ID				
A_0001	Glyoxylic acid	C00048	HMDB00119	72.993	12.44	-2.9E-02	8.2E-02
A_0003	Pyruvic acid	C00022	HMDB00243	87.008	13.57	1.7E-02	1.2E-02
A_0004	Lactic acid	C00186,C00256,C014	HMDB00190,HMDB01311	89.024	11.18	8.1E-02	2.7E-02
A_0005	3-Hydroxybutyric acid	C01089,C03197	HMDB00011,HMDB00357,HMDB00442	103.040	9.78	8.2E-02	1.9E-02
A_0006	2-Hydroxybutyric acid	C05984	HMDB00008	103.040	10.33	-5.3E-02	1.1E-01
A_0007	Glyceric acid	C00258	HMDB00139,HMDB06372	105.019	10.87	-6.6E-02	8.0E-02
A_0008	Fumaric acid	C00122	HMDB00134	115.003	27.83	8.2E-02	1.1E-02
A_0010	Hexanoic acid	C01585	HMDB00535	115.076	9.28	-3.6E-02	6.7E-02
A_0011	*N*-Acetylglycine	No ID	HMDB00532	116.035	9.81	3.9E-02	3.3E-02
A_0012	Succinic acid	C00042	HMDB00254	117.019	22.74	7.6E-02	8.3E-03
A_0013	Isethionic acid	C05123	HMDB03903	124.992	12.02	8.3E-02	8.6E-03
A_0014	5-Oxoproline	C01879	HMDB00267	128.035	9.73	8.0E-02	9.9E-03
A_0015	4-Methyl-2-oxovaleric acid<br>3-Methyl-2-oxovaleric acid	C00233<br>C00671,C03465	HMDB00695<br>HMDB00491	129.055	9.95	-5.3E-02	1.1E-02
A_0016	5-Oxohexanoic acid	C02129	No ID	129.055	9.45	-2.2E-02	-7.9E-03
A_0017	Heptanoic acid	No ID	HMDB00666	129.091	8.91	-2.5E-02	3.3E-02
A_0018	*N*-Acetylalanine	No ID	HMDB00766	130.050	9.22	-6.2E-02	1.0E-01
A_0019	6-Hydroxyhexanoic acid	C06103	No ID	131.069	8.56	3.1E-02	1.4E-02
A_0020	Malic acid	C00149,C00497,C007	HMDB00156,HMDB00744	133.014	23.17	8.2E-02	9.5E-03
A_0021	Threonic acid	C01620	HMDB00943	135.031	9.20	8.3E-02	5.8E-03
A_0022	Ethanolamine phosphate	C00346	HMDB00224	140.011	8.19	-3.8E-02	4.0E-02
A_0023	Octanoic acid	C06423	HMDB00482	143.107	8.49	5.7E-02	6.9E-02
A_0024	XA0004	-	-	144.029	9.29	6.9E-02	2.3E-03
A_0026	Adipic acid	C06104	HMDB00448	145.049	16.13	-5.9E-02	-4.6E-02
A_0027	2-Hydroxyglutaric acid	C02630,C01087,C031	HMDB00606,HMDB00694	147.029	16.98	8.0E-02	-4.8E-04
A_0028	Pelargonic acid	C01601	HMDB00847	157.123	8.27	4.2E-02	9.9E-02
A_0029	Pimelic acid	C02656	HMDB00857	159.065	14.71	-5.2E-02	3.0E-02
A_0030	8-Hydroxyoctanoic acid	No ID	No ID	159.101	8.04	5.8E-02	5.3E-03
A_0031	Terephthalic acid	C06337	HMDB02428	165.018	17.56	8.1E-02	3.7E-05
A_0032	Quinolinic acid	C03722	HMDB00232	166.014	17.06	-4.0E-02	1.1E-01
A_0033	XA0012	-	-	166.018	9.63	6.4E-02	9.5E-02
A_0034	Phosphoenolpyruvic acid	C00074	HMDB00263	166.974	23.63	-5.0E-02	-6.1E-02
A_0035	Uric acid	C00366	HMDB00289	167.021	9.13	-2.8E-02	2.9E-02
A_0036	Dihydroxyacetone phosphate	C00111	HMDB01473	168.990	13.23	7.6E-02	-6.6E-03
A_0037	Glyceraldehyde 3-phosphate	C00118,C00661	HMDB01112	168.989	11.63	3.0E-02	-1.6E-02
A_0038	Glycerol 3-phosphate	C00093	HMDB00126	171.006	12.63	8.2E-02	1.6E-02
A_0039	Decanoic acid	C01571	HMDB00511	171.138	8.21	-2.6E-02	1.1E-01
A_0040	Isovalerylalanine	No ID	HMDB00747	172.097	8.26	-6.8E-02	3.5E-02
A_0041	*cis*-Aconitic acid	C00417	HMDB00072	173.008	31.11	1.8E-02	-9.0E-02
A_0042	Suberic acid	C08278	HMDB00893	173.081	13.70	-7.6E-02	-1.3E-02
A_0043	*N*-Acetylaspartic acid	C01042	HMDB00812	174.040	15.37	-6.1E-02	-1.1E-01
A_0044	Ascorbic acid	C00072	HMDB00044	175.024	8.79	-2.4E-02	9.1E-02
A_0045	Hippuric acid	C01586	HMDB00714	178.050	8.34	5.2E-02	9.7E-03
A_0046	2-Phosphoglyceric acid	C00631	HMDB03391	184.985	21.25	-7.0E-02	-6.1E-02
A_0047	3-Phosphoglyceric acid	C00197	HMDB00807	184.985	21.29	-5.9E-02	-6.5E-02
A_0048	XA0017	-	-	186.113	7.97	-2.2E-02	5.2E-03
A_0049	*N*-Acetylglutamine	No ID	HMDB06029	187.073	8.02	5.0E-02	1.4E-02
A_0050	Azelaic acid	C08261	HMDB00784	187.097	12.91	-7.5E-02	-4.5E-02
A_0051	10-Hydroxydecanoic acid	C02774	No ID	187.133	7.71	8.2E-02	4.6E-03
A_0052	Isocitric acid	C00311	HMDB00193	191.019	32.07	4.3E-02	-3.9E-02
A_0053	XA0019	-	-	191.019	8.47	-4.3E-02	1.3E-01
A_0054	Citric acid	C00158	HMDB00094	191.019	28.50	7.4E-03	-9.7E-02
A_0055	Phenaceturic acid	C05598	HMDB00821	192.066	8.13	4.9E-02	-2.0E-03

A_0056	Gluconic acid	C00257	HMDB00625	195.050	8.35	-2.6E-02	-4.5E-02
A_0058	Sebacic acid	C08277	HMDB00792	201.112	12.27	-6.6E-02	1.8E-02
A_0059	Mucic acid	C00879,C01807	HMDB00639	209.029	15.14	-5.0E-02	1.3E-01
A_0060	Phosphocreatine	C02305	HMDB01511	210.028	12.83	-4.3E-02	-6.7E-02
A_0061	3-Indoxylsulfuric acid	No ID	HMDB00682	212.003	9.75	4.8E-02	2.6E-02
A_0062	*O*-Succinylhomoserine	C01118	No ID	218.069	12.98	-3.7E-02	1.3E-01
A_0063	Pantothenic acid	C00864	HMDB00210	218.103	7.78	3.0E-02	6.4E-03
A_0064	Myristoleic acid	C08322	HMDB02000	225.185	7.69	-4.6E-02	1.0E-01
A_0065	XA0027	-	-	227.200	7.36	4.4E-02	-4.7E-03
A_0066	Ribulose 5-phosphate	C00199,C01101	HMDB00618	229.011	11.36	5.5E-02	7.5E-02
A_0067	Ribose 5-phosphate	C00117	HMDB01548	229.011	10.91	-2.4E-02	4.9E-02
A_0068	XA0033	-	-	242.079	7.76	7.8E-02	1.6E-02
A_0069	Biotin	C00120	HMDB00030	243.081	7.55	8.1E-02	7.4E-03
A_0070	Glucosamine 6-phosphate	C00352	HMDB01254	258.038	8.85	6.6E-02	-1.3E-03
A_0071	Glucose 1-phosphate	C00103	HMDB01586	259.021	10.45	7.3E-02	-1.3E-02
A_0072	Glucose 6-phosphate	C00668,C01172,C00	HMDB01401	259.021	10.12	7.9E-02	4.1E-03
A_0073	*myo*-Inositol 2-phosphate	No ID	No ID	259.021	10.84	7.6E-02	4.3E-02
A_0074	Fructose 6-phosphate	C05345,C00085	HMDB00124	259.021	10.12	7.6E-02	1.4E-02
A_0075	*myo*-Inositol 1-phosphate<br>*myo*-Inositol 3-phosphate	C01177<br>C04006	HMDB00213<br>HMDB06814	259.021	10.57	4.6E-02	3.5E-02
A_0076	2,3-Diphosphoglyceric acid	C01159	HMDB01294	264.951	19.84	2.8E-02	-3.9E-02
A_0077	6-Phosphogluconic acid	C00345	HMDB01316	275.016	15.49	-4.1E-02	-1.2E-02
A_0078	Xanthosine	C01762	HMDB00299	283.066	7.77	-4.8E-02	8.6E-02
A_0079	Sedoheptulose 7-phosphate	C05382	HMDB01068	289.031	9.92	-4.1E-02	1.1E-01
A_0080	*N*-Acetylglucosamine 1-phosphate	C04256	HMDB01367	300.048	9.94	-6.2E-02	1.0E-01
A_0081	*N*-Acetylglucosamine 6-phosphate	C00357	HMDB01062	300.048	9.40	-4.2E-02	1.2E-01
A_0082	*N*-Acetylneuraminic acid	C00270	HMDB00230	308.098	7.25	7.1E-02	6.2E-02
A_0083	Ribulose 1,5-diphosphate	C01182	No ID	308.977	17.01	-5.2E-02	7.5E-02
A_0085	CMP	C00055	HMDB00095	322.043	9.83	7.9E-02	-2.4E-02
A_0086	UMP	C00105	HMDB00288	323.027	10.07	8.1E-02	3.3E-03
A_0087	cAMP	C00575	HMDB00058	328.045	7.43	8.2E-02	3.5E-03
A_0088	Fructose 1,6-diphosphate	C00354	HMDB01058	338.988	15.37	8.1E-02	5.6E-03
A_0089	cGMP	C00942	HMDB01314	344.039	7.51	7.0E-02	-9.6E-04
A_0090	AMP	C00020	HMDB00045	346.055	9.51	8.2E-02	7.5E-03
A_0091	IMP	C00130	HMDB00175	347.039	9.80	7.2E-02	-3.4E-03
A_0092	GMP	C00144	HMDB01397	362.050	9.38	8.1E-02	-1.7E-03
A_0093	XA0055	-	-	368.995	15.42	-3.1E-02	9.2E-02
A_0094	CoA_divalent	C00010	HMDB01423	382.549	10.96	7.7E-02	7.5E-03
A_0096	FAD_divalent	C00016	HMDB01248	391.571	7.99	8.2E-02	8.2E-03
A_0098	CDP	C00112	HMDB01546	402.010	11.85	4.4E-02	-8.9E-02
A_0099	UDP	C00015	HMDB00295	402.994	12.07	4.1E-02	-8.8E-02
A_0100	Acetyl CoA_divalent	C00024	HMDB01206	403.554	10.56	5.6E-02	-1.2E-02
A_0101	Cholic acid	C00695	HMDB00619	407.279	6.90	4.2E-02	5.8E-02
A_0102	ThPP	C00068	HMDB01372	423.029	7.88	8.3E-02	6.6E-03
A_0104	3',5'-ADP	C00054	HMDB00061	426.020	14.30	-7.5E-02	-1.9E-02
A_0105	ADP	C00008	HMDB01341	426.021	11.23	7.8E-02	-1.2E-02
A_0106	GDP	C00035	HMDB01201	442.016	11.00	7.9E-02	-3.6E-02
A_0107	XA0065	-	-	445.052	7.03	4.1E-02	-4.2E-02
A_0108	Octanoyl CoA_divalent	C01944	HMDB01070	445.602	9.53	6.0E-02	6.7E-03
A_0109	FMN	C00061	HMDB01520	455.099	8.26	7.9E-02	5.4E-03
A_0110	Adenylosuccinic acid	C03794	HMDB00536	462.066	14.40	7.8E-02	7.7E-03
A_0114	UTP	C00075	HMDB00285	482.959	13.10	4.7E-02	-1.5E-02
A_0115	CDP-choline	C00307	HMDB01413	487.100	6.83	6.4E-02	8.3E-02
A_0117	ATP	C00002	HMDB00538	505.988	12.19	6.1E-02	-1.8E-03
A_0118	GTP	C00044	HMDB01273	521.983	11.86	7.5E-02	-4.2E-03
A_0119	ADP-ribose	C00301	HMDB01178	558.064	8.68	8.2E-02	-4.3E-03

A_0120	UDP-glucose<br>UDP-galactose	C00029<br>C00052	HMDB00286<br>HMDB00302	565.048	8.83	7.7E-02	1.7E-03
A_0121	UDP-glucuronic acid	C00167	HMDB00935	579.027	11.40	3.6E-02	5.8E-02
A_0122	ADP-glucose<br>GDP-fucose	C00498<br>C00325	HMDB06557<br>HMDB01095	588.075	8.48	8.2E-02	4.4E-03
A_0123	GDP-galactose<br>GDP-mannose	C02280<br>C00096	No ID<br>HMDB01163	604.070	8.46	6.6E-02	-5.7E-02
A_0124	UDP-*N*-acetylglucosamine	C00043	HMDB00290	606.076	8.63	8.1E-02	5.0E-03
A_0125	CMP-*N*-acetylneuraminate	C00128	HMDB01176	613.140	8.09	8.3E-02	5.2E-03
A_0126	NAD^+	C00003	HMDB00902	662.103	6.61	-6.7E-02	-5.5E-02
A_0127	3'-Dephospho CoA	C00882	HMDB01373	686.140	7.93	3.1E-02	1.4E-02
A_0128	$NADP^+$	C00006	HMDB00217	742.071	9.75	-5.9E-02	-7.5E-02
C_0001	Urea	C00086	HMDB00294	61.040	21.79	8.2E-02	1.5E-02
C_0002	Ethanolamine	C00189	HMDB00149	62.060	6.53	-3.6E-02	1.3E-01
C_0003	XC0001	-	-	72.081	6.56	4.1E-02	5.8E-02
C_0004	Methylguanidine	C02294	HMDB01522	74.071	6.36	4.6E-02	-8.5E-04
C_0005	Gly	C00037	HMDB00123	76.040	8.55	2.0E-02	2.8E-02
C_0006	Trimethylamine *N*-oxide	C01104	HMDB00925	76.076	6.79	3.3E-02	3.5E-02
C_0007	Morpholine	C14452	HMDB31581	88.076	6.82	8.1E-02	2.4E-03
C_0008	Putrescine	C00134	HMDB01414	89.107	4.85	6.8E-02	6.5E-02
C_0009	Sarcosine	C00213	HMDB00271	90.055	9.73	-7.4E-02	4.9E-02
C_0010	Ala	C00041,C00133,C01	HMDB00161,HMDB01310	90.055	9.28	8.1E-02	1.2E-02
C_0011	β-Ala	C00099	HMDB00056	90.055	7.55	-5.8E-02	9.6E-02
C_0012	Glycerol	C00116	HMDB00131	93.055	22.74	-5.9E-02	-8.6E-02
C_0013	Homoserinelactone	No ID	No ID	102.055	7.30	7.0E-02	1.3E-02
C_0014	*N*,*N*-Dimethylglycine	C01026	HMDB00092	104.071	11.24	7.0E-02	2.7E-02
C_0015	2-Aminoisobutyric acid	C03665	HMDB01906	104.071	9.91	2.8E-02	1.0E-01
C_0016	GABA	C00334	HMDB00112	104.071	7.92	-7.4E-02	5.3E-02
C_0017	Choline	C00114	HMDB00097	104.107	7.05	-6.7E-02	8.8E-02
C_0018	Ser	C00065,C00716,C00	HMDB00187,HMDB03406	106.050	10.27	5.8E-02	9.1E-02
C_0019	Diethanolamine	C06772	HMDB04437	106.086	7.87	-2.7E-02	-5.0E-02
C_0020	Hypotaurine	C00519	HMDB00965	110.027	18.66	5.7E-02	2.5E-02
C_0022	Histamine	C00388	HMDB00870	112.086	4.91	7.2E-02	7.1E-04
C_0023	Uracil	C00106	HMDB00300	113.034	22.76	-2.5E-03	1.4E-01
C_0024	Creatinine	C00791	HMDB00562	114.066	7.51	8.1E-02	1.0E-02
C_0025	Pro	C00148,C00763,C16	HMDB00162,HMDB03411	116.070	11.11	4.5E-02	1.3E-01
C_0026	Guanidoacetic acid	C00581	HMDB00128	118.061	8.47	5.5E-02	2.1E-02
C_0027	Val	C00183,C06417,C16	HMDB00883	118.086	10.31	4.1E-02	1.3E-01
C_0028	Betaine	C00719	HMDB00043	118.086	11.59	7.7E-02	1.0E-02
C_0029	Thr	C00188,C00820	HMDB00167	120.065	10.81	6.7E-02	8.1E-02
C_0030	Homoserine	C00263	HMDB00719	120.066	10.37	1.2E-03	1.3E-01
C_0031	Betaine aldehyde_+H_2O	C00576	HMDB01252	120.102	7.67	-4.2E-02	1.3E-01
C_0032	Anserine_divalent	C01262	HMDB00194	121.068	7.04	7.5E-02	-3.6E-02
C_0033	Cys	C00097,C00736,C00	HMDB00574,HMDB03417	122.027	11.60	-4.9E-02	-5.9E-02
C_0034	2-Amino-2-(hydroxymethyl)-1,3-propanediol	C07182	No ID	122.081	8.54	-2.3E-02	-4.2E-02
C_0035	2-Phenylethylamine	C05332	HMDB12275	122.096	8.05	-1.2E-02	-4.1E-02
C_0036	Nicotinamide	C00153	HMDB01406	123.055	7.61	8.3E-02	6.7E-03
C_0037	Taurine	C00245	HMDB00251	126.022	22.72	8.3E-02	9.7E-03
C_0038	1-Methylhistamine	C05127	HMDB00898	126.102	5.07	-5.4E-02	9.7E-02
C_0040	XC0016	-	-	129.066	9.00	8.2E-02	1.8E-02
C_0041	Pipecolic acid	C00408	HMDB00070,HMDB00716,HMDB05960	130.086	10.58	5.0E-02	4.0E-02
C_0042	*N*-Methylproline	No ID	No ID	130.086	12.80	2.8E-02	1.1E-02
C_0043	*N*-Acetylputrescine	C02714	HMDB02064	131.117	8.77	-4.8E-02	9.7E-02
C_0044	Hydroxyproline	C01157	HMDB00725	132.065	12.38	7.0E-02	5.5E-02
C_0045	3-Guanidinopropionic acid	C03065	No ID	132.076	8.25	7.6E-02	1.2E-02
C_0046	Creatine	C00300	HMDB00064	132.410	9.06	-6.0E-02	-5.8E-02
C_0047	Ile	C00407,C06418,C16	HMDB00172	132.102	10.51	-2.7E-03	1.5E-01
C_0048	Leu	C00123,C01570,C16	HMDB00687	132.102	10.61	3.1E-02	1.4E-01

C_0049	Gly-Gly	C02037	HMDB11733	133.060	8.63	8.3E-02	4.5E-03
C_0050	Asn	C00152,C01905,C164	HMDB00168	133.061	10.78	8.0E-02	3.1E-02
C_0051	Ornithine	C00077,C00515,C01€	HMDB00214,HMDB03374	133.097	7.01	7.3E-02	3.0E-02
C_0052	Thiaproline	No ID	No ID	134.027	14.32	-5.5E-02	1.0E-01
C_0053	Asp	C00049,C00402,C164	HMDB00191,HMDB06483	134.045	11.88	7.4E-02	3.1E-02
C_0054	Adenine	C00147	HMDB00034	136.062	7.84	7.1E-02	3.0E-02
C_0055	Hypoxanthine	C00262	HMDB00157	137.046	11.43	7.6E-03	1.3E-01
C_0057	Trigonelline	C01004	HMDB00875	138.056	10.78	6.7E-02	-1.4E-04
C_0058	Tyramine	C00483	HMDB00306	138.091	8.56	-3.0E-02	6.6E-02
C_0059	Urocanic acid	C00785	HMDB00301	139.049	8.51	-3.9E-02	-3.1E-02
C_0060	1-Methyl-4-imidazoleacetic acid	C05828	HMDB02820	141.066	8.49	4.8E-02	1.4E-02
C_0061	Ectoine	C06231	No ID	143.080	9.75	3.5E-02	4.4E-03
C_0062	XC0029	-	-	144.102	12.58	2.4E-02	5.3E-03
C_0063	Stachydrine	C10172	HMDB04827	144.102	11.84	7.7E-02	2.0E-02
C_0064	Acetylcholine	C01996	HMDB00895	146.117	7.84	5.0E-02	1.3E-02
C_0065	γ-Butyrobetaine	C01181	HMDB01161	146.117	8.32	8.2E-02	3.9E-03
C_0066	Spermidine	C00315	HMDB01257	146.165	4.66	7.1E-02	-2.5E-02
C_0067	Gln	C00064,C00303,C00ε	HMDB00641,HMDB03423	147.076	11.04	8.2E-02	4.3E-03
C_0068	Lys	C00047,C00739,C164	HMDB00182,HMDB03405	147.113	7.08	2.5E-02	1.3E-01
C_0069	*threo*-β-Methylaspartic acid	C03618	No ID	148.060	12.73	7.6E-02	7.5E-03
C_0070	Glu	C00025,C00217,C003	HMDB00148,HMDB03339	148.060	11.24	7.0E-02	3.4E-02
C_0071	Isoglutamic acid	C05574	No ID	148.060	9.41	8.1E-02	2.5E-02
C_0072	Met	C00073,C00855,C017	HMDB00696	150.058	11.01	-1.6E-02	1.4E-01
C_0073	Triethanolamine	C06771	No ID	150.112	8.48	-3.2E-02	-2.8E-02
C_0074	Guanine	C00242	HMDB00132	152.057	8.54	-5.0E-02	1.2E-01
C_0075	His	C00135,C00768,C064	HMDB00177	156.077	7.51	7.5E-02	1.3E-02
C_0076	Ala-Ala	C00993	HMDB03459	161.091	9.52	-5.2E-02	1.2E-01
C_0077	N^6-Methyllysine	C02728	HMDB02038	161.128	7.31	6.4E-02	5.6E-02
C_0078	2-Aminoadipic acid	C00956	HMDB00510	162.076	11.27	6.6E-02	6.6E-02
C_0079	Carnitine	C00318,C00487,C15(	HMDB00062	162.112	8.75	8.0E-02	1.4E-02
C_0080	5-Hydroxylysine	C16741	HMDB00450	163.107	7.36	-4.2E-02	1.3E-01
C_0081	Methionine sulfoxide	C02989	HMDB02005	166.052	12.28	5.0E-02	1.3E-02
C_0082	Phe	C00079,C02057,C022	HMDB00159	166.085	11.40	6.6E-02	9.7E-02
C_0083	Taurocyamine	C01959	HMDB03584	168.042	22.99	8.2E-02	6.7E-03
C_0084	Tyr-Arg_divalent	No ID	No ID	169.594	7.94	-4.5E-02	1.3E-01
C_0085	Noradrenaline	C00547	HMDB00216	170.081	9.27	4.3E-02	2.8E-02
C_0086	1-Methylhistidine	No ID	HMDB00001	170.092	7.72	8.3E-02	6.3E-03
C_0087	3-Methylhistidine	C01152	HMDB00479	170.092	7.71	-7.6E-02	5.9E-02
C_0088	XC0040	-	-	174.087	12.55	8.2E-02	1.7E-03
C_0089	*N*-Acetylornithine	C00437	HMDB03357	175.107	9.79	-5.0E-02	1.2E-01
C_0090	N^5-Ethylglutamine	C01047	No ID	175.109	11.70	7.5E-02	2.8E-02
C_0091	Arg	C00062,C00792	HMDB00517,HMDB03416	175.119	7.32	7.7E-02	5.0E-02
C_0092	Citrulline	C00327	HMDB00904	176.102	11.37	6.8E-02	5.7E-02
C_0093	Serotonin	C00780	HMDB00259	177.102	9.00	-1.6E-03	3.8E-02
C_0094	Glucosamine	C00329	HMDB01514	180.087	9.56	6.5E-02	1.4E-02
C_0095	Tyr	C00082,C01536,C064	HMDB00158	182.081	11.68	3.7E-02	1.5E-01
C_0096	Phosphorylcholine	C00588	HMDB01565	184.072	21.17	7.6E-02	2.3E-02
C_0097	N^8-Acetylspermidine	C01029	HMDB02189	188.176	6.52	-4.1E-02	1.1E-01
C_0098	N^6-Acetyllysine	C02727	HMDB00206	189.123	11.81	3.0E-02	-1.6E-02
C_0099	Gly-Leu	No ID	No ID	189.123	10.12	-4.9E-02	1.1E-01
C_0100	*N*-Acetyllysine	C12989	HMDB00446	189.123	10.18	8.2E-02	3.0E-03
C_0101	N^6,N^6,N^6-Trimethyllysine	C03793	HMDB01325	189.159	7.39	-7.1E-02	6.0E-02
C_0102	Homocitrulline	C02427	HMDB00679	190.118	11.53	7.9E-02	3.9E-03
C_0103	Gly-Asp	No ID	No ID	191.065	10.25	8.1E-02	-4.5E-03
C_0104	ADMA	C03626	HMDB01539	203.149	7.89	1.9E-02	7.7E-02
C_0105	SDMA	No ID	HMDB03334	203.150	8.02	7.9E-02	3.2E-02

C_0106	Spermine	C00750	HMDB01256	203.223	4.60	5.7E-02	-8.3E-02
C_0107	*O*-Acetylcarnitine	C02571	HMDB00201	204.122	9.28	8.1E-02	5.7E-03
C_0108	Trp	C00078,C00525,C00	HMDB00929	205.097	11.33	6.4E-02	9.8E-02
C_0109	Carboxymethyllysine	No ID	No ID	205.119	9.50	8.2E-02	7.3E-03
C_0110	Kynurenine	C00328,C01718	HMDB00684	209.091	10.17	7.5E-02	9.5E-03
C_0111	XC0061	-	-	218.138	9.58	7.4E-02	1.5E-02
C_0112	β-Ala-Lys	C05341	No ID	218.149	7.00	-1.1E-02	1.3E-01
C_0113	Cystathionine	C00542,C02291	HMDB00099	223.075	10.19	5.9E-02	1.7E-02
C_0114	Carnosine	C00386	HMDB00033	227.113	6.95	8.0E-02	6.7E-03
C_0115	2'-Deoxycytidine	C00881	HMDB00014	228.097	9.68	-6.0E-02	9.9E-02
C_0116	Ergothioneine	C05570	HMDB03045	230.095	18.08	3.1E-02	1.4E-02
C_0117	Butyrylcarnitine	C02862	HMDB02013	232.153	9.85	7.7E-02	5.6E-03
C_0118	XC0071	-	-	234.180	6.40	8.3E-02	6.0E-03
C_0119	Cystine	C00491,C01420	HMDB00192	241.031	11.28	-3.6E-02	4.9E-02
C_0120	Homocarnosine	C00884	HMDB00745	241.129	7.04	7.3E-02	-3.6E-02
C_0122	Cytidine	C00475	HMDB00089	244.093	9.95	5.2E-02	9.9E-02
C_0123	Uridine	C00299	HMDB00296	245.076	22.78	-3.3E-02	1.4E-01
C_0124	N^{1}-Acetylspermine	C02567	HMDB01186	245.233	5.72	-3.8E-02	-2.3E-02
C_0125	Malonylcarnitine	No ID	HMDB02095	248.111	10.54	7.9E-02	2.9E-03
C_0126	Pyridoxamine-P	C00647	HMDB01555	249.063	10.80	8.3E-02	5.0E-03
C_0127	γ-Glu-Cys	C00669	HMDB01049	251.069	13.23	8.0E-02	8.8E-03
C_0128	XC0089	-	-	255.097	9.76	-5.0E-02	7.7E-02
C_0129	Dyphylline	C07819	No ID	255.107	22.79	2.8E-02	7.9E-02
C_0130	Glycerophosphocholine	C00670	HMDB00086	258.109	22.32	8.0E-02	2.4E-02
C_0131	Thiamine	C00378	HMDB00235	265.110	6.78	8.1E-02	6.2E-03
C_0132	Adenosine	C00212	HMDB00050	268.104	10.14	-4.3E-02	5.8E-02
C_0133	Inosine	C00294	HMDB00195	269.087	20.11	-4.8E-02	1.0E-03
C_0134	Glu-Glu	C01425	No ID	277.103	11.19	-4.8E-02	9.8E-02
C_0135	Saccharopine	C00449	HMDB00279	277.139	11.05	7.8E-02	-5.6E-04
C_0136	Guanosine	C00387	HMDB00133	284.098	12.88	-7.6E-02	3.6E-02
C_0137	Octanoylcarnitine	C02838	HMDB00791	288.215	10.73	7.3E-02	-3.6E-03
C_0138	Ophthalmic acid	No ID	HMDB05765	290.134	13.56	-6.5E-02	8.8E-02
C_0139	Argininosuccinic acid	C03406	HMDB00052	291.130	9.72	8.1E-02	7.7E-03
C_0140	5'-Deoxy-5'-methylthioadenosine	C00170	HMDB01173	298.097	10.41	7.8E-02	9.1E-03
C_0141	Glutathione (GSSG)_divalent	C00127	HMDB03337	307.083	12.52	7.5E-02	4.6E-02
C_0142	Glutathione (GSH)	C00051	HMDB00125	308.090	13.60	7.9E-02	-2.6E-02
C_0143	XC0132	-	-	325.160	8.93	-6.7E-02	9.4E-02
C_0144	NMN	C00455	HMDB00229	335.064	21.20	-7.3E-02	6.8E-02
C_0145	TMP	C01081	HMDB02666	345.077	11.01	5.7E-02	9.9E-02
C_0146	*S*-Lactoylglutathione	C03451	HMDB01066	380.112	14.24	7.0E-02	1.5E-02
C_0147	*S*-Adenosylhomocysteine	C00021	HMDB00939	385.129	8.98	3.6E-02	1.3E-01
C_0148	*S*-Adenosylmethionine	C00019	HMDB01185	399.144	7.32	7.8E-02	1.6E-02
C_0149	Cysteine glutathione disulfide	C05526	HMDB00656	427.095	11.94	-5.1E-02	8.5E-02

C indicates the cation mode and A indicates the anion mode.

† Metabolites identified from HMT database based on m/z and migration time
