# Supplementary material for: Altered choline level in atherosclerotic lesions: Upregulation of choline transporter-like protein 1 in human coronary unstable plaque
Source: PLoS One. 2023 Feb 17;18(2):e0281730. doi: 10.1371/journal.pone.0281730 (PMC9937458; doi:10.1371/journal.pone.0281730)
Supplement: S6 Table — (PDF) [file pone.0281730.s006.PDF]

Supplementary table 6. Hierarchical clustering analysis of arterial and cardiac metabolites in rabbits fed a 0.5% cholesterol diet

| Line | ID | HMT DB † | | | *m/z* | MT/RT | Standardized Relative Area | | | | | | | | | | | | | | |
|---|---|---|---|---|---|---|---|---|---|---|---|---|---|---|---|---|---|---|---|---|---|
| | | | | | | | non-injured artery | | | | | injured artery | | | | | heart | | | | |
| | | Compound name | KEGG ID | HMDB ID | | | 1 | 2 | 3 | 4 | 5 | 6 | 7 | 8 | 9 | 10 | 11 | 12 | 13 | 14 | 15 |
| 1 | C_0062 | XC0029 | - | - | 144.102 | 12.58 | -0.411 | -0.411 | 0.745 | -0.411 | -0.411 | -0.411 | -0.411 | 0.944 | -0.411 | -0.411 | -0.411 | -0.411 | 3.244 | -0.411 | -0.411 |
| 2 | C_0042 | *N*-Methylproline | No ID | No ID | 130.086 | 12.80 | -0.374 | -0.374 | 0.303 | -0.374 | -0.374 | -0.374 | -0.278 | 0.649 | -0.374 | -0.374 | -0.374 | -0.374 | 3.445 | -0.374 | -0.374 |
| 3 | C_0061 | Ectoine | C06231 | No ID | 143.080 | 9.75 | -0.258 | -0.258 | -0.258 | -0.258 | -0.258 | -0.258 | -0.258 | -0.258 | -0.258 | -0.258 | -0.258 | -0.258 | 3.615 | -0.258 | -0.258 |
| 4 | A_0055 | Phenaceturic acid | C05598 | HMDB00821 | 192.066 | 8.13 | -0.386 | -0.386 | -0.386 | -0.386 | -0.386 | -0.386 | -0.386 | -0.386 | -0.386 | -0.386 | 0.497 | -0.386 | 3.374 | 0.763 | -0.386 |
| 5 | A_0052 | Isocitric acid | C00311 | HMDB00193 | 191.019 | 32.07 | 0.356 | -0.126 | 0.875 | -1.456 | -1.361 | -0.193 | -0.920 | -1.074 | 0.447 | 0.145 | 1.018 | -0.290 | 2.389 | 0.187 | 0.002 |
| 6 | A_0041 | *cis*-Aconitic acid | C00417 | HMDB00072 | 173.008 | 31.11 | 1.566 | 0.024 | 2.108 | -1.247 | -1.230 | -0.181 | -0.809 | -1.039 | -0.044 | -0.283 | 0.281 | -0.424 | 1.379 | 0.048 | -0.147 |
| 7 | A_0054 | Citric acid | C00158 | HMDB00094 | 191.019 | 28.50 | 1.586 | 0.096 | 2.311 | -1.173 | -1.144 | -0.227 | -0.925 | -0.980 | 0.298 | -0.150 | 0.168 | -0.643 | 0.954 | 0.142 | -0.312 |
| 8 | A_0076 | 2,3-Diphosphoglyceric acid | C01159 | HMDB01294 | 264.951 | 19.84 | -0.213 | -0.573 | 2.687 | -0.764 | -1.125 | -0.794 | 0.451 | -0.565 | -0.595 | -0.532 | 0.349 | -0.529 | 1.431 | 0.488 | 0.284 |
| 9 | C_0106 | Spermine | C00750 | HMDB01256 | 203.223 | 4.60 | 0.261 | -0.059 | 1.774 | -1.055 | -1.101 | -0.934 | -1.127 | -1.206 | -0.543 | -0.462 | 0.979 | 0.581 | 1.190 | 1.161 | 0.541 |
| 10 | A_0098 | CDP | C00112 | HMDB01546 | 402.010 | 11.85 | 1.246 | -0.631 | 1.531 | -0.834 | -0.779 | -0.713 | -0.922 | -0.974 | -0.698 | -0.688 | 0.447 | -0.083 | 0.073 | 1.931 | 1.094 |
| 11 | A_0099 | UDP | C00015 | HMDB00295 | 402.994 | 12.07 | 1.558 | -0.628 | 1.526 | -0.941 | -0.785 | -0.673 | -0.923 | -1.003 | -0.606 | -0.680 | 0.163 | 0.126 | -0.017 | 1.390 | 1.494 |
| 12 | A_0003 | Pyruvic acid | C00022 | HMDB00243 | 87.008 | 13.57 | 0.473 | -0.504 | 0.438 | -0.504 | -0.504 | -0.504 | 0.676 | -0.504 | 0.201 | -0.504 | -0.504 | -0.504 | -0.504 | -0.504 | 3.248 |
| 13 | C_0005 | Gly | C00037 | HMDB00123 | 76.040 | 8.55 | -0.173 | 0.091 | -0.376 | -1.366 | 0.207 | 0.006 | 0.250 | -0.376 | -0.350 | 0.513 | -0.068 | -0.577 | -0.870 | -0.106 | 3.196 |
| 14 | A_0011 | *N*-Acetylglycine | No ID | HMDB00532 | 116.035 | 9.81 | -0.283 | -0.784 | -0.784 | -0.784 | 0.129 | -0.132 | -0.158 | -0.464 | -0.164 | 0.165 | 1.356 | 0.585 | -0.784 | -0.784 | 2.887 |
| 15 | C_0060 | 1-Methyl-4-imidazoleacetic acid | C05828 | HMDB02820 | 141.066 | 8.49 | -0.188 | -0.617 | -1.201 | -0.957 | 0.265 | 0.663 | -0.871 | -0.677 | -0.794 | 0.415 | 1.645 | -0.015 | -0.276 | 0.184 | 2.424 |
| 16 | A_0101 | Cholic acid | C00695 | HMDB00619 | 407.279 | 6.90 | -1.156 | -1.156 | -1.156 | -0.650 | 0.736 | -0.088 | -0.229 | -0.478 | -0.291 | 1.142 | 0.815 | 0.934 | 0.756 | -1.156 | 1.976 |
| 17 | A_0075 | *myo*-Inositol 1-phosphate<br>*myo*-Inositol 3-phosphate | C01177<br>C04006 | HMDB00213<br>HMDB06814 | 259.021 | 10.57 | -1.541 | -0.396 | -1.541 | 0.745 | 1.136 | -0.551 | -0.079 | 0.095 | -0.910 | -0.871 | 0.387 | 1.120 | -0.354 | 1.325 | 1.435 |
| 18 | A_0028 | Pelargonic acid | C01601 | HMDB00847 | 157.123 | 8.27 | -1.845 | -0.356 | -1.845 | -0.493 | 0.429 | -0.119 | 0.461 | 1.339 | -1.058 | -0.232 | 0.744 | 1.134 | 0.067 | 1.019 | 0.753 |
| 19 | A_0023 | Octanoic acid | C06423 | HMDB00482 | 143.107 | 8.49 | -1.542 | -0.177 | -1.542 | -0.511 | 0.111 | -0.399 | 0.168 | 1.006 | -1.542 | -0.441 | 0.700 | 1.290 | 0.909 | 1.337 | 0.633 |
| 20 | A_0066 | Ribulose 5-phosphate | C00199,C0110 | HMDB00618 | 229.011 | 11.36 | -1.489 | -0.161 | -1.067 | -0.105 | -0.374 | -0.280 | 1.160 | 0.050 | -1.141 | -1.269 | 0.888 | 2.035 | 0.710 | 0.247 | 0.796 |
| 21 | C_0122 | Cytidine | C00475 | HMDB00089 | 244.093 | 9.95 | -1.058 | -0.348 | -1.056 | -0.883 | -1.142 | -0.395 | 1.459 | 0.601 | -0.690 | -0.924 | 0.824 | 1.664 | 1.399 | 0.155 | 0.394 |
| 22 | C_0147 | *S*-Adenosylhomocysteine | C00021 | HMDB00939 | 385.129 | 8.98 | -1.712 | -0.893 | -1.398 | -0.902 | -0.784 | -0.481 | 1.347 | 1.201 | 0.467 | -0.096 | 1.159 | 1.137 | -0.077 | 0.469 | 0.562 |
| 23 | C_0145 | TMP | C01081 | HMDB02666 | 345.077 | 11.01 | -1.234 | -1.061 | -1.167 | -0.744 | -1.090 | -0.030 | 0.793 | 0.618 | -0.462 | -0.527 | 1.318 | 2.010 | 0.233 | 0.591 | 0.752 |
| 24 | A_0033 | XA0012 | - | - | 166.018 | 9.63 | -1.020 | -0.907 | -1.361 | -1.066 | -1.015 | 0.101 | 0.841 | 0.062 | -0.216 | -0.735 | 0.415 | 1.907 | 1.081 | 0.987 | 0.925 |
| 25 | C_0082 | Phe | C00079,C0205 | HMDB00159 | 166.085 | 11.40 | -1.413 | -1.152 | -1.057 | -1.173 | -1.071 | 0.172 | 0.627 | 0.190 | -0.220 | -0.362 | 0.682 | 0.895 | 1.513 | 1.047 | 1.322 |
| 26 | C_0108 | Trp | C00078,C0052 | HMDB00929 | 205.097 | 11.33 | -1.364 | -1.036 | -1.163 | -1.423 | -0.870 | 0.262 | 0.476 | 0.071 | -0.052 | -0.222 | 0.968 | 1.262 | 0.643 | 0.658 | 1.790 |
| 27 | C_0044 | Hydroxyproline | C01157 | HMDB00725 | 132.065 | 12.38 | -0.824 | -0.995 | -0.743 | -1.268 | -0.808 | 0.329 | 0.102 | -0.316 | -0.712 | -0.563 | 1.797 | 0.596 | 1.324 | 0.343 | 1.738 |
| 28 | C_0029 | Thr | C00188,C0082 | HMDB00167 | 120.065 | 10.81 | -1.115 | -1.187 | -1.081 | -1.327 | -0.815 | 0.174 | 0.082 | 0.037 | -0.045 | -0.172 | 1.043 | 0.480 | 1.512 | 0.447 | 1.969 |
| 29 | C_0018 | Ser | C00065,C0071 | HMDB00187,H | 106.050 | 10.27 | -1.193 | -0.907 | -1.329 | -1.055 | -0.924 | -0.142 | 0.676 | -0.161 | 0.444 | -0.231 | 0.445 | 0.380 | 0.552 | 1.131 | 2.312 |
| 30 | A_0082 | *N*-Acetylneuraminic acid | C00270 | HMDB00230 | 308.098 | 7.25 | -1.023 | -0.996 | -1.275 | -1.011 | -0.970 | -0.278 | -0.143 | -0.405 | 0.562 | -0.291 | 0.866 | 0.550 | 1.729 | 1.171 | 1.515 |
| 31 | C_0092 | Citrulline | C00327 | HMDB00904 | 176.102 | 11.37 | -1.082 | -0.553 | -0.990 | -1.079 | -0.573 | -0.344 | 0.227 | -0.675 | -0.351 | 0.021 | -0.078 | 0.976 | 2.139 | 0.482 | 1.880 |
| 32 | A_0115 | CDP-choline | C00307 | HMDB01413 | 487.100 | 6.83 | -1.120 | -1.007 | -0.834 | -1.113 | -1.020 | 0.142 | 0.299 | 0.294 | -0.425 | -0.318 | 0.144 | 1.154 | 2.267 | 0.221 | 1.318 |
| 33 | C_0078 | 2-Aminoadipic acid | C00956 | HMDB00510 | 162.076 | 11.27 | -1.376 | -0.895 | -0.418 | -1.386 | -0.869 | -0.615 | -0.283 | 0.619 | -0.430 | 0.346 | 0.716 | 1.041 | 1.865 | 0.206 | 1.480 |
| 34 | C_0077 | $N^6$-Methyllysine | C02728 | HMDB02038 | 161.128 | 7.31 | -1.389 | -0.701 | -0.605 | -0.777 | -0.843 | -0.187 | 0.026 | 0.108 | -0.225 | -0.597 | 0.648 | 0.503 | 2.816 | 0.834 | 0.388 |
| 35 | C_0066 | Spermidine | C00315 | HMDB01257 | 146.165 | 4.66 | -0.521 | -0.238 | 0.200 | -1.062 | -0.801 | -0.555 | -1.078 | -1.150 | 0.103 | -0.490 | 0.701 | 0.746 | 2.538 | 0.937 | 0.670 |
| 36 | A_0123 | GDP-galactose<br>GDP-mannose | C02280<br>C00096 | No ID<br>HMDB01163 | 604.070 | 8.46 | 0.209 | 0.447 | 0.573 | -0.940 | -0.679 | -0.618 | -0.818 | -1.282 | -0.889 | -1.114 | 0.369 | 0.674 | 2.199 | 0.634 | 1.237 |
| 37 | C_0120 | Homocarnosine | C00884 | HMDB00745 | 241.129 | 7.04 | -0.752 | -0.689 | 0.866 | -0.548 | -0.221 | -0.890 | -0.893 | -0.867 | -0.924 | -0.903 | 0.443 | 1.751 | 0.844 | 1.348 | 1.435 |
| 38 | C_0032 | Anserine_divalent | C01262 | HMDB00194 | 121.068 | 7.04 | -0.739 | -0.715 | 0.776 | -0.548 | -0.181 | -0.894 | -0.910 | -0.888 | -0.943 | -0.924 | 0.568 | 1.640 | 0.966 | 1.377 | 1.416 |
| 39 | A_0085 | CMP | C00055 | HMDB00095 | 322.043 | 9.83 | -0.719 | -0.734 | -0.215 | -0.833 | 0.057 | -0.566 | -1.116 | -1.156 | -0.519 | -0.671 | 1.468 | 1.356 | 1.188 | 1.504 | 0.955 |
| 40 | A_0086 | UMP | C00105 | HMDB00288 | 323.027 | 10.07 | -0.750 | -0.854 | -0.665 | -0.753 | -0.314 | -0.482 | -0.910 | -0.944 | -0.258 | -0.663 | 1.016 | 1.861 | 1.396 | 1.232 | 1.088 |
| 41 | A_0092 | GMP | C00144 | HMDB01397 | 362.050 | 9.38 | -0.740 | -0.810 | -0.533 | -0.695 | -0.283 | -0.523 | -0.943 | -0.926 | -0.491 | -0.669 | 1.408 | 1.790 | 1.335 | 0.812 | 1.268 |
| 42 | A_0124 | UDP-*N*-acetylglucosamine | C00043 | HMDB00290 | 606.076 | 8.63 | -0.834 | -0.487 | -0.454 | -1.044 | -0.647 | -0.202 | -0.663 | -1.024 | -0.625 | -0.632 | 1.499 | 1.685 | 1.245 | 1.176 | 1.007 |
| 43 | C_0071 | Isoglutamic acid | C05574 | No ID | 148.060 | 9.41 | -0.848 | -0.692 | -0.724 | -0.848 | -0.765 | -0.481 | -0.534 | -0.354 | -0.660 | -0.709 | 1.362 | 1.823 | 1.568 | 0.830 | 1.033 |
| 44 | C_0131 | Thiamine | C00378 | HMDB00235 | 265.110 | 6.78 | -0.670 | -0.670 | -0.670 | -0.670 | -0.670 | -0.670 | -0.670 | -0.670 | -0.670 | -0.670 | 1.836 | 1.579 | 1.199 | 0.914 | 1.172 |
| 45 | C_0102 | Homocitrulline | C02427 | HMDB00679 | 190.118 | 11.53 | -0.659 | -0.659 | -0.659 | -0.659 | -0.659 | -0.659 | -0.659 | -0.659 | -0.659 | -0.659 | 2.162 | 1.024 | 1.125 | 0.949 | 1.329 |
| 46 | C_0109 | Carboxymethyllysine | No ID | No ID | 205.119 | 9.50 | -0.670 | -0.670 | -0.670 | -0.670 | -0.670 | -0.670 | -0.670 | -0.670 | -0.670 | -0.670 | 1.419 | 1.104 | 1.201 | 1.046 | 1.933 |
| 47 | A_0069 | Biotin | C00120 | HMDB00030 | 243.081 | 7.55 | -0.668 | -0.668 | -0.668 | -0.668 | -0.668 | -0.668 | -0.668 | -0.668 | -0.668 | -0.668 | 1.453 | 1.493 | 0.791 | 1.142 | 1.807 |
| 48 | A_0013 | Isethionic acid | C05123 | HMDB03903 | 124.992 | 12.02 | -0.653 | -0.664 | -0.628 | -0.736 | -0.633 | -0.691 | -0.654 | -0.604 | -0.690 | -0.797 | 1.137 | 1.702 | 1.242 | 1.086 | 1.584 |
| 49 | C_0040 | XC0016 | - | - | 129.066 | 9.00 | -0.862 | -0.714 | -0.685 | -0.782 | -0.565 | -0.577 | -0.533 | -0.595 | -0.707 | -0.663 | 0.952 | 1.433 | 1.137 | 1.275 | 1.886 |
| 50 | A_0014 | 5-Oxoproline | C01879 | HMDB00267 | 128.035 | 9.73 | -0.629 | -0.511 | -0.771 | -0.817 | -0.852 | -0.595 | -0.629 | -0.481 | -0.673 | -0.680 | 1.521 | 0.890 | 0.900 | 1.582 | 1.744 |
| 51 | A_0051 | 10-Hydroxydecanoic acid | C02774 | No ID | 187.133 | 7.71 | -0.680 | -0.680 | -0.680 | -0.680 | -0.680 | -0.680 | -0.680 | -0.680 | -0.680 | -0.680 | 1.500 | 1.319 | 1.052 | 1.445 | 1.481 |
| 52 | A_0122 | ADP-glucose<br>GDP-fucose | C00498<br>C00325 | HMDB06557<br>HMDB01095 | 588.075 | 8.48 | -0.720 | -0.445 | -0.562 | -0.873 | -0.876 | -0.567 | -0.592 | -0.807 | -0.587 | -0.713 | 1.689 | 1.214 | 1.137 | 1.372 | 1.328 |
| 53 | C_0103 | Gly-Asp | No ID | No ID | 191.065 | 10.25 | -0.617 | -0.538 | -0.707 | -0.758 | -0.532 | -0.647 | -0.596 | -1.136 | -0.410 | -0.705 | 1.255 | 1.069 | 1.328 | 1.870 | 1.125 |
| 54 | A_0119 | ADP-ribose | C00301 | HMDB01178 | 558.064 | 8.68 | -0.699 | -0.634 | -0.508 | -0.648 | -0.686 | -0.696 | -0.719 | -0.769 | -0.620 | -0.763 | 1.545 | 0.957 | 1.450 | 1.623 | 1.166 |
| 55 | C_0088 | XC0040 | - | - | 174.087 | 12.55 | -0.674 | -0.674 | -0.674 | -0.674 | -0.674 | -0.674 | -0.674 | -0.674 | -0.674 | -0.674 | 1.336 | 0.945 | 1.432 | 1.773 | 1.257 |
| 56 | C_0065 | γ-Butyrobetaine | C01181 | HMDB01161 | 146.117 | 8.32 | -0.697 | -0.649 | -0.644 | -0.687 | -0.681 | -0.684 | -0.616 | -0.681 | -0.679 | -0.701 | 1.151 | 0.907 | 1.710 | 1.629 | 1.324 |
| 57 | A_0087 | cAMP | C00575 | HMDB00058 | 328.045 | 7.43 | -0.674 | -0.674 | -0.674 | -0.674 | -0.674 | -0.674 | -0.674 | -0.674 | -0.674 | -0.674 | 1.122 | 0.972 | 1.372 | 1.703 | 1.571 |
| 58 | C_0067 | Gln | C00064,C0030 | HMDB00641,H | 147.076 | 11.04 | -0.682 | -0.635 | -0.649 | -0.698 | -0.679 | -0.681 | -0.659 | -0.677 | -0.694 | -0.703 | 1.283 | 0.988 | 1.300 | 1.462 | 1.726 |
| 59 | C_0118 | XC0071 | - | - | 234.180 | 6.40 | -0.677 | -0.677 | -0.677 | -0.677 | -0.677 | -0.677 | -0.677 | -0.677 | -0.677 | -0.677 | 1.004 | 1.216 | 1.396 | 1.468 | 1.683 |
| 60 | C_0036 | Nicotinamide | C00153 | HMDB01406 | 123.055 | 7.61 | -0.744 | -0.696 | -0.677 | -0.643 | -0.672 | -0.704 | -0.611 | -0.661 | -0.692 | -0.721 | 1.342 | 1.235 | 1.401 | 1.495 | 1.346 |
| 61 | C_0086 | 1-Methylhistidine | No ID | HMDB00001 | 170.092 | 7.72 | -0.682 | -0.682 | -0.682 | -0.682 | -0.682 | -0.682 | -0.682 | -0.682 | -0.682 | -0.682 | 1.303 | 1.349 | 1.503 | 1.222 | 1.442 |
| 62 | A_0102 | ThPP | C00068 | HMDB01372 | 423.029 | 7.88 | -0.681 | -0.681 | -0.681 | -0.681 | -0.681 | -0.681 | -0.681 | -0.681 | -0.681 | -0.681 | 1.208 | 1.568 | 1.497 | 1.250 | 1.283 |
| 63 | A_0125 | CMP-*N*-acetylneuraminate | C00128 | HMDB01176 | 613.140 | 8.09 | -0.682 | -0.682 | -0.682 | -0.682 | -0.682 | -0.682 | -0.682 | -0.682 | -0.682 | -0.682 | 1.251 | 1.518 | 1.349 | 1.450 | 1.249 |
| 64 | C_0126 | Pyridoxamine-P | C00647 | HMDB01555 | 249.063 | 10.80 | -0.682 | -0.682 | -0.682 | -0.682 | -0.682 | -0.682 | -0.682 | -0.682 | -0.682 | -0.682 | 1.313 | 1.510 | 1.389 | 1.419 | 1.186 |
| 65 | A_0096 | FAD_divalent | C00016 | HMDB01248 | 391.571 | 7.99 | -0.669 | -0.620 | -0.760 | -0.706 | -0.733 | -0.653 | -0.634 | -0.633 | -0.688 | -0.702 | 1.413 | 1.578 | 1.340 | 1.366 | 1.101 |
| 66 | C_0037 | Taurine | C00245 | HMDB00251 | 126.022 | 22.72 | -0.613 | -0.691 | -0.671 | -0.846 | -0.733 | -0.611 | -0.585 | -0.620 | -0.709 | -0.713 | 1.282 | 1.498 | 1.545 | 1.322 | 1.144 |
| 67 | C_0049 | Gly-Gly | C02037 | HMDB11733 | 133.060 | 8.63 | -0.678 | -0.678 | -0.678 | -0.678 | -0.678 | -0.678 | -0.678 | -0.678 | -0.678 | -0.678 | 1.126 | 1.327 | 1.679 | 1.497 | 1.147 |
| 68 | A_0038 | Glycerol 3-phosphate | C00093 | HMDB00126 | 171.006 | 12.63 | -0.794 | -0.712 | -0.727 | -0.793 | -0.629 | -0.619 | -0.491 | -0.622 | -0.711 | -0.631 | 1.151 | 1.407 | 1.831 | 1.212 | 1.127 |
| 69 | A_0090 | AMP | C00020 | HMDB00045 | 346.055 | 9.51 | -0.665 | -0.684 | -0.660 | -0.668 | -0.648 | -0.657 | -0.686 | -0.680 | -0.618 | -0.681 | 0.860 | 1.773 | 1.799 | 1.151 | 1.063 |
| 70 | A_0021 | Threonic acid | C01620 | HMDB00943 | 135.031 | 9.20 | -0.675 | -0.675 | -0.675 | -0.675 | -0.675 | -0.675 | -0.675 | -0.675 | -0.675 | -0.675 | 1.098 | 1.661 | 1.619 | 1.360 | 1.008 |
| 71 | C_0130 | Glycerophosphocholine | C00670 | HMDB00086 | 258.109 | 22.32 | -0.834 | -0.838 | -0.819 | -0.837 | -0.837 | -0.484 | -0.521 | -0.480 | -0.357 | -0.582 | 1.368 | 1.156 | 1.874 | 1.416 | 0.776 |
| 72 | A_0031 | Terephthalic acid | C06337 | HMDB02428 | 165.018 | 17.56 | -0.651 | -0.614 | -0.640 | -0.689 | -0.699 | -0.636 | -0.742 | -0.667 | -0.645 | -0.673 | 1.355 | 0.939 | 1.944 | 1.470 | 0.947 |
| 73 | C_0007 | Morpholine | C14452 | HMDB31581 | 88.076 | 6.82 | -0.666 | -0.666 | -0.666 | -0.666 | -0.666 | -0.666 | -0.666 | -0.666 | -0.666 | -0.666 | 1.459 | 0.996 | 1.922 | 1.408 | 0.875 |
| 74 | C_0100 | *N*-Acetyllysine | C12989 | HMDB00446 | 189.123 | 10.18 | -0.675 | -0.675 | -0.675 | -0.675 | -0.675 | -0.675 | -0.675 | -0.675 | -0.675 | -0.675 | 1.764 | 1.136 | 1.512 | 1.297 | 1.039 |
| 75 | C_0024 | Creatinine | C00791 | HMDB00562 | 114.066 | 7.51 | -0.720 | -0.694 | -0.597 | -0.976 | -0.632 | -0.459 | -0.569 | -0.646 | -0.704 | -0.669 | 1.722 | 0.779 | 1.542 | 1.260 | 1.363 |
| 76 | C_0001 | Urea | C00086 | HMDB00294 | 61.040 | 21.79 | -0.789 | -0.795 | -0.532 | -0.915 | -0.630 | -0.468 | -0.542 | -0.522 | -0.697 | -0.750 | 1.327 | 0.787 | 1.801 | 1.189 | 1.536 |
| 77 | C_0105 | SDMA | No ID | HMDB03334 | 203.150 | 8.02 | -1.401 | -0.518 | -0.601 | -0.811 | -0.838 | -0.439 | -0.377 | -0.424 | -0.522 | -0.628 | 1.510 | 0.972 | 1.279 | 1.185 | 1.613 |
| 78 | C_0091 | Arg | C00062,C0079 | HMDB00517,H | 175.119 | 7.32 | -1.148 | -0.911 | -0.837 | -0.957 | -0.938 | -0.490 | -0.268 | -0.043 | -0.206 | -0.437 | 0.700 | 0.802 | 1.951 | 1.361 | 1.421 |
| 79 | C_0050 | Asn | C00152,C0190 | HMDB00168 | 133.061 | 10.78 | -0.906 | -0.719 | -0.747 | -0.903 | -0.842 | -0.555 | -0.358 | -0.447 | -0.325 | -0.672 | 0.786 | 1.234 | 2.131 | 1.208 | 1.116 |
| 80 | A_0004 | Lactic acid | C00186,C0025 | HMDB00190,H | 89.024 | 11.18 | -0.706 | -0.824 | -0.757 | -0.820 | -0.770 | -0.489 | -0.378 | -0.441 | -0.654 | -0.696 | 0.493 | 1.357 | 1.628 | 1.312 | 1.744 |
| 81 | A_0005 | 3-Hydroxybutyric acid | C01089,C0319 | HMDB00011,H | 103.040 | 9.78 | -0.739 | -0.812 | -0.721 | -0.798 | -0.727 | -0.538 | -0.592 | -0.555 | -0.534 | -0.644 | 0.906 | 1.312 | 1.909 | 1.083 | 1.450 |
| 82 | C_0083 | Taurocyamine | C01959 | HMDB03584 | 168.042 | 22.99 | -0.661 | -0.661 | -0.661 | -0.661 | -0.661 | -0.661 | -0.661 | -0.661 | -0.661 | -0.661 | 0.976 | 1.040 | 2.129 | 1.087 | 1.374 |
| 83 | A_0020 | Malic acid | C00149,C0049 | HMDB00156,H | 133.014 | 23.17 | -0.679 | -0.629 | -0.660 | -0.665 | -0.701 | -0.669 | -0.624 | -0.630 | -0.680 | -0.684 | 1.036 | 1.167 | 2.065 | 0.901 | 1.451 |
| 84 | A_0008 | Fumaric acid | C00122 | HMDB00134 | 115.003 | 27.83 | -0.679 | -0.649 | -0.675 | -0.655 | -0.680 | -0.659 | -0.630 | -0.628 | -0.676 | -0.678 | 0.979 | 1.172 | 1.994 | 0.856 | 1.609 |
| 85 | C_0010 | Ala | C00041,C0013 | HMDB00161,H | 90.055 | 9.28 | -0.674 | -0.654 | -0.627 | -0.699 | -0.697 | -0.638 | -0.600 | -0.616 | -0.670 | -0.686 | 0.883 | 1.254 | 2.013 | 0.772 | 1.639 |
| 86 | C_0079 | Carnitine | C00318,C0048 | HMDB00062 | 162.112 | 8.75 | -0.693 | -0.632 | -0.621 | -0.701 | -0.617 | -0.628 | -0.559 | -0.637 | -0.661 | -0.628 | 0.633 | 1.489 | 2.196 | 0.607 | 1.452 |
| 87 | C_0139 | Argininosuccinic acid | C03406 | HMDB00052 | 291.130 | 9.72 | -0.652 | -0.652 | -0.652 | -0.652 | -0.652 | -0.652 | -0.652 | -0.652 | -0.652 | -0.652 | 1.039 | 0.741 | 1.965 | 0.925 | 1.846 |
| 88 | C_0107 | *O*-Acetylcarnitine | C02571 | HMDB00201 | 204.122 | 9.28 | -0.722 | -0.607 | -0.624 | -0.697 | -0.646 | -0.659 | -0.620 | -0.693 | -0.676 | -0.643 | 0.958 | 0.677 | 1.795 | 1.395 | 1.762 |
| 89 | A_0106 | GDP | C00035 | HMDB01201 | 442.016 | 11.00 | 0.038 | -0.645 | 0.181 | -0.870 | -0.874 | -0.761 | -0.939 | -0.910 | -0.787 | -0.799 | 1.275 | 0.767 | 1.507 | 1.518 | 1.300 |
| 90 | A_0105 | ADP | C00008 | HMDB01341 | 426.021 | 11.23 | -0.417 | -0.521 | -0.404 | -0.650 | -0.661 | -0.720 | -0.759 | -0.743 | -0.720 | -0.723 | 0.534 | 0.715 | 1.122 | 2.003 | 1.943 |
| 91 | C_0125 | Malonylcarnitine | No ID | HMDB02095 | 248.111 | 10.54 | -0.642 | -0.642 | -0.642 | -0.642 | -0.642 | -0.642 | -0.642 | -0.642 | -0.642 | -0.642 | 0.524 | 0.775 | 1.344 | 2.039 | 1.742 |
| 92 | C_0137 | Octanoylcarnitine | C02838 | HMDB00791 | 288.215 | 10.73 | -0.601 | -0.601 | -0.601 | -0.601 | -0.601 | -0.601 | -0.601 | -0.601 | -0.601 | -0.601 | 0.769 | 0.113 | 1.260 | 2.527 | 1.344 |
| 93 | C_0117 | Butyrylcarnitine | C02862 | HMDB02013 | 232.153 | 9.85 | -0.694 | -0.694 | -0.694 | -0.656 | -0.694 | -0.643 | -0.516 | -0.561 | -0.694 | -0.572 | 1.021 | 0.813 | 0.994 | 2.387 | 1.205 |
| 94 | A_0027 | 2-Hydroxyglutaric acid | C02630,C0108 | HMDB00606,H | 147.029 | 16.98 | -0.656 | -0.656 | -0.656 | -0.656 | -0.656 | -0.656 | -0.656 | -0.656 | -0.656 | -0.656 | 1.090 | 0.733 | 1.407 | 2.139 | 1.191 |
| 95 | A_0036 | Dihydroxyacetone phosphate | C00111 | HMDB01473 | 168.990 | 13.23 | -0.600 | -0.508 | -0.639 | -0.603 | -0.684 | -0.626 | -0.651 | -0.639 | -0.679 | -0.702 | 1.533 | 0.288 | 1.519 | 2.108 | 0.885 |
| 96 | A_0120 | UDP-glucose<br>UDP-galactose | C00029<br>C00052 | HMDB00286<br>HMDB00302 | 565.048 | 8.83 | -0.727 | -0.453 | -0.451 | -0.932 | -0.813 | -0.270 | -0.471 | -0.845 | -0.572 | -0.851 | 1.285 | 0.661 | 1.527 | 2.097 | 0.813 |
| 97 | C_0028 | Betaine | C00719 | HMDB00043 | 118.086 | 11.59 | -0.745 | -0.577 | -0.651 | -0.710 | -0.751 | -0.598 | -0.376 | -0.597 | -0.577 | -0.721 | 0.860 | 0.904 | 2.112 | 1.902 | 0.523 |
| 98 | C_0063 | Stachydrine | C10172 | HMDB04827 | 144.102 | 11.84 | -0.771 | -0.476 | -0.755 | -0.805 | -0.843 | -0.572 | -0.089 | -0.683 | -0.632 | -0.768 | 1.104 | 1.613 | 1.594 | 1.722 | 0.361 |
| 99 | C_0110 | Kynurenine | C00328,C0171 | HMDB00684 | 209.091 | 10.17 | -0.766 | -0.785 | -0.553 | -0.660 | -0.643 | -0.615 | -0.421 | -0.394 | -0.709 | -0.714 | 2.031 | 0.900 | 1.689 | 1.446 | 0.194 |
| 100 | A_0089 | cGMP | C00942 | HMDB01314 | 344.039 | 7.51 | -0.577 | -0.577 | -0.577 | -0.577 | -0.577 | -0.577 | -0.577 | -0.577 | -0.577 | -0.577 | 1.414 | 1.500 | 2.035 | 1.396 | -0.577 |
| 101 | A_0071 | Glucose 1-phosphate | C00103 | HMDB01586 | 259.021 | 10.45 | -0.310 | -0.498 | -0.328 | -0.769 | -0.787 | -0.665 | -0.638 | -0.741 | -0.490 | -0.682 | 1.165 | 0.619 | 2.528 | 1.442 | 0.155 |
| 102 | C_0075 | His | C00135,C0076 | HMDB00177 | 156.077 | 7.51 | -0.702 | -0.657 | -0.608 | -0.699 | -0.691 | -0.566 | -0.495 | -0.501 | -0.485 | -0.603 | 0.756 | 0.836 | 2.739 | 1.109 | 0.566 |
| 103 | A_0109 | FMN | C00061 | HMDB01520 | 455.099 | 8.26 | -0.639 | -0.639 | -0.639 | -0.639 | -0.639 | -0.639 | -0.639 | -0.639 | -0.639 | -0.639 | 1.306 | 1.333 | 2.311 | 0.872 | 0.573 |
| 104 | A_0110 | Adenylosuccinic acid | C03794 | HMDB00536 | 462.066 | 14.40 | -0.646 | -0.633 | -0.633 | -0.637 | -0.638 | -0.598 | -0.635 | -0.646 | -0.616 | -0.622 | 1.641 | 1.028 | 2.271 | 0.376 | 0.988 |
| 105 | C_0111 | XC0061 | - | - | 218.138 | 9.58 | -0.619 | -0.619 | -0.619 | -0.619 | -0.520 | -0.619 | -0.517 | -0.551 | -0.619 | -0.523 | 0.213 | 0.983 | 2.622 | 0.414 | 1.591 |
| 106 | C_0054 | Adenine | C00147 | HMDB00034 | 136.062 | 7.84 | -0.672 | -0.405 | -0.811 | -0.305 | -0.950 | -0.572 | -0.187 | -0.410 | -0.543 | -0.845 | 0.037 | 1.787 | 2.324 | 0.331 | 1.219 |
| 107 | A_0073 | *myo*-Inositol 2-phosphate | No ID | No ID | 259.021 | 10.84 | -1.259 | -0.591 | -0.844 | -0.515 | -0.736 | -0.506 | -0.174 | -0.187 | -0.681 | -0.736 | 0.433 | 2.006 | 1.648 | 1.055 | 1.087 |

| | | | | | | | | | | | | | | | | | | | | | |
|---|---|---|---|---|---|---|---|---|---|---|---|---|---|---|---|---|---|---|---|---|---|
| 108 | C_0148 | *S*-Adenosylmethionine | C00019 | HMDB01185 | 399.144 | 7.32 | -0.834 | -0.387 | -0.410 | -0.843 | -0.900 | -0.534 | -0.439 | -0.587 | -0.598 | -0.813 | 0.613 | 1.906 | 1.978 | 0.938 | 0.908 |
| 109 | C_0127 | γ-Glu-Cys | C00669 | HMDB01049 | 251.069 | 13.23 | -0.644 | -0.644 | -0.644 | -0.644 | -0.644 | -0.644 | -0.644 | -0.644 | -0.644 | -0.644 | 0.683 | 1.835 | 2.071 | 0.965 | 0.889 |
| 110 | A_0012 | Succinic acid | C00042 | HMDB00254 | 117.019 | 22.74 | -0.610 | -0.587 | -0.602 | -0.620 | -0.629 | -0.619 | -0.576 | -0.597 | -0.621 | -0.608 | 0.163 | 1.584 | 2.422 | 1.183 | 0.716 |
| 111 | C_0090 | $N^5$-Ethylglutamine | C01047 | No ID | 175.109 | 11.70 | -0.914 | -0.914 | -0.358 | -0.914 | -0.914 | -0.300 | -0.096 | -0.914 | -0.304 | -0.452 | 0.775 | 1.659 | 2.164 | 1.025 | 0.460 |
| 112 | C_0070 | Glu | C00025,C0021 | HMDB00148,H | 148.060 | 11.24 | -0.752 | -0.467 | -0.726 | -0.896 | -0.682 | -0.375 | -0.047 | -0.419 | -0.674 | -0.738 | 1.242 | 2.568 | 1.316 | 0.428 | 0.222 |
| 113 | C_0142 | Glutathione (GSH) | C00051 | HMDB00125 | 308.090 | 13.60 | -0.169 | -0.247 | -0.367 | -0.560 | -0.711 | -0.920 | -0.851 | -0.888 | -0.899 | -0.890 | 1.163 | 1.931 | 0.969 | 1.262 | 1.176 |
| 114 | C_0069 | *threo*-β-Methylaspartic acid | C03618 | No ID | 148.060 | 12.73 | -0.635 | -0.635 | -0.635 | -0.635 | -0.635 | -0.635 | -0.635 | -0.635 | -0.635 | -0.635 | 1.544 | 2.296 | 0.458 | 1.017 | 1.036 |
| 115 | C_0140 | 5'-Deoxy-5'-methylthioadenosine | C00170 | HMDB01173 | 298.097 | 10.41 | -0.637 | -0.637 | -0.637 | -0.637 | -0.637 | -0.637 | -0.637 | -0.637 | -0.637 | -0.637 | 0.770 | 2.444 | 0.869 | 1.217 | 1.069 |
| 116 | A_0094 | CoA_divalent | C00010 | HMDB01423 | 382.549 | 10.96 | -0.585 | -0.597 | -0.574 | -0.644 | -0.651 | -0.634 | -0.676 | -0.653 | -0.637 | -0.633 | 0.674 | 2.533 | 1.084 | 0.926 | 1.068 |
| 117 | A_0074 | Fructose 6-phosphate | C05345,C0008 | HMDB00124 | 259.021 | 10.12 | -0.638 | -0.638 | -0.638 | -0.585 | -0.601 | -0.638 | -0.584 | -0.592 | -0.638 | -0.638 | 0.431 | 2.540 | 1.154 | 0.865 | 1.202 |
| 118 | A_0072 | Glucose 6-phosphate | C00668,C0117 | HMDB01401 | 259.021 | 10.12 | -0.610 | -0.476 | -0.527 | -0.658 | -0.697 | -0.680 | -0.681 | -0.694 | -0.688 | -0.702 | 0.771 | 2.348 | 1.329 | 0.871 | 1.095 |
| 119 | A_0088 | Fructose 1,6-diphosphate | C00354 | HMDB01058 | 338.988 | 15.37 | -0.648 | -0.626 | -0.604 | -0.674 | -0.690 | -0.683 | -0.682 | -0.686 | -0.668 | -0.684 | 0.838 | 2.030 | 1.258 | 1.286 | 1.231 |
| 120 | C_0114 | Carnosine | C00386 | HMDB00033 | 227.113 | 6.95 | -0.675 | -0.650 | -0.581 | -0.659 | -0.664 | -0.675 | -0.665 | -0.657 | -0.676 | -0.681 | 0.756 | 2.010 | 0.919 | 1.409 | 1.488 |
| 121 | C_0045 | 3-Guanidinopropionic acid | C03065 | No ID | 132.076 | 8.25 | -0.625 | -0.625 | -0.625 | -0.625 | -0.625 | -0.625 | -0.625 | -0.625 | -0.625 | -0.625 | 0.879 | 2.393 | 0.524 | 0.835 | 1.619 |
| 122 | C_0146 | *S*-Lactoylglutathione | C03451 | HMDB01066 | 380.112 | 14.24 | -0.565 | -0.565 | -0.565 | -0.565 | -0.565 | -0.565 | -0.565 | -0.565 | -0.565 | -0.565 | 0.268 | 2.299 | 0.235 | 0.605 | 2.242 |
| 123 | C_0008 | Putrescine | C00134 | HMDB01414 | 89.107 | 4.85 | -1.112 | -0.637 | -0.939 | -0.960 | -0.928 | -0.270 | 0.327 | -0.204 | -0.253 | -0.762 | 1.160 | 1.611 | 0.101 | 0.813 | 2.053 |
| 124 | C_0096 | Phosphorylcholine | C00588 | HMDB01565 | 184.072 | 21.17 | -0.795 | -0.210 | -0.695 | -1.035 | -0.788 | -0.422 | -0.215 | -0.674 | -0.603 | -0.862 | 1.332 | 1.506 | 0.400 | 1.041 | 2.019 |
| 125 | A_0068 | XA0033 | - | - | 242.079 | 7.76 | -0.728 | -0.260 | -0.656 | -1.007 | -0.741 | -0.486 | -0.374 | -0.672 | -0.666 | -0.880 | 1.361 | 1.483 | 0.574 | 1.126 | 1.927 |
| 126 | C_0141 | Glutathione (GSSG)_divalent | C00127 | HMDB03337 | 307.083 | 12.52 | -1.085 | -0.753 | -1.205 | -0.863 | -0.026 | -0.342 | 0.134 | -0.562 | -0.810 | -0.751 | 1.425 | 1.521 | 0.682 | 1.090 | 1.546 |
| 127 | C_0057 | Trigonelline | C01004 | HMDB00875 | 138.056 | 10.78 | -0.574 | -0.574 | -0.574 | -0.574 | -0.574 | -0.574 | -0.574 | -0.574 | -0.574 | -0.574 | 1.916 | 1.585 | -0.574 | 1.757 | 1.053 |
| 128 | C_0135 | Saccharopine | C00449 | HMDB00279 | 277.139 | 11.05 | -0.651 | -0.651 | -0.651 | -0.651 | -0.651 | -0.651 | -0.651 | -0.651 | -0.651 | -0.651 | 1.842 | 1.048 | 0.538 | 1.887 | 1.194 |
| 129 | C_0014 | *N*,*N*-Dimethylglycine | C01026 | HMDB00092 | 104.071 | 11.24 | -0.336 | -0.569 | -1.256 | -1.256 | -0.546 | 0.219 | -0.428 | -0.624 | -0.683 | -0.379 | 2.416 | 0.933 | 0.978 | 0.613 | 0.917 |
| 130 | A_0091 | IMP | C00130 | HMDB00175 | 347.039 | 9.80 | -0.545 | -0.613 | -0.500 | -0.616 | -0.595 | -0.622 | -0.637 | -0.637 | -0.623 | -0.628 | 2.763 | 0.772 | 0.709 | 0.721 | 1.053 |
| 131 | C_0022 | Histamine | C00388 | HMDB00870 | 112.086 | 4.91 | -0.643 | -0.457 | -0.302 | -0.565 | -0.732 | -0.677 | -0.508 | -0.652 | -0.706 | -0.717 | 2.531 | 1.188 | 0.784 | 0.102 | 1.354 |
| 132 | C_0053 | Asp | C00049,C0040 | HMDB00191,H | 134.045 | 11.88 | -0.895 | -0.931 | -0.740 | -0.284 | -0.815 | -0.542 | -0.414 | -0.549 | -0.217 | -0.629 | 1.605 | 1.461 | 1.732 | -0.223 | 1.441 |
| 133 | C_0013 | Homoserinelactone | No ID | No ID | 102.055 | 7.30 | -0.557 | -0.557 | -0.557 | -0.557 | -0.557 | -0.557 | -0.557 | -0.557 | -0.557 | -0.557 | 1.293 | 1.365 | 2.459 | -0.557 | 1.006 |
| 134 | A_0108 | Octanoyl CoA_divalent | C01944 | HMDB01070 | 445.602 | 9.53 | -0.481 | -0.481 | -0.481 | -0.481 | -0.481 | -0.481 | -0.481 | -0.481 | -0.481 | -0.481 | 1.930 | -0.481 | 2.156 | -0.481 | 1.688 |
| 135 | A_0100 | Acetyl CoA_divalent | C00024 | HMDB01206 | 403.554 | 10.56 | -0.313 | -0.361 | -0.380 | -0.620 | -0.591 | -0.386 | -0.620 | -0.587 | -0.350 | -0.386 | 2.016 | -0.519 | 2.643 | 0.629 | -0.174 |
| 136 | A_0070 | Glucosamine 6-phosphate | C00352 | HMDB01254 | 258.038 | 8.85 | -0.535 | -0.535 | -0.535 | -0.535 | -0.535 | -0.535 | -0.535 | -0.535 | -0.535 | -0.535 | 1.490 | -0.535 | 2.665 | 0.963 | 0.772 |
| 137 | C_0094 | Glucosamine | C00329 | HMDB01514 | 180.087 | 9.56 | -0.720 | -0.737 | -0.686 | -0.611 | -0.304 | -0.639 | -0.076 | -0.263 | -0.681 | -0.656 | 1.240 | -0.617 | 2.013 | 2.043 | 0.693 |
| 138 | A_0024 | XA0004 | - | - | 144.029 | 9.29 | -0.546 | -0.546 | -0.546 | -0.546 | -0.546 | -0.546 | -0.546 | -0.546 | -0.546 | -0.546 | 0.675 | -0.546 | 2.433 | 1.174 | 1.723 |
| 139 | A_0118 | GTP | C00044 | HMDB01273 | 521.983 | 11.86 | -0.528 | -0.514 | -0.549 | -0.570 | -0.555 | -0.665 | -0.667 | -0.654 | -0.670 | -0.676 | 0.918 | -0.025 | 1.298 | 1.518 | 2.339 |
| 140 | C_0051 | Ornithine | C00077,C0051 | HMDB00214,H | 133.097 | 7.01 | -0.811 | -0.708 | -0.761 | -0.852 | -0.678 | -0.440 | -0.503 | -0.306 | -0.543 | -0.267 | 0.436 | 0.374 | 1.654 | 0.898 | 2.509 |
| 141 | C_0064 | Acetylcholine | C01996 | HMDB00895 | 146.117 | 7.84 | -0.377 | -0.377 | -0.377 | -0.377 | -0.377 | -0.377 | -0.377 | -0.377 | -0.377 | -0.377 | -0.377 | -0.377 | 2.724 | -0.377 | 2.176 |
| 142 | C_0081 | Methionine sulfoxide | C02989 | HMDB02005 | 166.052 | 12.28 | -0.379 | -0.379 | -0.379 | -0.379 | -0.379 | -0.379 | -0.379 | -0.379 | -0.379 | -0.379 | -0.379 | -0.379 | 2.455 | -0.379 | 2.471 |
| 143 | A_0049 | *N*-Acetylglutamine | No ID | HMDB06029 | 187.073 | 8.02 | -0.378 | -0.378 | -0.378 | -0.378 | -0.378 | -0.378 | -0.378 | -0.378 | -0.378 | -0.378 | -0.378 | -0.378 | 2.298 | -0.378 | 2.619 |
| 144 | C_0041 | Pipecolic acid | C00408 | HMDB00070,H | 130.086 | 10.58 | -0.670 | -0.926 | -0.926 | -0.926 | 0.226 | -0.426 | -0.391 | -0.239 | -0.315 | 0.670 | -0.080 | 0.114 | 0.696 | 0.114 | 3.078 |
| 145 | C_0026 | Guanidoacetic acid | C00581 | HMDB00128 | 118.061 | 8.47 | -0.546 | -0.533 | -0.509 | -0.521 | -0.467 | -0.415 | -0.356 | -0.376 | -0.315 | -0.394 | 0.414 | 0.071 | 0.212 | 0.323 | 3.413 |
| 146 | C_0113 | Cystathionine | C00542,C0229 | HMDB00099 | 223.075 | 10.19 | -1.140 | -0.511 | -0.771 | 0.141 | 0.006 | -0.493 | -0.242 | -0.894 | -0.612 | -0.240 | 0.390 | -0.022 | 0.763 | 0.590 | 3.035 |
| 147 | A_0117 | ATP | C00002 | HMDB00538 | 505.988 | 12.19 | -0.535 | -0.383 | -0.546 | -0.384 | -0.342 | -0.566 | -0.467 | -0.551 | -0.580 | -0.574 | 0.097 | -0.291 | 0.420 | 2.060 | 2.642 |
| 148 | C_0004 | Methylguanidine | C02294 | HMDB01522 | 74.071 | 6.36 | -0.379 | -0.379 | -0.379 | -0.379 | -0.379 | -0.379 | -0.379 | -0.379 | -0.379 | -0.379 | -0.379 | -0.379 | -0.379 | 2.368 | 2.555 |
| 149 | A_0114 | UTP | C00075 | HMDB00285 | 482.959 | 13.10 | -0.189 | -0.294 | -0.190 | -0.598 | -0.288 | -0.371 | -0.419 | -0.503 | -0.598 | -0.434 | 0.286 | -0.598 | -0.598 | 2.461 | 2.332 |
| 150 | C_0098 | $N^6$-Acetyllysine | C02727 | HMDB00206 | 189.123 | 11.81 | -0.258 | -0.258 | -0.258 | -0.258 | -0.258 | -0.258 | -0.258 | -0.258 | -0.258 | -0.258 | -0.258 | -0.258 | -0.258 | 3.615 | -0.258 |
| 151 | A_0037 | Glyceraldehyde 3-phosphate | C00118,C0066 | HMDB01112 | 168.989 | 11.63 | -0.258 | -0.258 | -0.258 | -0.258 | -0.258 | -0.258 | -0.258 | -0.258 | -0.258 | -0.258 | -0.258 | -0.258 | -0.258 | 3.615 | -0.258 |
| 152 | A_0065 | XA0027 | - | - | 227.200 | 7.36 | -0.374 | -0.374 | -0.374 | -0.374 | -0.374 | -0.374 | -0.374 | -0.374 | -0.374 | -0.374 | -0.374 | 1.998 | -0.374 | 2.862 | -0.374 |
| 153 | A_0030 | 8-Hydroxyoctanoic acid | No ID | No ID | 159.101 | 8.04 | -0.481 | -0.481 | -0.481 | -0.481 | -0.481 | -0.481 | -0.481 | -0.481 | -0.481 | -0.481 | -0.481 | 1.827 | -0.481 | 2.213 | 1.729 |
| 154 | A_0045 | Hippuric acid | C01586 | HMDB00714 | 178.050 | 8.34 | -0.432 | -0.432 | -0.432 | -0.432 | -0.432 | -0.432 | -0.432 | -0.432 | -0.432 | -0.432 | -0.432 | 3.051 | -0.432 | 1.369 | 0.761 |
| 155 | C_0020 | Hypotaurine | C00519 | HMDB00965 | 110.027 | 18.66 | -0.362 | -0.463 | -0.652 | -0.603 | -0.639 | -0.351 | 0.008 | -0.615 | -0.434 | -0.685 | 0.506 | 3.234 | -0.019 | 0.571 | 0.505 |
| 156 | C_0085 | Noradrenaline | C00547 | HMDB00216 | 170.081 | 9.27 | -0.251 | -0.575 | -0.575 | -0.366 | -0.575 | -0.253 | -0.245 | -0.212 | -0.084 | -0.313 | 0.252 | 3.259 | 1.089 | -0.575 | -0.575 |
| 157 | C_0116 | Ergothioneine | C05570 | HMDB03045 | 230.095 | 18.08 | -0.258 | -0.258 | -0.258 | -0.258 | -0.258 | -0.258 | -0.258 | -0.258 | -0.258 | -0.258 | -0.258 | 3.615 | -0.258 | -0.258 | -0.258 |
| 158 | A_0127 | 3'-Dephospho CoA | C00882 | HMDB01373 | 686.140 | 7.93 | -0.258 | -0.258 | -0.258 | -0.258 | -0.258 | -0.258 | -0.258 | -0.258 | -0.258 | -0.258 | -0.258 | 3.615 | -0.258 | -0.258 | -0.258 |
| 159 | A_0019 | 6-Hydroxyhexanoic acid | C06103 | No ID | 131.069 | 8.56 | -0.258 | -0.258 | -0.258 | -0.258 | -0.258 | -0.258 | -0.258 | -0.258 | -0.258 | -0.258 | -0.258 | 3.615 | -0.258 | -0.258 | -0.258 |
| 160 | C_0006 | Trimethylamine *N*-oxide | C01104 | HMDB00925 | 76.076 | 6.79 | -0.682 | 0.530 | -0.250 | -0.606 | -0.439 | -0.474 | 0.498 | -0.065 | -0.243 | -0.977 | -0.514 | 3.291 | 0.109 | -0.193 | 0.014 |
| 161 | A_0063 | Pantothenic acid | C00864 | HMDB00210 | 218.103 | 7.78 | 0.092 | 1.319 | -0.376 | -0.917 | -0.574 | -0.383 | 0.708 | -0.671 | -0.855 | -0.973 | 0.414 | 2.764 | -0.556 | -0.138 | 0.146 |
| 162 | A_0107 | XA0065 | - | - | 445.052 | 7.03 | 0.113 | 0.918 | 1.314 | -1.131 | -0.655 | -0.412 | -0.256 | -0.572 | -1.059 | -1.408 | -0.487 | 2.205 | 0.794 | 0.083 | 0.553 |
| 163 | A_0061 | 3-Indoxylsulfuric acid | No ID | HMDB00682 | 212.003 | 9.75 | -0.792 | -0.820 | 0.142 | -0.650 | -0.745 | -0.486 | -0.788 | 1.097 | -0.196 | -0.841 | 0.845 | 1.851 | -1.188 | 1.147 | 1.424 |
| 164 | C_0104 | ADMA | C03626 | HMDB01539 | 203.149 | 7.89 | -0.546 | -0.472 | -0.652 | -0.816 | -0.831 | 0.161 | 0.796 | 0.554 | -0.054 | -0.039 | 0.618 | 2.571 | -1.880 | 0.179 | 0.410 |
| 165 | C_0030 | Homoserine | C00263 | HMDB00719 | 120.066 | 10.37 | -0.966 | -0.966 | -0.966 | -0.966 | -0.966 | 0.293 | 0.615 | 1.783 | 0.706 | 1.159 | 0.262 | 1.058 | -0.966 | -0.966 | 0.889 |
| 166 | C_0095 | Tyr | C00082,C0153 | HMDB00158 | 182.081 | 11.68 | -1.775 | -1.170 | -1.465 | -0.752 | -1.064 | 0.345 | 1.474 | 0.521 | 0.653 | 0.099 | 0.085 | 0.926 | 0.684 | 0.352 | 1.087 |
| 167 | C_0025 | Pro | C00148,C0076 | HMDB00162,H | 116.070 | 11.11 | -1.574 | -1.076 | -1.340 | -1.512 | -0.918 | 0.648 | 1.030 | 0.368 | 0.370 | 0.237 | 0.434 | 0.398 | 1.026 | 0.505 | 1.405 |
| 168 | C_0048 | Leu | C00123,C0157 | HMDB00687 | 132.102 | 10.61 | -1.430 | -1.090 | -0.987 | -1.171 | -0.893 | 0.679 | 1.636 | 0.900 | -0.049 | -0.143 | -0.079 | 0.478 | 1.507 | -0.365 | 1.007 |
| 169 | C_0027 | Val | C00183,C0641 | HMDB00883 | 118.086 | 10.31 | -1.548 | -1.171 | -1.042 | -1.503 | -0.668 | 0.693 | 0.960 | 0.664 | 0.110 | 0.162 | 0.206 | 0.590 | 1.598 | -0.289 | 1.238 |
| 170 | C_0068 | Lys | C00047,C0073 | HMDB00182,H | 147.113 | 7.08 | -1.372 | -1.048 | -1.094 | -1.390 | -0.954 | 0.282 | 0.687 | 0.780 | 1.312 | 0.755 | 0.017 | 0.209 | 1.442 | -0.629 | 1.004 |
| 171 | C_0015 | 2-Aminoisobutyric acid | C03665 | HMDB01906 | 104.071 | 9.91 | -1.190 | -0.926 | -1.105 | -1.547 | -0.593 | 0.358 | 0.097 | 0.127 | 2.207 | 0.312 | -0.038 | -0.045 | 0.903 | 0.095 | 1.345 |
| 172 | C_0129 | Dyphylline | C07819 | No ID | 255.107 | 22.79 | -1.463 | -1.044 | -0.263 | -1.702 | -0.731 | 0.364 | -0.407 | 0.738 | 0.847 | 1.518 | -0.193 | -0.082 | 1.707 | 0.668 | 0.042 |
| 173 | A_0121 | UDP-glucuronic acid | C00167 | HMDB00935 | 579.027 | 11.40 | -0.741 | -0.276 | -0.691 | -1.624 | -1.467 | 1.731 | 0.005 | -1.207 | 1.076 | 0.180 | 0.448 | 0.524 | 0.081 | 1.151 | 0.810 |
| 174 | C_0003 | XC0001 | - | - | 72.081 | 6.56 | -0.479 | -0.546 | -1.275 | -0.677 | -1.275 | 1.239 | -0.016 | -0.327 | 0.482 | -0.810 | 2.578 | 0.729 | 0.167 | 0.147 | 0.063 |
| 175 | C_0034 | 2-Amino-2-(hydroxymethyl)-1,3-propanediol | C07182 | No ID | 122.081 | 8.54 | 2.973 | -0.387 | -0.387 | -0.160 | -0.387 | 1.828 | -0.387 | -0.387 | -0.387 | -0.387 | -0.387 | -0.387 | -0.387 | -0.387 | -0.387 |
| 176 | C_0059 | Urocanic acid | C00785 | HMDB00301 | 139.049 | 8.51 | 1.561 | 1.727 | -0.627 | 0.441 | -0.627 | 2.050 | -0.627 | 0.495 | -0.627 | -0.627 | -0.627 | -0.627 | -0.627 | -0.627 | -0.627 |
| 177 | A_0077 | 6-Phosphogluconic acid | C00345 | HMDB01316 | 275.016 | 15.49 | 0.333 | 3.008 | -0.363 | 0.175 | -0.556 | 1.091 | 0.643 | -0.342 | -0.062 | -0.737 | -0.628 | -0.611 | -0.523 | -0.852 | -0.576 |
| 178 | C_0035 | 2-Phenylethylamine | C05332 | HMDB12275 | 122.096 | 8.05 | 0.571 | 1.936 | 0.339 | -0.559 | -0.206 | 1.264 | -0.175 | -0.239 | -0.633 | -1.257 | 0.644 | -2.055 | 0.938 | -0.522 | -0.045 |
| 179 | A_0026 | Adipic acid | C06104 | HMDB00448 | 145.049 | 16.13 | 1.170 | 1.983 | 0.854 | 0.391 | 0.402 | 0.732 | 0.560 | 0.615 | -0.958 | -0.958 | -0.958 | -0.958 | -0.958 | -0.958 | -0.958 |
| 180 | C_0046 | Creatine | C00300 | HMDB00064 | 132.410 | 9.06 | 0.828 | 1.536 | 1.757 | -0.057 | 1.186 | -0.053 | 0.874 | 0.010 | -0.511 | -0.540 | -1.079 | -1.116 | -1.076 | -0.985 | -0.774 |
| 181 | A_0043 | *N*-Acetylaspartic acid | C01042 | HMDB00812 | 174.040 | 15.37 | 1.363 | 1.208 | 1.646 | 1.065 | 0.831 | -0.095 | -0.383 | -0.496 | 0.312 | -0.339 | -0.581 | -1.196 | -1.089 | -0.953 | -1.293 |
| 182 | C_0012 | Glycerol | C00116 | HMDB00131 | 93.055 | 22.74 | 1.900 | 1.623 | 0.942 | 0.689 | -0.227 | 0.062 | -0.740 | 0.492 | -0.402 | 0.629 | -1.014 | -1.050 | -0.824 | -1.121 | -0.961 |
| 183 | A_0128 | NADP$^+$ | C00006 | HMDB00217 | 742.071 | 9.75 | 1.544 | 1.520 | 1.874 | -0.329 | -0.296 | 0.715 | -0.163 | -0.058 | -0.141 | 0.307 | -0.994 | -0.994 | -0.994 | -0.994 | -0.994 |
| 184 | A_0126 | NAD$^+$ | C00003 | HMDB00902 | 662.103 | 6.61 | 1.422 | 1.500 | 1.230 | -0.461 | 0.472 | 1.002 | 0.073 | -0.120 | 0.149 | 0.404 | -1.195 | -1.297 | -0.813 | -1.180 | -1.187 |
| 185 | A_0050 | Azelaic acid | C08261 | HMDB00784 | 187.097 | 12.91 | 1.108 | 1.951 | 0.749 | 0.622 | 0.054 | 0.298 | 0.235 | 0.417 | 0.391 | 0.284 | -1.222 | -1.222 | -1.222 | -1.222 | -1.222 |
| 186 | A_0042 | Suberic acid | C08278 | HMDB00893 | 173.081 | 13.70 | 0.698 | 2.006 | 0.252 | 0.395 | 0.257 | 0.762 | 0.438 | 0.813 | 0.307 | 0.242 | -1.234 | -1.234 | -1.234 | -1.234 | -1.234 |
| 187 | A_0104 | 3',5'-ADP | C00054 | HMDB00061 | 426.020 | 14.30 | 0.512 | 1.654 | 0.339 | 1.481 | 0.310 | 0.013 | 0.831 | 0.646 | 0.206 | 0.107 | -1.220 | -1.220 | -1.220 | -1.220 | -1.220 |
| 188 | A_0046 | 2-Phosphoglyceric acid | C00631 | HMDB03391 | 184.985 | 21.25 | 0.627 | 1.454 | 0.950 | 1.661 | 0.975 | -0.287 | 0.250 | 0.374 | -0.202 | -0.082 | -1.144 | -1.144 | -1.144 | -1.144 | -1.144 |
| 189 | A_0047 | 3-Phosphoglyceric acid | C00197 | HMDB00807 | 184.985 | 21.29 | 0.485 | 1.451 | 0.705 | 1.981 | 0.908 | -0.427 | 0.158 | 0.247 | -0.316 | -0.339 | -0.916 | -1.573 | -0.092 | -1.026 | -1.248 |
| 190 | A_0034 | Phosphoenolpyruvic acid | C00074 | HMDB00263 | 166.974 | 23.63 | 0.017 | 1.004 | 0.039 | 2.530 | 1.685 | -0.426 | -0.177 | -0.117 | -0.324 | -0.108 | -0.825 | -0.825 | -0.825 | -0.825 | -0.825 |
| 191 | A_0029 | Pimelic acid | C02656 | HMDB00857 | 159.065 | 14.71 | -0.816 | 2.374 | -0.816 | 0.714 | 0.342 | 0.825 | 0.728 | 0.841 | -0.816 | 0.702 | -0.816 | -0.816 | -0.816 | -0.816 | -0.816 |
| 192 | A_0040 | Isovalerylalanine | No ID | HMDB00747 | 172.097 | 8.26 | -1.104 | 1.410 | -0.130 | 0.569 | 0.629 | 0.801 | 0.491 | 0.470 | 1.117 | 1.269 | -1.104 | -1.104 | -1.104 | -1.104 | -1.104 |
| 193 | A_0058 | Sebacic acid | C08277 | HMDB00792 | 201.112 | 12.27 | -1.064 | 2.150 | 0.569 | 0.579 | 0.137 | 0.504 | 0.725 | 0.861 | 0.505 | 0.357 | -1.064 | -1.064 | -1.064 | -1.064 | -1.064 |
| 194 | C_0133 | Inosine | C00294 | HMDB00195 | 269.087 | 20.11 | 0.352 | 1.643 | 0.999 | -0.168 | 0.409 | -0.285 | 1.529 | 0.542 | -0.653 | -0.334 | -1.647 | 0.341 | 0.008 | -1.653 | -1.083 |
| 195 | C_0019 | Diethanolamine | C06772 | HMDB04437 | 106.086 | 7.87 | -0.353 | 0.905 | 2.254 | 0.625 | 0.186 | -0.657 | -0.205 | 0.093 | 0.485 | -0.920 | -0.784 | 1.070 | -0.172 | -1.972 | -0.556 |
| 196 | A_0056 | Gluconic acid | C00257 | HMDB00625 | 195.050 | 8.35 | -0.189 | 0.277 | 2.896 | -0.308 | -0.712 | 0.243 | 0.287 | 0.053 | -0.156 | -0.209 | -1.099 | 0.091 | -1.099 | 1.027 | -1.099 |
| 197 | A_0022 | Ethanolamine phosphate | C00346 | HMDB00224 | 140.011 | 8.19 | -0.037 | 1.074 | 1.630 | -1.360 | -1.108 | 1.021 | 1.625 | 0.858 | 0.072 | -0.711 | -0.545 | -0.846 | -0.473 | -0.931 | -0.269 |
| 198 | C_0073 | Triethanolamine | C06771 | No ID | 150.112 | 8.48 | -0.165 | 2.622 | 0.048 | -0.724 | -0.523 | 0.700 | -1.211 | 0.068 | 1.380 | 0.371 | -1.120 | -0.248 | -0.928 | 0.065 | -0.335 |
| 199 | A_0015 | 4-Methyl-2-oxovaleric acid 3-Methyl-2-oxovaleric acid | C00233 C00671,C034 65 | HMDB00695 HMDB00491 | 129.055 | 9.95 | -0.889 | 0.871 | 0.932 | -0.889 | 1.066 | 0.973 | 0.597 | -0.889 | 1.473 | 1.200 | -0.889 | -0.889 | -0.889 | -0.889 | -0.889 |
| 200 | A_0060 | Phosphocreatine | C02305 | HMDB01511 | 210.028 | 12.83 | 1.241 | 0.478 | 2.290 | -0.808 | -0.799 | 0.211 | -0.521 | -0.435 | 0.875 | 1.244 | -0.785 | -0.808 | -0.808 | -0.747 | -0.630 |
| 201 | C_0033 | Cys | C00097,C0073 | HMDB00574,H | 122.027 | 11.60 | 0.899 | 0.845 | 1.944 | -0.514 | -0.571 | -0.205 | -0.328 | -0.590 | 1.885 | 0.784 | -0.830 | -0.830 | -0.830 | -0.830 | -0.830 |
| 202 | C_0124 | $N^1$-Acetylspermine | C02567 | HMDB01186 | 245.233 | 5.72 | 0.740 | 0.969 | 0.043 | -0.632 | -0.147 | -0.010 | -0.632 | -0.632 | 3.015 | 0.448 | -0.632 | -0.632 | -0.632 | -0.632 | -0.632 |
| 203 | C_0119 | Cystine | C00491,C0142 | HMDB00192 | 241.031 | 11.28 | -0.346 | -0.300 | -0.101 | -0.594 | -0.594 | 0.577 | -0.102 | 0.097 | 3.052 | 1.282 | -0.594 | -0.594 | -0.594 | -0.594 | -0.594 |
| 204 | C_0132 | Adenosine | C00212 | HMDB00050 | 268.104 | 10.14 | -0.525 | -0.674 | -0.687 | 1.469 | -0.650 | 1.948 | 0.043 | 0.275 | 1.941 | 0.335 | -0.797 | -0.507 | -0.764 | -0.682 | -0.725 |
| 205 | A_0001 | Glyoxylic acid | C00048 | HMDB00119 | 72.993 | 12.44 | -0.465 | -0.465 | -0.465 | -0.465 | -0.465 | 2.088 | 1.047 | -0.465 | 2.440 | -0.465 | -0.465 | -0.465 | -0.465 | -0.465 | -0.465 |
| 206 | C_0112 | β-Ala-Lys | C05341 | No ID | 218.149 | 7.00 | -0.989 | -0.989 | -0.989 | -0.989 | -0.989 | 2.105 | 0.950 | 0.637 | 1.314 | 0.780 | -0.989 | -0.012 | -0.110 | 0.221 | 0.047 |
| 207 | C_0043 | *N*-Acetylputrescine | C02714 | HMDB02064 | 131.117 | 8.77 | -0.109 | 0.133 | -0.761 | -0.761 | -0.382 | 2.169 | 1.936 | 0.051 | 0.487 | 1.040 | -0.761 | -0.761 | -0.761 | -0.761 | -0.761 |
| 208 | A_0053 | XA0019 | - | - | 191.019 | 8.47 | -0.616 | -0.514 | -0.621 | -0.455 | -0.327 | 1.574 | 2.512 | 1.065 | 0.687 | -0.014 | -0.717 | -0.392 | -0.801 | -0.672 | -0.709 |
| 209 | A_0059 | Mucic acid | C00879,C0180 | HMDB00639 | 209.029 | 15.14 | -0.778 | -0.429 | -0.778 | -0.116 | 0.203 | 1.028 | 2.519 | 1.175 | 0.822 | 0.247 | -0.778 | -0.778 | -0.778 | -0.778 | -0.778 |
| 210 | C_0084 | Tyr-Arg_divalent | No ID | No ID | 169.594 | 7.94 | -0.558 | -0.568 | -0.546 | -0.541 | -0.619 | 1.446 | 2.414 | 0.878 | 0.682 | 0.898 | -0.697 | -0.697 | -0.697 | -0.697 | -0.697 |
| 211 | C_0002 | Ethanolamine | C00189 | HMDB00149 | 62.060 | 6.53 | -0.859 | -0.372 | -0.709 | -0.904 | -0.462 | 1.775 | 2.177 | 1.002 | 0.660 | 0.434 | 0.028 | -0.889 | -0.365 | -0.753 | -0.762 |
| 212 | C_0031 | Betaine aldehyde_+$H_2O$ | C00576 | HMDB01252 | 120.102 | 7.67 | -0.651 | -0.651 | -0.651 | -0.651 | -0.651 | 1.699 | 1.917 | 1.421 | 0.948 | 0.529 | -0.651 | -0.651 | -0.651 | -0.651 | -0.651 |
| 213 | C_0080 | 5-Hydroxylysine | C16741 | HMDB00450 | 163.107 | 7.36 | -0.651 | -0.651 | -0.651 | -0.651 | -0.651 | 1.889 | 1.423 | 1.464 | 1.379 | 0.354 | -0.651 | -0.651 | -0.651 | -0.651 | -0.651 |
| 214 | A_0062 | *O*-Succinylhomoserine | C01118 | No ID | 218.069 | 12.98 | -0.569 | -0.569 | -0.569 | -0.569 | -0.569 | 1.624 | 1.599 | 2.078 | 0.960 | -0.569 | -0.569 | -0.569 | -0.569 | -0.569 | -0.569 |
| 215 | A_0032 | Quinolinic acid | C03722 | HMDB00232 | 166.014 | 17.06 | -0.635 | -0.635 | -0.635 | -0.635 | -0.635 | 1.789 | 0.722 | 1.385 | 2.038 | 0.416 | -0.635 | -0.635 | -0.635 | -0.635 | -0.635 |
| 216 | C_0134 | Glu-Glu | C01425 | No ID | 277.103 | 11.19 | -0.402 | -0.228 | -0.772 | -0.772 | -0.264 | 1.904 | 0.850 | 0.708 | 1.997 | 0.840 | -0.772 | -0.772 | -0.772 | -0.772 | -0.772 |
| 217 | C_0097 | $N^8$-Acetylspermidine | C01029 | HMDB02189 | 188.176 | 6.52 | -0.645 | -0.645 | -0.645 | -0.499 | -0.458 | 0.974 | 1.165 | 0.925 | 2.619 | 0.436 | -0.645 | -0.645 | -0.645 | -0.645 | -0.645 |
| 218 | C_0149 | Cysteine glutathione disulfide | C05526 | HMDB00656 | 427.095 | 11.94 | -0.532 | -0.374 | -0.362 | -0.541 | -0.273 | 1.026 | 0.692 | 0.726 | 2.323 | 1.473 | -0.831 | -0.831 | -0.831 | -0.831 | -0.831 |
| 219 | C_0099 | Gly-Leu | No ID | No ID | 189.123 | 10.12 | -0.583 | -0.527 | -0.588 | -0.538 | -0.587 | 1.714 | 1.259 | 0.807 | 1.513 | 1.396 | -0.773 | -0.773 | -0.773 | -0.773 | -0.773 |

| | | | | | | | | | | | | | | | | | | | | | |
|---|---|---|---|---|---|---|---|---|---|---|---|---|---|---|---|---|---|---|---|---|---|
| 220 | C_0089 | *N*-Acetylornithine | C00437 | HMDB03357 | 175.107 | 9.79 | -0.783 | -0.307 | -0.783 | -0.377 | -0.414 | 1.804 | 1.447 | 0.847 | 1.526 | 0.954 | -0.783 | -0.783 | -0.783 | -0.783 | -0.783 |
| 221 | C_0076 | Ala-Ala | C00993 | HMDB03459 | 161.091 | 9.52 | -0.820 | -0.276 | -0.820 | -0.120 | -0.397 | 1.711 | 1.567 | 1.085 | 1.346 | 0.823 | -0.820 | -0.820 | -0.820 | -0.820 | -0.820 |
| 222 | C_0052 | Thiaproline | No ID | No ID | 134.027 | 14.32 | -0.876 | -0.209 | -0.481 | -0.118 | -0.193 | 1.249 | 1.658 | 0.513 | 1.910 | 0.925 | -0.876 | -0.876 | -0.876 | -0.876 | -0.876 |
| 223 | C_0138 | Ophthalmic acid | No ID | HMDB05765 | 290.134 | 13.56 | -0.491 | -0.130 | -0.263 | 0.288 | -0.258 | 0.521 | 1.373 | 1.385 | 1.760 | 0.994 | -1.036 | -1.036 | -1.036 | -1.036 | -1.036 |
| 224 | A_0006 | 2-Hydroxybutyric acid | C05984 | HMDB00008 | 103.040 | 10.33 | -0.849 | 0.118 | -0.849 | -0.849 | 0.783 | 0.744 | 1.743 | 0.819 | 1.345 | 1.240 | -0.849 | -0.849 | -0.849 | -0.849 | -0.849 |
| 225 | A_0078 | Xanthosine | C01762 | HMDB00299 | 283.066 | 7.77 | -0.754 | 0.921 | -0.754 | -0.754 | 0.877 | -0.754 | 1.535 | 1.935 | 0.649 | 0.866 | -0.754 | -0.754 | -0.754 | -0.754 | -0.754 |
| 226 | A_0007 | Glyceric acid | C00258 | HMDB00139,H | 105.019 | 10.87 | 0.152 | 0.555 | -1.036 | 0.683 | 0.298 | 0.513 | 1.893 | 1.644 | 0.162 | 0.315 | -1.036 | -1.036 | -1.036 | -1.036 | -1.036 |
| 227 | C_0144 | NMN | C00455 | HMDB00229 | 335.064 | 21.20 | 0.051 | 0.785 | -0.071 | -0.011 | -0.044 | 0.420 | 1.812 | 1.456 | 0.628 | 0.792 | -1.164 | -1.164 | -1.164 | -1.164 | -1.164 |
| 228 | C_0009 | Sarcosine | C00213 | HMDB00271 | 90.055 | 9.73 | 0.266 | 1.148 | 0.194 | -0.233 | -0.114 | 0.643 | 1.218 | 1.736 | 0.547 | 0.521 | -1.185 | -1.185 | -1.185 | -1.185 | -1.185 |
| 229 | C_0136 | Guanosine | C00387 | HMDB00133 | 284.098 | 12.88 | 0.168 | 1.476 | 0.081 | 0.436 | 0.177 | 0.630 | 1.637 | 0.943 | 0.329 | 0.220 | -1.263 | -1.171 | -1.161 | -1.260 | -1.240 |
| 230 | C_0016 | GABA | C00334 | HMDB00112 | 104.071 | 7.92 | 0.140 | 0.568 | -0.066 | 0.729 | 0.224 | 0.380 | 1.664 | 0.809 | 1.233 | 0.367 | -1.599 | -1.027 | -0.828 | -1.599 | -0.996 |
| 231 | C_0101 | $N^6$,$N^6$,$N^6$-Trimethyllysine | C03793 | HMDB01325 | 189.159 | 7.39 | -0.376 | 0.255 | 1.035 | -0.008 | -0.007 | 0.745 | 1.990 | 0.953 | 0.674 | 0.487 | -1.150 | -1.150 | -1.150 | -1.150 | -1.150 |
| 232 | C_0087 | 3-Methylhistidine | C01152 | HMDB00479 | 170.092 | 7.71 | 0.039 | 0.178 | 0.198 | -0.146 | 0.602 | 1.353 | 1.426 | 0.997 | 0.679 | 0.819 | -1.229 | -1.229 | -1.229 | -1.229 | -1.229 |
| 233 | C_0017 | Choline | C00114 | HMDB00097 | 104.107 | 7.05 | -0.252 | 0.126 | -0.283 | -0.228 | 0.128 | 1.380 | 1.974 | 0.904 | 0.525 | 1.018 | -0.688 | -1.143 | -1.246 | -0.941 | -1.274 |
| 234 | A_0018 | *N*-Acetylalanine | No ID | HMDB00766 | 130.050 | 9.22 | -0.186 | 0.245 | -0.320 | -0.300 | -0.400 | 1.330 | 2.211 | 1.102 | 0.723 | 0.532 | -0.988 | -0.988 | -0.988 | -0.988 | -0.988 |
| 235 | C_0143 | XC0132 | - | - | 325.160 | 8.93 | -0.428 | 0.114 | -0.198 | -0.089 | 0.291 | 0.853 | 2.153 | 1.068 | 0.796 | 0.830 | -1.234 | -1.027 | -1.083 | -1.076 | -0.969 |
| 236 | A_0080 | *N*-Acetylglucosamine 1-phosphate | C04256 | HMDB01367 | 300.048 | 9.94 | -0.165 | 0.097 | -0.379 | -0.302 | -0.068 | 0.674 | 2.310 | 1.517 | 0.636 | 0.609 | -0.986 | -0.986 | -0.986 | -0.986 | -0.986 |
| 237 | C_0115 | 2'-Deoxycytidine | C00881 | HMDB00014 | 228.097 | 9.68 | -0.229 | 0.304 | -0.461 | 0.062 | -0.097 | 0.591 | 2.742 | 0.919 | 0.462 | 0.451 | -0.949 | -0.949 | -0.949 | -0.949 | -0.949 |
| 238 | C_0011 | β-Ala | C00099 | HMDB00056 | 90.055 | 7.55 | -0.224 | 0.098 | 0.081 | 0.006 | -0.212 | 0.608 | 2.503 | 1.176 | 0.805 | -0.230 | -1.392 | -0.123 | -1.142 | -1.033 | -0.921 |
| 239 | A_0079 | Sedoheptulose 7-phosphate | C05382 | HMDB01068 | 289.031 | 9.92 | -0.462 | -0.268 | -0.369 | 0.472 | -0.074 | 0.169 | 3.027 | 1.235 | -0.129 | -0.397 | -0.949 | -0.244 | -0.404 | -0.949 | -0.657 |
| 240 | C_0038 | 1-Methylhistamine | C05127 | HMDB00898 | 126.102 | 5.07 | -0.552 | 0.270 | 0.041 | -0.242 | 0.030 | -0.149 | 2.586 | 1.695 | -0.013 | 0.593 | -0.852 | -0.852 | -0.852 | -0.852 | -0.852 |
| 241 | C_0074 | Guanine | C00242 | HMDB00132 | 152.057 | 8.54 | -0.558 | -0.205 | -0.564 | 0.248 | -0.350 | 0.549 | 2.441 | 1.969 | 0.170 | 0.140 | -0.778 | -0.748 | -0.694 | -0.865 | -0.753 |
| 242 | A_0081 | *N*-Acetylglucosamine 6-phosphate | C00357 | HMDB01062 | 300.048 | 9.40 | -0.280 | -0.172 | -0.877 | -0.148 | -0.120 | 0.126 | 2.679 | 1.944 | 0.041 | -0.007 | -0.592 | -0.877 | -0.585 | -0.521 | -0.614 |
| 243 | C_0123 | Uridine | C00299 | HMDB00296 | 245.076 | 22.78 | -0.795 | 0.084 | -0.975 | -0.578 | -0.421 | 0.589 | 2.513 | 1.846 | -0.095 | 0.295 | -0.671 | -0.157 | -0.198 | -1.025 | -0.411 |
| 244 | C_0047 | Ile | C00407,C0641 | HMDB00172 | 132.102 | 10.51 | -1.179 | -0.455 | -1.087 | -0.740 | -0.698 | 0.955 | 2.217 | 1.478 | -0.290 | -0.053 | -0.585 | -0.127 | 0.942 | -0.810 | 0.433 |
| 245 | C_0072 | Met | C00073,C0085 | HMDB00696 | 150.058 | 11.01 | -1.435 | -0.326 | -1.277 | 0.526 | -0.422 | 0.462 | 2.495 | 1.275 | -0.096 | -0.074 | -0.918 | -0.157 | 0.359 | -0.751 | 0.340 |
| 246 | C_0055 | Hypoxanthine | C00262 | HMDB00157 | 137.046 | 11.43 | -1.249 | -0.635 | -0.940 | -0.047 | -0.251 | -0.171 | 2.764 | 1.020 | -0.816 | -0.484 | -0.091 | 0.992 | 0.443 | -0.536 | 0.002 |
| 247 | C_0023 | Uracil | C00106 | HMDB00300 | 113.034 | 22.76 | -1.197 | -0.447 | -1.095 | -0.348 | -0.940 | 0.487 | 2.306 | 1.476 | -0.113 | -0.231 | 0.134 | 1.158 | 0.061 | -0.298 | -0.955 |
| 248 | A_0039 | Decanoic acid | C01571 | HMDB00511 | 171.138 | 8.21 | -0.375 | -0.375 | -0.375 | -0.375 | -0.375 | -0.375 | 2.045 | 2.827 | -0.375 | -0.375 | -0.375 | -0.375 | -0.375 | -0.375 | -0.375 |
| 249 | A_0044 | Ascorbic acid | C00072 | HMDB00044 | 175.024 | 8.79 | -0.356 | -0.356 | -0.356 | -0.356 | -0.043 | -0.356 | 0.898 | 3.416 | -0.356 | -0.356 | -0.356 | -0.356 | -0.356 | -0.356 | -0.356 |
| 250 | C_0128 | XC0089 | - | - | 255.097 | 9.76 | 0.325 | 0.045 | -0.094 | 0.408 | -0.555 | -0.036 | 1.768 | 2.655 | -0.259 | -0.355 | -0.780 | -0.780 | -0.780 | -0.780 | -0.780 |
| 251 | A_0083 | Ribulose 1,5-diphosphate | C01182 | No ID | 308.977 | 17.01 | 0.137 | -0.813 | -0.813 | 1.301 | 0.460 | -0.813 | 2.011 | 1.435 | 0.623 | 0.540 | -0.813 | -0.813 | -0.813 | -0.813 | -0.813 |
| 252 | A_0064 | Myristoleic acid | C08322 | HMDB02000 | 225.185 | 7.69 | -0.723 | -0.723 | -0.723 | 0.335 | 1.324 | 0.230 | 1.924 | 2.048 | -0.011 | -0.065 | -0.723 | -0.723 | -0.723 | -0.723 | -0.723 |
| 253 | A_0010 | Hexanoic acid | C01585 | HMDB00535 | 115.076 | 9.28 | -0.568 | -0.568 | -0.568 | 0.876 | 1.759 | -0.568 | 1.605 | 2.005 | -0.568 | -0.568 | -0.568 | -0.568 | -0.568 | -0.568 | -0.568 |
| 254 | A_0093 | XA0055 | - | - | 368.995 | 15.42 | -0.471 | -0.471 | -0.471 | -0.471 | 1.617 | -0.471 | 2.547 | 1.493 | -0.471 | -0.471 | -0.471 | -0.471 | -0.471 | -0.471 | -0.471 |
| 255 | A_0067 | Ribose 5-phosphate | C00117 | HMDB01548 | 229.011 | 10.91 | -1.005 | 0.242 | -0.166 | 0.823 | 0.452 | 0.320 | 1.873 | 0.872 | -0.700 | -1.073 | -0.560 | -1.046 | -0.774 | 1.737 | -0.994 |
| 256 | A_0035 | Uric acid | C00366 | HMDB00289 | 167.021 | 9.13 | 0.450 | 0.783 | -1.869 | 0.468 | 0.047 | 0.258 | 0.798 | 0.029 | 0.715 | 0.225 | 0.678 | -1.869 | -1.869 | 0.793 | 0.364 |
| 257 | C_0058 | Tyramine | C00483 | HMDB00306 | 138.091 | 8.56 | 0.313 | -1.099 | -1.099 | 0.422 | 0.547 | 1.205 | 0.451 | 0.601 | 0.679 | 0.533 | -1.099 | -1.099 | -1.099 | -1.099 | 1.847 |
| 258 | A_0048 | XA0017 | - | - | 186.113 | 7.97 | 0.548 | 0.150 | -1.845 | 1.843 | -0.619 | -0.083 | -0.619 | 0.090 | 1.551 | 0.726 | -0.601 | -1.212 | 0.934 | -0.226 | -0.638 |
| 259 | A_0017 | Heptanoic acid | No ID | HMDB00666 | 129.091 | 8.91 | -0.373 | -0.373 | -0.373 | 1.973 | -0.373 | -0.373 | -0.373 | 2.880 | -0.373 | -0.373 | -0.373 | -0.373 | -0.373 | -0.373 | -0.373 |
| 260 | C_0093 | Serotonin | C00780 | HMDB00259 | 177.102 | 9.00 | -2.168 | 0.411 | 0.925 | 0.695 | -0.110 | -0.127 | 1.022 | 0.664 | -2.168 | 0.910 | 0.326 | 0.459 | -0.087 | -0.644 | -0.109 |
| 261 | A_0016 | 5-Oxohexanoic acid | C02129 | No ID | 129.055 | 9.45 | -0.378 | -0.378 | -0.378 | -0.378 | 2.249 | -0.378 | -0.378 | -0.378 | -0.378 | 2.662 | -0.378 | -0.378 | -0.378 | -0.378 | -0.378 |

C indicates the cation mode and A indicates the anion mode.

† Metabolites identified from HMT database based on m/z and migration time
