# Supplementary material for: Altered choline level in atherosclerotic lesions: Upregulation of choline transporter-like protein 1 in human coronary unstable plaque
Source: PLoS One. 2023 Feb 17;18(2):e0281730. doi: 10.1371/journal.pone.0281730 (PMC9937458; doi:10.1371/journal.pone.0281730)
Supplement: S7 Table — (PDF) [file pone.0281730.s007.PDF]

Supplementary table 7. Arterial and cardiac metabolite levels in rabbits fed a 0.5% cholesterol diet

ID	Metabolite	KEGG ID	HMDB ID	Concentration (nmol/g)																				
				non-injured artery					injured artery					heart					non-injured artery		injured artery		heart	
				1	2	3	4	5	6	7	8	9	10	11	12	13	14	15	Mean	S.D.	Mean	S.D.	Mean	S.D.
A_0001	Glyoxylic acid	C00048	HMDB00119	N.D.	N.D.	N.D.	N.D.	N.D.	11	6.8	N.D.	13	N.D.	N.D.	N.D.	N.D.	N.D.	N.D.	N.D.	N.A.	10	3.3	N.D.	N.A.
A_0002	Glycolic acid	C00160	HMDB00115	N.D.	N.D.	N.D.	N.D.	N.D.	N.D.	N.D.	N.D.	N.D.	N.D.	N.D.	N.D.	N.D.	N.D.	N.D.	N.D.	N.A.	N.D.	N.A.	N.D.	N.A.
A_0003	Pyruvic acid	C00022	HMDB00243	25	N.D.	24	N.D.	N.D.	N.D.	30	N.D.	18	N.D.	N.D.	N.D.	N.D.	N.D.	95	24	0.6	24	8.5	95	N.A.
A_0004	Lactic acid	C00186,C0025	HMDB00190,H	1,737	1,111	1,469	1,134	1,398	2,891	3,480	3,146	2,014	1,793	8,100	12,680	14,118	12,442	14,737	1,370	259	2,665	730	12,415	2,597
A_0005	3-Hydroxybutyric acid	C01089,C0319	HMDB00011,H	18	13	19	14	19	32	28	30	32	24	130	158	199	142	167	16	2.9	29	3.1	159	26
A_0006	2-Hydroxybutyric acid	C05984	HMDB00008	N.D.	0.8	N.D.	N.D.	1.4	1.3	2.2	1.4	1.8	1.8	N.D.	N.D.	N.D.	N.D.	N.D.	1.1	0.4	1.7	0.3	N.D.	N.A.
A_0008	Fumaric acid	C00122	HMDB00134	7.0	12	7.7	11	6.7	10	16	16	7.4	7.0	300	334	479	278	411	9.0	2.6	11	4.3	361	83
A_0009	2-Oxoisovaleric acid	C00141	HMDB00019	N.D.	N.D.	N.D.	N.D.	N.D.	N.D.	N.D.	N.D.	N.D.	N.D.	N.D.	N.D.	N.D.	N.D.	N.D.	N.D.	N.A.	N.D.	N.A.	N.D.	N.A.
A_0012	Succinic acid	C00042	HMDB00254	22	26	23	20	18	20	28	24	19	22	174	455	621	376	284	22	3.2	23	3.7	382	170
A_0020	Malic acid	C00149,C0049	HMDB00156,H	52	78	62	59	40	57	80	77	51	49	953	1,022	1,494	882	1,171	58	14	63	15	1,105	243
A_0025	2-Oxoglutaric acid	C00026	HMDB00208	N.D.	N.D.	N.D.	N.D.	N.D.	N.D.	N.D.	N.D.	N.D.	N.D.	N.D.	N.D.	N.D.	N.D.	N.D.	N.D.	N.A.	N.D.	N.A.	N.D.	N.A.
A_0034	Phosphoenolpyruvic acid	C00074	HMDB00263	7.2	16	7.4	29	21	3.4	5.5	6.0	4.3	6.1	N.D.	N.D.	N.D.	N.D.	N.D.	16	9.2	5.1	1.2	N.D.	N.A.
A_0036	Dihydroxyacetone phosphate	C00111	HMDB01473	24	39	17	23	9.8	20	15	17	11	6.8	381	172	379	477	272	23	11	14	5.2	336	117
A_0037	Glyceraldehyde 3-phosphate	C00118,C0066	HMDB01112	N.D.	N.D.	N.D.	N.D.	N.D.	N.D.	N.D.	N.D.	N.D.	N.D.	N.D.	N.D.	N.D.	50	N.D.	N.D.	N.A.	N.D.	N.A.	50	N.A.
A_0038	Glycerol 3-phosphate	C00093	HMDB00126	120	177	166	120	235	242	331	240	178	233	1,479	1,658	1,954	1,521	1,462	164	48	245	55	1,615	205
A_0041	*cis*-Aconitic acid	C00417	HMDB00072	37	18	44	2.8	3.0	16	8.2	5.4	18	15	22	13	35	19	16	21	19	12	5.3	21	8.5
A_0046	2-Phosphoglyceric acid	C00631	HMDB03391	12	18	15	20	15	6.1	9.8	11	6.6	7.5	N.D.	N.D.	N.D.	N.D.	N.D.	16	2.9	8.1	2.0	N.D.	N.A.
A_0047	3-Phosphoglyceric acid	C00197	HMDB00807	76	112	84	131	92	42	64	67	46	46	24	N.D.	55	20	12	99	22	53	12	28	19
A_0052	Isocitric acid	C00311	HMDB00193	20	15	25	2.3	3.2	14	7.4	5.9	21	18	26	13	39	18	16	13	10	13	6.4	23	10
A_0054	Citric acid	C00158	HMDB00094	1,768	895	2,193	152	169	706	297	265	1,014	751	938	462	1,398	922	657	1,036	926	607	320	875	353
A_0056	Gluconic acid	C00257	HMDB00625	7.7	12	34	6.7	3.3	11	12	9.7	8.0	7.5	N.D.	10	N.D.	18	N.D.	13	12	9.7	1.9	14	5.6
A_0057	Erythrose 4-phosphate	C00279,C0360	HMDB01321	N.D.	N.D.	N.D.	N.D.	N.D.	N.D.	N.D.	N.D.	N.D.	N.D.	N.D.	N.D.	N.D.	N.D.	N.D.	N.D.	N.A.	N.D.	N.A.	N.D.	N.A.
A_0066	Ribulose 5-phosphate	C00199,C0110	HMDB00618	34	73	46	74	67	69	111	79	44	41	103	136	98	84	100	59	18	69	29	104	19
A_0067	Ribose 5-phosphate	C00117	HMDB01548	12	24	20	30	26	25	40	31	15	12	17	12	14	39	12	23	6.8	25	12	19	11
A_0071	Glucose 1-phosphate	C00103	HMDB01586	92	58	88	9.5	6.4	28	33	15	60	25	356	258	600	405	175	51	41	32	17	359	161
A_0072	Glucose 6-phosphate	C00668,C0117	HMDB01401	73	165	130	41	15	26	25	16	21	11	1,011	2,083	1,391	1,080	1,232	85	62	20	6.3	1,359	430
A_0074	Fructose 6-phosphate	C05345,C0008	HMDB00124	N.D.	N.D.	N.D.	9.3	6.5	N.D.	9.5	8.1	N.D.	N.D.	186	552	311	261	320	7.9	2.0	8.8	1.0	326	137
A_0077	6-Phosphogluconic acid	C00345	HMDB01316	17	39	11	16	9.9	23	20	12	14	8.4	9.3	9.4	10	7.5	9.7	19	12	15	6.0	9.2	1.0
A_0079	Sedoheptulose 7-phosphate	C05382	HMDB01068	2.4	3.3	2.8	6.9	4.2	5.4	19	11	4.0	2.7	N.D.	3.4	2.6	N.D.	1.4	3.9	1.8	8.4	6.8	2.5	1.0
A_0084	dTMP	C00364	HMDB01227	N.D.	N.D.	N.D.	N.D.	N.D.	N.D.	N.D.	N.D.	N.D.	N.D.	N.D.	N.D.	N.D.	N.D.	N.D.	N.D.	N.A.	N.D.	N.A.	N.D.	N.A.
A_0085	CMP	C00055	HMDB00095	6.6	6.4	12	5.3	15	8.3	2.0	1.6	8.8	7.1	31	30	28	32	26	9.2	4.4	5.6	3.5	29	2.6
A_0086	UMP	C00105	HMDB00288	7.4	4.5	9.7	7.3	19	15	3.0	2.0	21	9.8	56	79	66	62	58	9.6	5.7	10	8.0	64	9.2
A_0087	cAMP	C00575	HMDB00058	N.D.	N.D.	N.D.	N.D.	N.D.	N.D.	N.D.	N.D.	N.D.	N.D.	3.7	3.4	4.2	4.9	4.6	N.D.	N.A.	N.D.	N.A.	4.2	0.6
A_0088	Fructose 1,6-diphosphate	C00354	HMDB01058	47	61	76	30	20	24	24	22	34	23	1,028	1,815	1,305	1,324	1,287	47	23	25	4.6	1,352	286
A_0089	cGMP	C00942	HMDB01314	N.D.	N.D.	N.D.	N.D.	N.D.	N.D.	N.D.	N.D.	N.D.	N.D.	2.0	2.0	2.6	1.9	N.D.	N.D.	N.A.	N.D.	N.A.	2.1	0.3
A_0090	AMP	C00020	HMDB00045	80	53	87	76	103	90	50	58	146	57	2,230	3,517	3,554	2,640	2,516	80	18	80	40	2,892	607
A_0091	IMP	C00130	HMDB00175	86	23	127	20	39	14	N.D.	N.D.	13	8.5	3,144	1,303	1,245	1,256	1,563	59	46	12	2.9	1,702	816
A_0092	GMP	C00144	HMDB01397	20	16	32	23	46	33	9.0	9.9	35	24	142	164	138	109	134	28	12	22	12	138	20
A_0094	CoA_divalent	C00010	HMDB01423	1.3	1.1	1.4	0.5	0.4	0.7	0.14	0.4	0.6	0.7	17	40	22	20	22	1.0	0.4	0.5	0.2	24	9.1
A_0095	PRPP	C00119	HMDB00280	N.D.	N.D.	N.D.	N.D.	N.D.	N.D.	N.D.	N.D.	N.D.	N.D.	N.D.	N.D.	N.D.	N.D.	N.D.	N.D.	N.A.	N.D.	N.A.	N.D.	N.A.
A_0097	dTDP	C00363	HMDB01274	N.D.	N.D.	N.D.	N.D.	N.D.	N.D.	N.D.	N.D.	N.D.	N.D.	N.D.	N.D.	N.D.	N.D.	N.D.	N.D.	N.A.	N.D.	N.A.	N.D.	N.A.
A_0098	CDP	C00112	HMDB01546	5.0	0.8	5.6	0.3	0.4	0.6	0.12	N.D.	0.6	0.6	3.2	2.0	2.3	6.5	4.6	2.4	2.6	0.5	0.2	3.7	1.8
A_0099	UDP	C00015	HMDB00295	18	2.7	18	0.4	1.5	2.3	0.6	N.D.	2.8	2.3	8.3	8.0	7.0	17	18	8.2	9.1	2.0	1.0	12	5.3
A_0100	Acetyl CoA_divalent	C00024	HMDB01206	1.9	1.7	1.6	0.7	0.8	1.6	0.7	0.8	1.7	1.6	11	1.1	14	5.7	2.5	1.3	0.6	1.3	0.5	6.9	5.5
A_0103	Malonyl CoA_divalent	C00083	HMDB01175	N.D.	N.D.	N.D.	N.D.	N.D.	N.D.	N.D.	N.D.	N.D.	N.D.	N.D.	N.D.	N.D.	N.D.	N.D.	N.D.	N.A.	N.D.	N.A.	N.D.	N.A.
A_0105	ADP	C00008	HMDB01341	193	146	199	87	82	55	37	44	55	53	628	710	896	1,298	1,271	141	56	49	8.0	961	312
A_0106	GDP	C00035	HMDB01201	29	10	32	4.3	4.2	7.3	2.5	3.3	6.6	6.2	62	48	68	68	62	16	14	5.2	2.1	62	8.1
A_0111	dCTP	C00458	HMDB00998	N.D.	N.D.	N.D.	N.D.	N.D.	N.D.	N.D.	N.D.	N.D.	N.D.	N.D.	N.D.	N.D.	N.D.	N.D.	N.D.	N.A.	N.D.	N.A.	N.D.	N.A.
A_0112	dTTP	C00459	HMDB01342	N.D.	N.D.	N.D.	N.D.	N.D.	N.D.	N.D.	N.D.	N.D.	N.D.	N.D.	N.D.	N.D.	N.D.	N.D.	N.D.	N.A.	N.D.	N.A.	N.D.	N.A.
A_0113	CTP	C00063	HMDB00082	N.D.	N.D.	N.D.	N.D.	N.D.	N.D.	N.D.	N.D.	N.D.	N.D.	N.D.	N.D.	N.D.	N.D.	N.D.	N.D.	N.A.	N.D.	N.A.	N.D.	N.A.
A_0114	UTP	C00075	HMDB00285	0.6	0.4	0.6	N.D.	0.4	0.3	0.3	0.13	N.D.	0.2	1.3	N.D.	N.D.	4.3	4.1	0.5	0.08	0.2	0.08	3.2	1.7
A_0116	dATP	C00131	HMDB01532	N.D.	N.D.	N.D.	N.D.	N.D.	N.D.	N.D.	N.D.	N.D.	N.D.	N.D.	N.D.	N.D.	N.D.	N.D.	N.D.	N.A.	N.D.	N.A.	N.D.	N.A.
A_0117	ATP	C00002	HMDB00538	15	40	13	40	47	9.6	26	12	7.3	8.3	120	55	173	446	543	31	16	13	7.7	267	214
A_0118	GTP	C00044	HMDB01273	2.4	2.6	2.2	2.0	2.1	0.9	0.9	1.0	0.9	0.8	19	8.1	23	25	34	2.3	0.2	0.9	0.09	22	9.7
A_0126	NAD^{+}	C00003	HMDB00902	64	66	59	20	42	54	33	28	34	40	3.4	1.1	12	3.8	3.6	50	19	38	10	4.8	4.3
A_0128	$NADP^{+}$	C00006	HMDB00217	7.9	7.8	8.9	2.1	2.2	5.3	2.6	2.9	2.7	4.1	N.D.	N.D.	N.D.	N.D.	N.D.	5.8	3.4	3.5	1.2	N.D.	N.A.
C_0005	Gly	C00037	HMDB00123	810	897	744	421	934	869	948	744	753	1,034	844	679	583	832	1,909	761	204	870	125	969	537
C_0008	Putrescine	C00134	HMDB01414	2.7	8.5	4.8	4.5	4.9	13	20	14	13	7.0	31	36	18	26	42	5.1	2.1	13	4.7	30	9.2
C_0009	Sarcosine	C00213	HMDB00271	2.5	4.0	2.4	1.6	1.8	3.1	4.1	5.0	3.0	2.9	N.D.	N.D.	N.D.	N.D.	N.D.	2.5	0.9	3.6	0.9	N.D.	N.A.
C_0010	Ala	C00041,C0013	HMDB00161,H	175	197	228	146	148	216	259	241	179	161	1,968	2,396	3,270	1,840	2,839	179	35	211	41	2,463	598
C_0011	β-Ala	C00099	HMDB00056	27	32	32	31	28	40	67	48	42	27	10	29	14	16	17	30	2.3	45	15	17	7.0
C_0014	*N,N*-Dimethylglycine	C01026	HMDB00092	1.8	1.3	N.D.	N.D.	1.4	2.9	1.6	1.2	1.1	1.7	7.1	4.3	4.3	3.6	4.2	1.5	0.2	1.7	0.7	4.7	1.4
C_0016	GABA	C00334	HMDB00112	4.5	5.7	4.0	6.1	4.8	5.2	8.5	6.3	7.4	5.1	N.D.	1.5	2.0	N.D.	1.6	5.0	0.8	6.5	1.5	1.7	0.3
C_0017	Choline	C00114	HMDB00097	188	227	184	190	227	357	418	307	268	319	142	95	84	116	82	203	22	334	57	104	25
C_0018	Ser	C00065,C0071	HMDB00187,H	111	131	102	121	129	182	237	181	221	176	221	217	229	267	347	119	12	199	28	256	54
C_0021	Cytosine	C00380	HMDB00630	N.D.	N.D.	N.D.	N.D.	N.D.	N.D.	N.D.	N.D.	N.D.	N.D.	N.D.	N.D.	N.D.	N.D.	N.D.	N.D.	N.A.	N.D.	N.A.	N.D.	N.A.
C_0023	Uracil	C00106	HMDB00300	7.3	12	8.0	13	9.1	19	31	26	15	14	16	23	16	13	9.0	10	2.6	21	7.4	16	5.2
C_0024	Creatinine	C00791	HMDB00562	28	29	34	16	32	40	35	31	29	30	140	97	132	119	124	28	6.9	33	4.5	122	16
C_0025	Pro	C00148,C0076	HMDB00162,H	35	52	43	37	57	109	122	100	100	96	102	101	122	105	135	45	9.4	106	11	113	15
C_0027	Val	C00183,C0641	HMDB00883	48	61	66	49	79	127	137	126	106	108	110	123	159	92	146	60	13	121	13	126	27
C_0028	Betaine	C00719	HMDB00043	112	209	167	132	109	197	325	198	209	126	1,039	1,065	1,762	1,641	844	146	42	211	72	1,270	405
C_0029	Thr	C00188,C0082	HMDB00167	90	86	92	78	106	162	156	154	149	142	210	179	236	177	261	90	10	153	7.3	213	37
C_0030	Homoserine	C00263	HMDB00719	N.D.	N.D.	N.D.	N.D.	N.D.	0.7	0.9	1.6	1.0	1.3	0.7	1.2	N.D.	N.D.	1.1	N.D.	N.A.	1.1	0.3	1.0	0.2
C_0031	Betaine aldehyde_+H_2O	C00576	HMDB01252	N.D.	N.D.	N.D.	N.D.	N.D.	0.8	0.9	0.7	0.6	0.4	N.D.	N.D.	N.D.	N.D.	N.D.	N.D.	N.A.	0.7	0.2	N.D.	N.A.
C_0033	Cys	C00097,C0073	HMDB00574,H	5.9	5.7	9.5	1.1	0.9	2.1	1.7	0.8	9.3	5.5	N.D.	N.D.	N.D.	N.D.	N.D.	4.6	3.6	3.9	3.5	N.D.	N.A.
C_0039	Thymine	C00178	HMDB00262	N.D.	N.D.	N.D.	N.D.	N.D.	N.D.	N.D.	N.D.	N.D.	N.D.	N.D.	N.D.	N.D.	N.D.	N.D.	N.D.	N.A.	N.D.	N.A.	N.D.	N.A.
C_0044	Hydroxyproline	C01157	HMDB00725	7.1	6.2	7.5	4.8	7.1	13	12	9.6	7.6	8.4	20	14	18	13	20	6.5	1.1	10	2.2	17	3.3
C_0046	Creatine	C00300	HMDB00064	1,026	1,237	1,303	762	1,133	764	1,040	782	627	619	458	447	459	486	549	1,092	212	766	170	480	41
C_0047	Ile	C00407,C0641	HMDB00172	26	36	27	32	33	57	76	65	39	42	35	41	57	31	50	31	4.4	56	16	43	11
C_0048	Leu	C00123,C0157	HMDB00687	34	44	47	42	50	96	125	103	75	72	74	91	121	66	106	44	6.0	94	21	91	23
C_0050	Asn	C00152,C0190	HMDB00168	28	43	41	28	33	56	72	65	75	47	163	199	270	197	189	35	6.9	63	11	204	40
C_0051	Ornithine	C00077,C0051	HMDB00214,H	6.7	9.0	7.8	5.8	9.6	15	13	18	12	18	34	32	60	43	78	7.8	1.5	15	2.6	49	19
C_0053	Asp	C00049,C0040	HMDB00191,H	158	149	196	308	177	244	276	243	324	223	770	735	801	322	730	198	64	262	40	672	197
C_0054	Adenine	C00147	HMDB00034	1.7	2.4	1.3	2.7	0.8	2.0	3.1	2.4	2.0	1.1	3.7	8.9	10	4.6	7.2	1.8	0.8	2.1	0.7	7.0	2.8
C_0055	Hypoxanthine	C00262	HMDB00157	17	41	29	63	55	58	170	104	34	47	62	103	82	45	65	41	19	83	56	71	22
C_0056	Anthranilic acid	C00108	HMDB01123	N.D.	N.D.	N.D.	N.D.	N.D.	N.D.	N.D.	N.D.	N.D.	N.D.	N.D.	N.D.	N.D.	N.D.	N.D.	N.D.	N.A.	N.D.	N.A.	N.D.	N.A.
C_0058	Tyramine	C00483	HMDB00306	0.3	N.D.	N.D.	0.3	0.4	0.5	0.3	0.4	0.4	0.3	N.D.	N.D.	N.D.	N.D.	0.6	0.3	0.02	0.4	0.06	0.6	N.A.
C_0066	Spermidine	C00315	HMDB01257	45	56	74	24	35	44	24	21	70	47	93	95	165	102	92	47	19	41	20	109	31
C_0067	Gln	C00064,C0030	HMDB00641,H	535	779	705	452	551	539	654	559	470	426	10,803	9,262	10,896	11,740	13,121	604	134	529	88	11,164	1,413
C_0068	Lys	C00047,C0073	HMDB00182,H	48	65	63	48	70	133	154	159	186	157	119	129	192	86	170	59	10	158	19	140	42
C_0070	Glu	C00025,C0021	HMDB00148,H	1,896	2,339	1,937	1,673	2,006	2,481	2,992	2,414	2,017	1,919	4,991	7,048	5,106	3,727	3,409	1,970	241	2,365	427	4,856	1,437
C_0072	Met	C00073,C0085	HMDB00696	16	25	17	32	24	32	49	39	27	27	20	27	31	22	31	23	6.8	35	9.3	26	5.1
C_0074	Guanine	C00242	HMDB00132	2.9	5.0	2.8	7.8	4.1	9.6	21	18	7.3	7.1	1.5	1.7	2.1	1.0	1.7	4.5	2.0	13	6.5	1.6	0.4
C_0075	His	C00135,C0076	HMDB00177	27	37	49	27	29	59	76	74	78	50	372	391	842	456	327	34	9.4	67	12	478	209
C_0082	Phe	C00079,C0205	HMDB00159	16	22	24	22	24	53	63	53	44	40	64	69	84	73	79	22	3.3	50	9.0	74	7.6
C_0091	Arg	C00062,C0079	HMDB00517,H	36	62	70	57	59	108	133	157	139	114	239	250	376	312	318	57	13	130	20	299	56
C_0092	Citrulline	C00327	HMDB00904	10	17	11	10	17	20	28	16	20	26	24	39	55	32	52	13	3.8	22	5.0	41	13
C_0095	Tyr	C00082,C0153	HMDB00158	16	25	21	31	26	47	63	49	51	43	43	55	52	47	58	24	5.7	51	7.6	51	6.0
C_0106	Spermine	C00750	HMDB01256	24	19	46	4.4	3.7	6.1	3.3	2.1	12	13	34	29	38	37	28	19	17	7.3	5.0	33	4.6
C_0108	Trp	C00078,C0052	HMDB00929	6.3	8.1	7.4	6.0	9.0	15	16	14	13	12	19	20	17	17	23	7.3	1.2	14	1.5	19	2.6
C_0114	Carnosine	C00386	HMDB00033	4.1	13	37	9.9	7.8	4.2	7.7	10	3.9	2.0	509	951	566	739	767	14	13	5.6	3.3	706	176

C_0121	Thymidine	C00214	HMDB00273	N.D.	N.D.	N.D.	N.D.	N.D.	N.D.	N.D.	N.D.	N.D.	N.D.	N.D.	N.D.	N.D.	N.D.	N.D.	N.D.	N.A.	N.D.	N.A.	N.D.	N.A.
C_0122	Cytidine	C00475	HMDB00089	13	21	13	15	12	21	42	32	17	15	34	44	41	27	29	15	3.6	25	11	35	7.3
C_0123	Uridine	C00299	HMDB00296	59	94	51	67	74	115	193	166	87	103	64	85	83	49	74	69	16	133	45	71	15
C_0132	Adenosine	C00212	HMDB00050	23	15	14	138	16	166	56	69	165	73	7.5	24	9.4	14	12	41	54	106	55	13	6.6
C_0133	Inosine	C00294	HMDB00195	681	966	824	566	694	541	941	723	459	530	240	679	605	239	365	746	153	639	195	426	206
C_0136	Guanosine	C00387	HMDB00133	62	116	58	73	62	81	123	94	69	64	2.7	6.5	6.9	2.8	3.6	75	24	86	24	4.5	2.1
C_0141	Glutathione (GSSG)_divalent	C00127	HMDB03337	96	144	79	129	250	204	273	172	136	145	460	474	352	411	477	140	67	186	55	435	53
C_0142	Glutathione (GSH)	C00051	HMDB00125	516	469	397	281	190	64	105	84	76	82	1,318	1,780	1,201	1,377	1,326	371	135	82	15	1,400	222
C_0148	*S*-Adenosylmethionine	C00019	HMDB01185	4.7	8.2	8.0	4.6	4.1	7.0	7.8	6.6	6.5	4.8	16	26	27	19	18	5.9	2.0	6.6	1.1	21	5.0

C and A indicate cation and anion mode, respectively.

N.D.: Not Detected

N.A.: Not Available
