# Supplementary material for: Altered choline level in atherosclerotic lesions: Upregulation of choline transporter-like protein 1 in human coronary unstable plaque
Source: PLoS One. 2023 Feb 17;18(2):e0281730. doi: 10.1371/journal.pone.0281730 (PMC9937458; doi:10.1371/journal.pone.0281730)
Supplement: S8 Table — (PDF) [file pone.0281730.s008.PDF]

Supplementary table 8. Relative amount of arterial and cardiac metabolites in rabbits fed a 0.5% cholesterol diet

ID	HMT DB †				m/z	MT/RT	Relative Area																					Comparative Analysis					
							non-injured artery					injured artery					heart					non-injured artery		injured artery		heart		injured artery vs non-injured artery		heart vs non-injured artery		heart vs injured artery	
	Compound name	Pathway Label	KEGG ID	HMDB ID			1	2	3	4	5	6	7	8	9	10	11	12	13	14	15	Mean	S.D.	Mean	S.D.	Mean	S.D.	Injured artery to non-injured artery ratio	p-value ‖	Heart to non-injured artery ratio	p-value ‖	Heart to injured artery ratio	p-value ‖
C_0030	Homoserine	Homoserine	C00263	HMDB00719	120.066	10.37	N.D.	N.D.	N.D.	N.D.	N.D.	1.2E-04	1.5E-04	2.5E-04	1.5E-04	2.0E-04	1.1E-04	1.9E-04	N.D.	N.D.	1.7E-04	N.D.	N.A.	1.7E-04	5.3E-05	1.6E-04	3.9E-05	1<	N.A.	1<	N.A.	0.9	0.640
C_0112	β-Ala-Lys	b-Ala-Lys	C05341	No ID	218.149	7.00	N.D.	N.D.	N.D.	N.D.	N.D.	6.8E-04	4.2E-04	3.6E-04	5.0E-04	3.9E-04	N.D.	2.1E-04	1.9E-04	2.6E-04	2.3E-04	N.D.	N.A.	4.7E-04	1.3E-04	2.2E-04	3.0E-05	1<	N.A.	1<	N.A.	0.5	0.011 *
A_0001	Glyoxylic acid	Glyoxylic acid	C00048	HMDB00119	72.993	12.44	N.D.	N.D.	N.D.	N.D.	N.D.	1.2E-04	7.1E-05	N.D.	1.4E-04	N.D.	N.D.	N.D.	N.D.	N.D.	N.D.	N.D.	N.A.	1.1E-04	3.4E-05	N.D.	N.A.	1<	N.A.	N.A.	N.A.	<1	N.A.
A_0032	Quinolinic acid	Quinolinic acid	C03722	HMDB00232	166.014	17.06	N.D.	N.D.	N.D.	N.D.	N.D.	1.1E-04	6.1E-05	9.1E-05	1.2E-04	4.7E-05	N.D.	N.D.	N.D.	N.D.	N.D.	N.D.	N.A.	8.6E-05	3.1E-05	N.D.	N.A.	1<	N.A.	N.A.	N.A.	<1	N.A.
A_0039	Decanoic acid	Decanoic acid	C01571	HMDB00511	171.138	8.21	N.D.	N.D.	N.D.	N.D.	N.D.	N.D.	1.8E-04	2.3E-04	N.D.	N.D.	N.D.	N.D.	N.D.	N.D.	N.D.	N.D.	N.A.	2.1E-04	4.0E-05	N.D.	N.A.	1<	N.A.	N.A.	N.A.	<1	N.A.
A_0062	O-Succinylhomoserine	O-Succinylhomoserine	C01118	No ID	218.069	12.98	N.D.	N.D.	N.D.	N.D.	N.D.	1.6E-05	1.6E-05	2.0E-05	1.1E-05	N.D.	N.D.	N.D.	N.D.	N.D.	N.D.	N.D.	N.A.	1.6E-05	3.4E-06	N.D.	N.A.	1<	N.A.	N.A.	N.A.	<1	N.A.
C_0031	Betaine aldehyde_+H_2O	BTL	C00576	HMDB01252	120.102	7.67	N.D.	N.D.	N.D.	N.D.	N.D.	2.2E-04	2.4E-04	1.9E-04	1.5E-04	1.1E-04	N.D.	N.D.	N.D.	N.D.	N.D.	N.D.	N.A.	1.8E-04	5.2E-05	N.D.	N.A.	1<	N.A.	N.A.	N.A.	<1	N.A.
C_0080	5-Hydroxylysine	5-Hydroxylysine	C16741	HMDB00450	163.107	7.36	N.D.	N.D.	N.D.	N.D.	N.D.	1.4E-04	1.1E-04	1.1E-04	1.1E-04	5.4E-05	N.D.	N.D.	N.D.	N.D.	N.D.	N.D.	N.A.	1.1E-04	3.1E-05	N.D.	N.A.	1<	N.A.	N.A.	N.A.	<1	N.A.
C_0084	Tyr-Arg_divalent	Tyr-Arg_divalent	No ID	No ID	169.594	7.94	1.8E-05	1.6E-05	1.9E-05	2.0E-05	9.9E-06	2.7E-04	4.0E-04	2.0E-04	1.8E-04	2.0E-04	N.D.	N.D.	N.D.	N.D.	N.D.	1.7E-05	4.0E-06	2.5E-04	9.0E-05	N.D.	N.A.	15	0.004 **	<1	N.A.	<1	N.A.
C_0097	N^8-Acetylspermidine	N8-Acetylspermidine	C01029	HMDB02189	188.176	6.52	N.D.	N.D.	N.D.	5.2E-05	6.6E-05	5.7E-04	6.4E-04	5.6E-04	1.2E-03	3.8E-04	N.D.	N.D.	N.D.	N.D.	N.D.	5.9E-05	1.0E-05	6.6E-04	2.9E-04	N.D.	N.A.	11	0.010 **	<1	N.A.	<1	N.A.
C_0130	Glycerophosphocholine	GPCho	C00670	HMDB00086	258.109	22.32	2.3E-03	2.0E-03	3.3E-03	2.0E-03	2.0E-03	2.6E-02	2.3E-02	2.6E-02	3.4E-02	1.9E-02	1.5E-01	1.4E-01	1.8E-01	1.5E-01	1.1E-01	2.3E-03	5.3E-04	2.6E-02	5.5E-03	1.5E-01	2.7E-02	11	6.4E-04 ***	64	2.7E-04 ***	5.7	3.9E-04 ***
C_0099	Gly-Leu	Gly-Leu	No ID	No ID	189.123	10.12	6.7E-04	8.7E-04	6.5E-04	8.3E-04	6.6E-04	8.8E-03	7.2E-03	5.6E-03	8.1E-03	7.7E-03	N.D.	N.D.	N.D.	N.D.	N.D.	7.4E-04	1.0E-04	7.5E-03	1.2E-03	N.D.	N.A.	10	2.2E-04 ***	<1	N.A.	<1	N.A.
A_0044	Ascorbic acid	Ascorbic acid	C00072	HMDB00044	175.024	8.79	N.D.	N.D.	N.D.	N.D.	1.7E-04	N.D.	6.9E-04	2.1E-03	N.D.	N.D.	N.D.	N.D.	N.D.	N.D.	N.D.	1.7E-04	N.A.	1.4E-03	9.8E-04	N.D.	N.A.	8.0	N.A.	<1	N.A.	<1	N.A.
C_0089	N-Acetylornithine	N-AcOrn	C00437	HMDB03357	175.107	9.79	N.D.	2.0E-04	N.D.	1.7E-04	1.6E-04	1.1E-03	9.4E-04	6.9E-04	9.7E-04	7.3E-04	N.D.	N.D.	N.D.	N.D.	N.D.	1.8E-04	2.3E-05	8.8E-04	1.7E-04	N.D.	N.A.	5.0	5.9E-04 ***	<1	N.A.	<1	N.A.
C_0149	Cysteine glutathione disulfide	Cysteine glutathione disulfi	C05526	HMDB00656	427.095	11.94	2.8E-04	4.2E-04	4.3E-04	2.7E-04	5.1E-04	1.7E-03	1.4E-03	1.4E-03	2.9E-03	2.1E-03	N.D.	N.D.	N.D.	N.D.	N.D.	3.8E-04	1.1E-04	1.9E-03	6.2E-04	N.D.	N.A.	5.0	0.005 **	<1	N.A.	<1	N.A.
C_0119	Cystine	Cystine	C00491,C0142	HMDB00192	241.031	11.28	4.6E-05	5.4E-05	9.1E-05	N.D.	N.D.	2.2E-04	9.1E-05	1.3E-04	6.7E-04	3.5E-04	N.D.	N.D.	N.D.	N.D.	N.D.	6.4E-05	2.4E-05	2.9E-04	2.4E-04	N.D.	N.A.	4.6	0.097	<1	N.A.	<1	N.A.
C_0134	Glu-Glu	Glu-Glu	C01425	No ID	277.103	11.19	1.6E-05	2.4E-05	N.D.	N.D.	2.2E-05	1.2E-04	7.2E-05	6.5E-05	1.2E-04	7.1E-05	N.D.	N.D.	N.D.	N.D.	N.D.	2.1E-05	4.1E-06	9.0E-05	2.8E-05	N.D.	N.A.	4.3	0.005 **	<1	N.A.	<1	N.A.
A_0082	N-Acetylneuraminic acid	NeuNAc	C00270	HMDB00230	308.098	7.25	6.7E-05	7.4E-05	N.D.	7.0E-05	8.0E-05	2.6E-04	3.0E-04	2.3E-04	4.9E-04	2.6E-04	5.7E-04	4.8E-04	7.9E-04	6.5E-04	7.4E-04	7.3E-05	6.0E-06	3.1E-04	1.0E-04	6.4E-04	1.3E-04	4.2	0.007 **	8.9	5.1E-04 ***	2.1	0.002 **
A_0053	XA0019	XA0019	No ID	No ID	191.019	8.47	7.3E-04	9.1E-04	7.2E-04	1.0E-03	1.2E-03	4.4E-03	6.0E-03	3.6E-03	2.9E-03	1.7E-03	5.6E-04	1.1E-03	4.2E-04	6.4E-04	5.8E-04	9.2E-04	2.1E-04	3.7E-03	1.6E-03	6.6E-04	2.6E-04	4.1	0.016 *	0.7	0.129	0.2	0.012 *
C_0076	Ala-Ala	Ala-Ala	C00993	HMDB03459	161.091	9.52	N.D.	2.7E-04	N.D.	3.4E-04	2.1E-04	1.2E-03	1.2E-03	9.4E-04	1.1E-03	8.1E-04	N.D.	N.D.	N.D.	N.D.	N.D.	2.7E-04	6.8E-05	1.0E-03	1.8E-04	N.D.	N.A.	3.8	1.9E-04 ***	<1	N.A.	<1	N.A.
[illegible]	[illegible]	[illegible]	[illegible]	[illegible]	[illegible]	[illegible]	[illegible]	[illegible]	[illegible]	[illegible]	[illegible]	[illegible]	[illegible]	[illegible]	[illegible]	[illegible]	[illegible]	[illegible]	[illegible]	[illegible]	[illegible]	[illegible]	[illegible]	[illegible]	[illegible]	[illegible]	[illegible]	[illegible]	[illegible]	[illegible]	[illegible]	[illegible]	[illegible]

A_0099	UDP	UDP	C00015	HMDB00295	402.994	12.07	9.3E-04	1.4E-04	9.2E-04	2.2E-05	7.9E-05	1.2E-04	2.9E-05	N.D.	1.4E-04	1.2E-04	4.2E-04	4.1E-04	3.6E-04	8.7E-04	9.1E-04	4.2E-04	4.6E-04	1.0E-04	5.0E-05	5.9E-04	2.7E-04	0.2	0.205		1.4	0.489		5.8	0.014	*
A_0072	Glucose 6-phosphate	G6P	C00668,C0117	HMDB01401	259.021	10.12	3.3E-03	7.4E-03	5.9E-03	1.9E-03	6.5E-04	1.2E-03	1.1E-03	7.4E-04	9.3E-04	4.9E-04	4.6E-02	9.4E-02	6.3E-02	4.9E-02	5.6E-02	3.8E-03	2.8E-03	8.9E-04	2.8E-04	6.1E-02	1.9E-02	0.2	0.079		16	0.002	**	69	0.002	**
C_0142	Glutathione (GSH)	GSH	C00051	HMDB00125	308.090	13.60	6.3E-02	5.7E-02	4.9E-02	3.4E-02	2.3E-02	7.9E-03	1.3E-02	1.0E-02	9.4E-03	1.0E-02	1.6E-01	2.2E-01	1.5E-01	1.7E-01	1.6E-01	4.5E-02	1.6E-02	1.0E-02	1.8E-03	1.7E-01	2.7E-02	0.2	0.008	**	3.8	6.7E-05	***	17	1.8E-04	***
A_0098	CDP	CDP	C00112	HMDB01546	402.010	11.85	2.5E-04	3.8E-05	2.8E-04	1.6E-05	2.2E-05	2.9E-05	5.8E-06	N.D.	3.1E-05	3.2E-05	1.6E-04	1.0E-04	1.2E-04	3.3E-04	2.3E-04	1.2E-04	1.3E-04	2.5E-05	1.3E-05	1.9E-04	9.3E-05	0.2	0.177		1.5	0.392		7.6	0.017	*
A_0091	IMP	IMP	C00130	HMDB00175	347.039	9.80	2.7E-03	7.2E-04	4.0E-03	6.3E-04	1.3E-03	4.5E-04	N.D.	N.D.	4.1E-04	2.7E-04	1.0E-01	4.2E-02	4.0E-02	4.0E-02	5.0E-02	1.9E-03	1.5E-03	3.8E-04	9.3E-05	5.4E-02	2.6E-02	0.2	0.085		29	0.011	*	144	0.010	**
C_0032	Anserine_divalent	Anserine	C01262	HMDB00194	121.068	7.04	9.7E-03	1.1E-02	7.2E-02	1.8E-02	3.3E-02	3.2E-03	2.6E-03	3.5E-03	1.2E-03	2.0E-03	6.4E-02	1.1E-01	8.0E-02	9.7E-02	9.9E-02	2.9E-02	2.6E-02	2.5E-03	9.3E-04	9.0E-02	1.8E-02	0.09	0.089		3.1	0.003	**	36	3.7E-04	***
C_0120	Homocarnosine	Homocarnosine	C00884	HMDB00745	241.129	7.04	9.2E-04	1.2E-03	8.4E-03	1.9E-03	3.4E-03	2.9E-04	2.7E-04	3.9E-04	1.3E-04	2.3E-04	6.4E-03	1.2E-02	8.3E-03	1.1E-02	1.1E-02	3.1E-03	3.1E-03	2.6E-04	9.6E-05	9.7E-03	2.4E-03	0.08	0.103		3.1	0.006	**	37	8.8E-04	***
A_0019	6-Hydroxyhexanoic acid	6-Hydroxyhexanoic acid	C06103	No ID	131.069	8.56	N.D.	N.D.	N.D.	N.D.	N.D.	N.D.	N.D.	N.D.	N.D.	N.D.	N.D.	7.4E-05	N.D.	N.D.	N.D.	N.D.	N.A.	N.D.	N.A.	7.4E-05	N.A.	N.A.	N.A.		1<	N.A.		1<	N.A.	
A_0021	Threonic acid	Threonic acid	C01620	HMDB00943	135.031	9.20	N.D.	N.D.	N.D.	N.D.	N.D.	N.D.	N.D.	N.D.	N.D.	N.D.	3.2E-04	4.3E-04	4.2E-04	3.7E-04	3.1E-04	N.D.	N.A.	N.D.	N.A.	3.7E-04	5.4E-05	N.A.	N.A.		1<	N.A.		1<	N.A.	
A_0024	XA0004	XA0004	No ID	No ID	144.029	9.29	N.D.	N.D.	N.D.	N.D.	N.D.	N.D.	N.D.	N.D.	N.D.	N.D.	3.8E-05	N.D.	9.3E-05	5.4E-05	7.1E-05	N.D.	N.A.	N.D.	N.A.	6.4E-05	2.4E-05	N.A.	N.A.		1<	N.A.		1<	N.A.	
A_0027	2-Hydroxyglutaric acid	2-Hydroxyglutaric acid	C02630,C0108	HMDB00606,H	147.029	16.98	N.D.	N.D.	N.D.	N.D.	N.D.	N.D.	N.D.	N.D.	N.D.	N.D.	3.7E-04	3.0E-04	4.4E-04	6.0E-04	4.0E-04	N.D.	N.A.	N.D.	N.A.	4.2E-04	1.1E-04	N.A.	N.A.		1<	N.A.		1<	N.A.	
A_0030	8-Hydroxyoctanoic acid	8-Hydroxyoctanoic acid	No ID	No ID	159.101	8.04	N.D.	N.D.	N.D.	N.D.	N.D.	N.D.	N.D.	N.D.	N.D.	N.D.	N.D.	1.5E-04	N.D.	1.7E-04	1.4E-04	N.D.	N.A.	N.D.	N.A.	1.5E-04	1.6E-05	N.A.	N.A.		1<	N.A.		1<	N.A.	
A_0037	Glyceraldehyde 3-phosphate	GAP	C00118,C0066	HMDB01112	168.989	11.63	N.D.	N.D.	N.D.	N.D.	N.D.	N.D.	N.D.	N.D.	N.D.	N.D.	N.D.	N.D.	N.D.	5.9E-04	N.D.	N.D.	N.A.	N.D.	N.A.	5.9E-04	N.A.	N.A.	N.A.		1<	N.A.		1<	N.A.	
A_0045	Hippuric acid	Hippuric acid	C01586	HMDB00714	178.050	8.34	N.D.	N.D.	N.D.	N.D.	N.D.	N.D.	N.D.	N.D.	N.D.	N.D.	N.D.	2.1E-04	N.D.	1.1E-04	7.2E-05	N.D.	N.A.	N.D.	N.A.	1.3E-04	7.2E-05	N.A.	N.A.		1<	N.A.		1<	N.A.	
A_0049	*N*-Acetylglutamine	N-Acetylglutamine	No ID	HMDB06029	187.073	8.02	N.D.	N.D.	N.D.	N.D.	N.D.	N.D.	N.D.	N.D.	N.D.	N.D.	N.D.	N.D.	9.9E-05	N.D.	1.1E-04	N.D.	N.A.	N.D.	N.A.	1.1E-04	8.4E-06	N.A.	N.A.		1<	N.A.		1<	N.A.	
A_0051	10-Hydroxydecanoic acid	10-Hydroxydecanoic acid	C02774	No ID	187.133	7.71	N.D.	N.D.	N.D.	N.D.	N.D.	N.D.	N.D.	N.D.	N.D.	N.D.	1.9E-04	1.7E-04	1.5E-04	1.8E-04	1.9E-04	N.D.	N.A.	N.D.	N.A.	1.8E-04	1.6E-05	N.A.	N.A.		1<	N.A.		1<	N.A.	
A_0055	Phenaceturic acid	Phenaceturic acid	C05598	HMDB00821	192.066	8.13	N.D.	N.D.	N.D.	N.D.	N.D.	N.D.	N.D.	N.D.	N.D.	N.D.	7.7E-05	N.D.	3.3E-04	1.0E-04	N.D.	N.D.	N.A.	N.D.	N.A.	1.7E-04	1.4E-04	N.A.	N.A.		1<	N.A.		1<	N.A.	
A_0065	XA0027	XA0027	No ID	No ID	227.200	7.36	N.D.	N.D.	N.D.	N.D.	N.D.	N.D.	N.D.	N.D.	N.D.	N.D.	N.D.	3.6E-04	N.D.	5.0E-04	N.D.	N.D.	N.A.	N.D.	N.A.	4.3E-04	9.4E-05	N.A.	N.A.		1<	N.A.		1<	N.A.	
A_0069	Biotin	Biotin	C00120	HMDB00030	243.081	7.55	N.D.	N.D.	N.D.	N.D.	N.D.	N.D.	N.D.	N.D.	N.D.	N.D.	6.7E-04	6.8E-04	4.6E-04	5.7E-04	7.8E-04	N.D.	N.A.	N.D.	N.A.	6.3E-04	1.2E-04	N.A.	N.A.		1<	N.A.		1<	N.A.	
A_0070	Glucosamine 6-phosphate	Glc-6P	C00352	HMDB01254	258.038	8.85	N.D.	N.D.	N.D.	N.D.	N.D.	N.D.	N.D.	N.D.	N.D.	N.D.	4.3E-04	N.D.	6.8E-04	3.2E-04	2.8E-04	N.D.	N.A.	N.D.	N.A.	4.3E-04	1.8E-04	N.A.	N.A.		1<	N.A.		1<	N.A.	
A_0087	cAMP	cAMP	C00575	HMDB00058	328.045	7.43	N.D.	N.D.	N.D.	N.D.	N.D.	N.D.	N.D.	N.D.	N.D.	N.D.	2.4E-04	2.2E-04	2.7E-04	3.1E-04	3.0E-04	N.D.	N.A.	N.D.	N.A.	2.7E-04	4.0E-05	N.A.	N.A.		1<	N.A.		1<	N.A.	
A_0089	cGMP	cGMP	C00942	HMDB01314	344.039	7.51	N.D.	N.D.	N.D.	N.D.	N.D.	N.D.	N.D.	N.D.	N.D.	N.D.	1.1E-04	1.2E-04	1.5E-04	1.1E-04	N.D.	N.D.	N.A.	N.D.	N.A.	1.2E-04	1.7E-05	N.A.	N.A.		1<	N.A.		1<	N.A.	
A_0102	ThPP	ThPP	C00068	HMDB01372	423.029	7.88	N.D.	N.D.	N.D.	N.D.	N.D.	N.D.	N.D.	N.D.	N.D.	N.D.	6.5E-04	7.7E-04	7.5E-04	6.6E-04	6.7E-04	N.D.	N.A.	N.D.	N.A.	7.0E-04	5.5E-05	N.A.	N.A.		1<	N.A.		1<	N.A.	
A_0108	Octanoyl CoA_divalent	Octanoyl CoA	C01944	HMDB01070	445.602	9.53	N.D.	N.D.	N.D.	N.D.	N.D.	N.D.	N.D.	N.D.	N.D.	N.D.	6.3E-05	N.D.	6.9E-05	N.D.	5.7E-05	N.D.	N.A.	N.D.	N.A.	6.3E-05	6.2E-06	N.A.	N.A.		1<	N.A.		1<	N.A.	
A_0109	FMN	FMN	C00061	HMDB01520	455.099	8.26	N.D.	N.D.	N.D.	N.D.	N.D.	N.D.	N.D.	N.D.	N.D.	N.D.	7.9E-05	8.1E-05	1.2E-04	6.2E-05	5.0E-05	N.D.	N.A.	N.D.	N.A.	7.8E-05	2.7E-05	N.A.	N.A.		1<	N.A.		1<	N.A.	
A_0125	CMP-*N*-acetylneuraminate	CMP-NeuNAc	C00128	HMDB01176	613.140	8.09	N.D.	N.D.	N.D.	N.D.	N.D.	N.D.	N.D.	N.D.	N.D.	N.D.	4.7E-04	5.3E-04	4.9E-04	5.1E-04	4.7E-04	N.D.	N.A.	N.D.	N.A.	4.9E-04	2.9E-05	N.A.	N.A.		1<	N.A.		1<	N.A.	
A_0127	3'-Dephospho CoA	Dephospho-CoA	C00882	HMDB01373	686.140	7.93	N.D.	N.D.	N.D.	N.D.	N.D.	N.D.	N.D.	N.D.	N.D.	N.D.	N.D.	1.6E-04	N.D.	N.D.	N.D.	N.D.	N.A.	N.D.	N.A.	1.6E-04	N.A.	N.A.	N.A.		1<	N.A.		1<	N.A.	
C_0004	Methylguanidine	Methylguanidine	C02294	HMDB01522	74.071	6.36	N.D.	N.D.	N.D.	N.D.	N.D.	N.D.	N.D.	N.D.	N.D.	N.D.	N.D.	N.D.	N.D.	8.2E-04	8.7E-04	N.D.	N.A.	N.D.	N.A.	8.4E-04	3.9E-05	N.A.	N.A.		1<	N.A.		1<	N.A.	
C_0007	Morpholine	Morpholine	C14452	HMDB31581	88.076	6.82	N.D.	N.D.	N.D.	N.D.	N.D.	N.D.	N.D.	N.D.	N.D.	N.D.	8.4E-04	6.5E-04	1.0E-03	8.2E-04	6.1E-04	N.D.	N.A.	N.D.	N.A.	7.9E-04	1.6E-04	N.A.	N.A.		1<	N.A.		1<	N.A.	
C_0013	Homoserinelactone	Homoserinelactone	No ID	No ID	102.055	7.30	N.D.	N.D.	N.D.	N.D.	N.D.	N.D.	N.D.	N.D.	N.D.	N.D.	2.0E-04	2.1E-04	3.3E-04	N.D.	1.7E-04	N.D.	N.A.	N.D.	N.A.	2.3E-04	7.0E-05	N.A.	N.A.		1<	N.A.		1<	N.A.	
C_0045	3-Guanidinopropionic acid	3-Guanidinopropionic acid	C03065	No ID	132.076	8.25	N.D.	N.D.	N.D.	N.D.	N.D.	N.D.	N.D.	N.D.	N.D.	N.D.	4.2E-04	8.4E-04	3.2E-04	4.0E-04	6.2E-04	N.D.	N.A.	N.D.	N.A.	5.2E-04	2.1E-04	N.A.	N.A.		1<	N.A.		1<	N.A.	
C_0049	Gly-Gly	Gly-Gly	C02037	HMDB11733	133.060	8.63	N.D.	N.D.	N.D.	N.D.	N.D.	N.D.	N.D.	N.D.	N.D.	N.D.	5.3E-04	5.9E-04	6.9E-04	6.4E-04	5.4E-04	N.D.	N.A.	N.D.	N.A.	6.0E-04	6.9E-05	N.A.	N.A.		1<	N.A.		1<	N.A.	
C_0057	Trigonelline	Trigonelline	C01004	HMDB00875	138.056	10.78	N.D.	N.D.	N.D.	N.D.	N.D.	N.D.	N.D.	N.D.	N.D.	N.D.	2.6E-04	2.2E-04	N.D.	2.4E-04	1.7E-04	N.D.	N.A.	N.D.	N.A.	2.2E-04	3.9E-05	N.A.	N.A.		1<	N.A.		1<	N.A.	
C_0061	Ectoine	Ectoine	C06231	No ID	143.080	9.75	N.D.	N.D.	N.D.	N.D.	N.D.	N.D.	N.D.	N.D.	N.D.	N.D.	N.D.	N.D.	3.6E-04	N.D.	N.D.	N.D.	N.A.	N.D.	N.A.	3.6E-04	N.A.	N.A.	N.A.		1<	N.A.		1<	N.A.	
C_0064	Acetylcholine	Acetylcholine	C01996	HMDB00895	146.117	7.84	N.D.	N.D.	N.D.	N.D.	N.D.	N.D.	N.D.	N.D.	N.D.	N.D.	N.D.	N.D.	1.8E-04	N.D.	1.4E-04	N.D.	N.A.	N.D.	N.A.	1.6E-04	2.2E-05	N.A.	N.A.		1<	N.A.		1<	N.A.	
C_0069	*threo*-β-Methylaspartic acid	threo-b-Methylaspartic aci	C03618	No ID	148.060	12.73	N.D.	N.D.	N.D.	N.D.	N.D.	N.D.	N.D.	N.D.	N.D.	N.D.	2.3E-03	3.0E-03	1.1E-03	1.7E-03	1.7E-03	N.D.	N.A.	N.D.	N.A.	2.0E-03	7.1E-04	N.A.	N.A.		1<	N.A.		1<	N.A.	
C_0081	Methionine sulfoxide	Methionine sulfoxide	C02989	HMDB02005	166.052	12.28	N.D.	N.D.	N.D.	N.D.	N.D.	N.D.	N.D.	N.D.	N.D.	N.D.	N.D.	N.D.	3.8E-04	N.D.	3.8E-04	N.D.	N.A.	N.D.	N.A.	3.8E-04	1.6E-06	N.A.	N.A.		1<	N.A.		1<	N.A.	
C_0083	Taurocyamine	Taurocyamine	C01959	HMDB03584	168.042	22.99	N.D.	N.D.	N.D.	N.D.	N.D.	N.D.	N.D.	N.D.	N.D.	N.D.	8.8E-04	9.1E-04	1.5E-03	9.4E-04	1.1E-03	N.D.	N.A.	N.D.	N.A.	1.1E-03	2.6E-04	N.A.	N.A.		1<	N.A.		1<	N.A.	
C_0086	1-Methylhistidine	1-Methylhistidine	No ID	HMDB00001	170.092	7.72	N.D.	N.D.	N.D.	N.D.	N.D.	N.D.	N.D.	N.D.	N.D.	N.D.	9.0E-03	9.2E-03	9.9E-03	8.6E-03	9.6E-03	N.D.	N.A.	N.D.	N.A.	9.2E-03	5.0E-04	N.A.	N.A.		1<	N.A.		1<	N.A.	
C_0088	XC0040	XC0040	No ID	No ID	174.087	12.55	N.D.	N.D.	N.D.	N.D.	N.D.	N.D.	N.D.	N.D.	N.D.	N.D.	4.2E-04	3.4E-04	4.4E-04	5.1E-04	4.0E-04	N.D.	N.A.	N.D.	N.A.	4.2E-04	6.3E-05	N.A.	N.A.		1<	N.A.		1<	N.A.	
C_0098	N^6-Acetyllysine	N6-Acetyllysine	C02727	HMDB00206	189.123	11.81	N.D.	N.D.	N.D.	N.D.	N.D.	N.D.	N.D.	N.D.	N.D.	N.D.	N.D.	N.D.	N.D.	2.7E-04	N.D.	N.D.	N.A.	N.D.	N.A.	2.7E-04	N.A.	N.A.	N.A.		1<	N.A.		1<	N.A.	
C_0100	*N*-Acetyllysine	N-Acetyllysine	C12989	HMDB00446	189.123	10.18	N.D.	N.D.	N.D.	N.D.	N.D.	N.D.	N.D.	N.D.	N.D.	N.D.	1.1E-03	8.2E-04	9.9E-04	8.9E-04	7.8E-04	N.D.	N.A.	N.D.	N.A.	9.2E-04	1.3E-04	N.A.	N.A.		1<	N.A.		1<	N.A.	
C_0102	Homocitrulline	Homocitrulline	C02427	HMDB00679	190.118	11.53	N.D.	N.D.	N.D.	N.D.	N.D.	N.D.	N.D.	N.D.	N.D.	N.D.	3.5E-04	2.1E-04	2.2E-04	2.0E-04	2.5E-04	N.D.	N.A.	N.D.	N.A.	2.5E-04	6.2E-05	N.A.	N.A.		1<	N.A.		1<	N.A.	
C_0109	Carboxymethyllysine	Carboxymethyllysine	No ID	No ID	205.119	9.50	N.D.	N.D.	N.D.	N.D.	N.D.	N.D.	N.D.	N.D.	N.D.	N.D.	6.5E-04	5.5E-04	5.8E-04	5.3E-04	8.0E-04	N.D.	N.A.	N.D.	N.A.	6.2E-04	1.1E-04	N.A.	N.A.		1<	N.A.		1<	N.A.	
C_0116	Ergothioneine	Ergothioneine	C05570	HMDB03045	230.095	18.08	N.D.	N.D.	N.D.	N.D.	N.D.	N.D.	N.D.	N.D.	N.D.	N.D.	N.D.	7.7E-04	N.D.	N.D.	N.D.	N.D.	N.A.	N.D.	N.A.	7.7E-04	N.A.	N.A.	N.A.		1<	N.A.		1<	N.A.	
C_0118	XC0071	XC0071	No ID	No ID	234.180	6.40	N.D.	N.D.	N.D.	N.D.	N.D.	N.D.	N.D.	N.D.	N.D.	N.D.	2.4E-04	2.7E-04	3.0E-04	3.1E-04	3.4E-04	N.D.	N.A.	N.D.	N.A.	2.9E-04	3.7E-05	N.A.	N.A.		1<	N.A.		1<	N.A.	
C_0125	Malonylcarnitine	Malonylcarnitine	No ID	HMDB02095	248.111	10.54	N.D.	N.D.	N.D.	N.D.	N.D.	N.D.	N.D.	N.D.	N.D.	N.D.	1.5E-04	1.8E-04	2.5E-04	3.4E-04	3.0E-04	N.D.	N.A.	N.D.	N.A.	2.4E-04	8.0E-05	N.A.	N.A.		1<	N.A.		1<	N.A.	
C_0126	Pyridoxamine-P	Pyridoxamine-P	C00647	HMDB01555	249.063	10.80	N.D.	N.D.	N.D.	N.D.	N.D.	N.D.	N.D.	N.D.	N.D.	N.D.	1.6E-03	1.7E-03	1.6E-03	1.7E-03	1.5E-03	N.D.	N.A.	N.D.	N.A.	1.6E-03	9.6E-05	N.A.	N.A.		1<	N.A.		1<	N.A.	
C_0127	γ-Glu-Cys	g-Glu-Cys	C00669	HMDB01049	251.069	13.23	N.D.	N.D.	N.D.	N.D.	N.D.	N.D.	N.D.	N.D.	N.D.	N.D.	1.6E-04	3.0E-04	3.3E-04	2.0E-04	1.9E-04	N.D.	N.A.	N.D.	N.A.	2.4E-04	7.6E-05	N.A.	N.A.		1<	N.A.		1<	N.A.	
C_0131	Thiamine	Thiamine	C00378	HMDB00235	265.110	6.78	N.D.	N.D.	N.D.	N.D.	N.D.	N.D.	N.D.	N.D.	N.D.	N.D.	1.3E-04	1.2E-04	1.0E-04	8.5E-05	9.9E-05	N.D.	N.A.	N.D.	N.A.	1.1E-04	2.0E-05	N.A.	N.A.		1<	N.A.		1<	N.A.	
C_0135	Saccharopine	Saccharopine	C00449	HMDB00279	277.139	11.05	N.D.	N.D.	N.D.	N.D.	N.D.	N.D.	N.D.	N.D.	N.D.	N.D.	5.0E-04	3.4E-04	2.4E-04	5.0E-04	3.7E-04	N.D.	N.A.	N.D.	N.A.	3.9E-04	1.1E-04	N.A.	N.A.		1<	N.A.		1<	N.A.	
C_0137	Octanoylcarnitine	Octanoylcarnitine	C02838	HMDB00791	288.215	10.73	N.D.	N.D.	N.D.	N.D.	N.D.	N.D.	N.D.	N.D.	N.D.	N.D.	2.9E-04	1.5E-04	3.9E-04	6.6E-04	4.1E-04	N.D.	N.A.	N.D.	N.A.	3.8E-04	1.9E-04	N.A.	N.A.		1<	N.A.		1<	N.A.	
C_0139	Argininosuccinic acid	ArgSuccinate	C03406	HMDB00052	291.130	9.72	N.D.	N.D.	N.D.	N.D.	N.D.	N.D.	N.D.	N.D.	N.D.	N.D.	7.3E-04	6.0E-04	1.1E-03	6.8E-04	1.1E-03	N.D.	N.A.	N.D.	N.A.	8.5E-04	2.4E-04	N.A.	N.A.		1<	N.A.		1<	N.A.	
C_0140	5'-Deoxy-5'-methylthioadenosine	MTA	C00170	HMDB01173	298.097	10.41	N.D.	N.D.	N.D.	N.D.	N.D.	N.D.	N.D.	N.D.	N.D.	N.D.	7.8E-05	1.7E-04	8.4E-05	1.0E-04	9.5E-05	N.D.	N.A.	N.D.	N.A.	1.1E-04	3.8E-05	N.A.	N.A.		1<	N.A.		1<	N.A.	
C_0146	*S*-Lactoylglutathione	S-Lactoylglutathione	C03451	HMDB01066	380.112	14.24	N.D.	N.D.	N.D.	N.D.	N.D.	N.D.	N.D.	N.D.	N.D.	N.D.	1.0E-03	3.5E-03	9.8E-04	1.4E-03	3.4E-03	N.D.	N.A.	N.D.	N.A.	2.1E-03	1.3E-03	N.A.	N.A.		1<	N.A.		1<	N.A.	

C and A indicate cation and anion mode, respectively.

N.D.: Not Detected

N.A.: Not Available

[||] Welch's t-test, p-value.*<0.05, **<0.01, ***<0.001
