# Supplementary material for: Altered choline level in atherosclerotic lesions: Upregulation of choline transporter-like protein 1 in human coronary unstable plaque
Source: PLoS One. 2023 Feb 17;18(2):e0281730. doi: 10.1371/journal.pone.0281730 (PMC9937458; doi:10.1371/journal.pone.0281730)
Supplement: S9 Table — (PDF) [file pone.0281730.s009.PDF]

Supplementary table 9. Arterial and cardiac metabolite levels in rabbits fed a conventional diet

ID	Metabolite	KEGG ID	HMDB ID	Concentration (nmol/g)																				
				non-injured artery					injured artery					heart					non-injured artery		injured artery		heart	
				1	2	3	4	5	6	7	8	9	10	11	12	13	14	15	Mean	S.D.	Mean	S.D.	Mean	S.D.
A_0001	Glyoxylic acid	C00048	HMDB00119	N.D.	N.D.	N.D.	N.D.	N.D.	N.D.	N.D.	8.7	N.D.	N.D.	N.D.	N.D.	N.D.	N.D.	N.D.	N.D.	N.A.	8.7	N.A.	N.D.	N.A.
A_0002	Glycolic acid	C00160	HMDB00115	N.D.	N.D.	N.D.	N.D.	N.D.	N.D.	N.D.	N.D.	N.D.	N.D.	N.D.	N.D.	N.D.	N.D.	N.D.	N.D.	N.A.	N.D.	N.A.	N.D.	N.A.
A_0003	Pyruvic acid	C00022	HMDB00243	N.D.	N.D.	N.D.	N.D.	15	N.D.	N.D.	31	N.D.	N.D.	N.D.	N.D.	N.D.	N.D.	N.D.	15	N.A.	31	N.A.	N.D.	N.A.
A_0004	Lactic acid	C00186,C0025	HMDB00190,H	596	850	1,370	669	1,759	2,252	1,413	1,879	1,456	1,320	14,847	13,244	19,595	16,778	11,487	1,049	499	1,664	392	15,190	3,144
A_0005	3-Hydroxybutyric acid	C01089,C0319	HMDB00011,H	28	12	14	9.2	31	69	18	10	20	26	135	394	371	201	148	19	9.9	29	23	250	124
A_0006	2-Hydroxybutyric acid	C05984	HMDB00008	N.D.	1.1	N.D.	N.D.	N.D.	2.9	1.3	N.D.	1.2	1.6	N.D.	12	11	N.D.	N.D.	1.1	N.A.	1.7	0.8	11	0.3
A_0008	Fumaric acid	C00122	HMDB00134	N.D.	9.1	7.3	3.4	9.6	19	8.6	7.7	8.6	5.7	289	305	325	354	401	7.4	2.8	9.9	5.2	334	44
A_0009	2-Oxoisovaleric acid	C00141	HMDB00019	N.D.	N.D.	N.D.	N.D.	N.D.	N.D.	N.D.	N.D.	N.D.	N.D.	N.D.	N.D.	N.D.	N.D.	N.D.	N.D.	N.A.	N.D.	N.A.	N.D.	N.A.
A_0012	Succinic acid	C00042	HMDB00254	10	19	18	12	13	28	18	26	19	12	830	1,044	1,364	532	316	15	4.0	21	6.4	817	413
A_0022	Malic acid	C00149,C0049	HMDB00156,H	23	42	40	21	64	95	41	46	44	37	886	944	915	1,110	1,144	38	17	52	24	1,000	118
A_0026	2-Oxoglutaric acid	C00026	HMDB00208	N.D.	N.D.	N.D.	N.D.	N.D.	N.D.	N.D.	N.D.	N.D.	N.D.	N.D.	N.D.	N.D.	N.D.	N.D.	N.D.	N.A.	N.D.	N.A.	N.D.	N.A.
A_0035	Phosphoenolpyruvic acid	C00074	HMDB00263	1.5	16	12	5.2	11	16	13	6.5	11	8.7	N.D.	N.D.	N.D.	N.D.	N.D.	9.1	5.6	11	3.8	N.D.	N.A.
A_0037	Dihydroxyacetone phosphate	C00111	HMDB01473	5.4	14	23	22	25	31	48	21	36	19	152	125	118	178	375	18	8.1	31	12	190	107
A_0038	Glyceraldehyde 3-phosphate	C00118,C0066	HMDB01112	N.D.	N.D.	N.D.	N.D.	N.D.	N.D.	N.D.	N.D.	N.D.	N.D.	N.D.	N.D.	N.D.	N.D.	N.D.	N.D.	N.A.	N.D.	N.A.	N.D.	N.A.
A_0039	Glycerol 3-phosphate	C00093	HMDB00126	65	131	125	33	116	182	51	83	81	68	1,338	1,674	1,569	1,503	1,802	94	43	93	51	1,577	175
A_0042	*cis*-Aconitic acid	C00417	HMDB00072	52	N.D.	16	18	15	1.6	N.D.	24	14	17	15	15	18	12	15	25	18	14	9.5	15	2.0
A_0046	2-Phosphoglyceric acid	C00631	HMDB03391	2.7	8.6	9.0	5.9	12	11	7.6	7.6	9.3	7.3	N.D.	N.D.	N.D.	N.D.	N.D.	7.7	3.6	8.5	1.5	N.D.	N.A.
A_0047	3-Phosphoglyceric acid	C00197	HMDB00807	15	57	60	35	86	73	53	46	61	42	23	23	20	N.D.	50	51	27	55	12	29	14
A_0051	Isocitric acid	C00311	HMDB00193	22	N.D.	15	21	11	N.D.	N.D.	15	16	14	12	17	12	13	15	17	5.3	15	1.0	14	2.0
A_0053	Citric acid	C00158	HMDB00094	4,010	31	653	825	765	57	34	1,107	441	759	588	620	614	467	742	1,257	1,571	480	461	606	98
A_0055	Gluconic acid	C00257	HMDB00625	20	15	5.3	8.9	8.9	7.8	4.5	11	3.6	4.9	N.D.	N.D.	N.D.	N.D.	N.D.	12	5.7	6.4	3.2	N.D.	N.A.
A_0056	Erythrose 4-phosphate	C00279,C0360	HMDB01321	15	N.D.	69	N.D.	N.D.	67	103	N.D.	N.D.	N.D.	N.D.	N.D.	N.D.	N.D.	N.D.	42	38	85	25	N.D.	N.A.
A_0065	Ribulose 5-phosphate	C00199,C0110	HMDB00618	4.9	78	56	25	66	136	132	63	94	39	133	123	145	125	148	46	30	93	42	135	11
A_0066	Ribose 5-phosphate	C00117	HMDB01548	N.D.	27	20	11	17	54	52	22	41	13	N.D.	22	13	13	31	19	6.7	36	18	20	8.6
A_0070	Glucose 1-phosphate	C00103	HMDB01586	45	4.5	69	14	32	11	19	43	18	36	155	33	80	201	378	33	26	25	13	169	133
A_0071	Glucose 6-phosphate	C00668,C0117	HMDB01401	N.D.	8.8	162	29	66	14	56	52	35	31	1,028	354	625	1,503	2,342	67	68	38	17	1,171	786
A_0073	Fructose 6-phosphate	C05345,C0008	HMDB00124	N.D.	3.3	23	8.0	N.D.	4.8	13	N.D.	9.6	N.D.	257	87	172	409	480	11	10	9.0	3.9	281	163
A_0076	6-Phosphogluconic acid	C00345	HMDB01316	4.8	1.8	25	3.9	16	8.5	19	29	9.2	14	11	8.1	N.D.	N.D.	17	10	10	16	8.3	12	4.7
A_0078	Sedoheptulose 7-phosphate	C05382	HMDB01068	N.D.	13	3.2	1.6	2.2	22	24	4.5	8.7	2.4	3.5	4.0	3.2	N.D.	2.5	4.9	5.3	12	9.9	3.3	0.6
A_0083	dTMP	C00364	HMDB01227	N.D.	N.D.	N.D.	N.D.	N.D.	N.D.	N.D.	N.D.	N.D.	N.D.	N.D.	N.D.	N.D.	N.D.	N.D.	N.D.	N.A.	N.D.	N.A.	N.D.	N.A.
A_0084	CMP	C00055	HMDB00095	4.5	2.8	12	1.3	5.1	1.0	1.4	2.3	1.6	2.1	33	12	17	24	21	5.1	4.1	1.7	0.5	21	7.8
A_0085	UMP	C00105	HMDB00288	10	2.7	13	1.6	4.0	1.0	1.0	2.1	1.8	2.3	73	31	44	64	41	6.3	4.9	1.6	0.6	51	18
A_0086	cAMP	C00575	HMDB00058	N.D.	N.D.	N.D.	N.D.	N.D.	N.D.	N.D.	N.D.	N.D.	N.D.	5.0	3.8	3.8	3.3	3.8	N.D.	N.A.	N.D.	N.A.	3.9	0.7
A_0087	Fructose 1,6-diphosphate	C00354	HMDB01058	33	3.2	37	14	53	10	8.7	37	22	20	1,213	214	571	1,022	1,384	28	20	20	11	881	481
A_0088	cGMP	C00942	HMDB01314	N.D.	N.D.	N.D.	N.D.	N.D.	N.D.	N.D.	N.D.	N.D.	N.D.	1.5	N.D.	2.2	N.D.	2.0	N.D.	N.A.	N.D.	N.A.	1.9	0.4
A_0089	AMP	C00020	HMDB00045	138	62	58	20	39	26	31	16	14	28	2,802	2,590	3,337	3,201	2,122	63	45	23	7.6	2,811	488
A_0090	IMP	C00130	HMDB00175	66	2.7	129	2.4	22	N.D.	N.D.	N.D.	N.D.	4.4	1,203	320	160	894	3,371	44	54	4.4	N.A.	1,189	1,290
A_0091	GMP	C00144	HMDB01397	21	6.0	31	2.4	14	1.7	1.5	3.3	3.8	5.5	135	114	145	147	158	15	12	3.2	1.7	140	17
A_0092	CoA_divalent	C00010	HMDB01423	1.1	N.D.	2.5	0.5	0.9	0.2	0.12	1.9	0.2	0.5	42	17	35	27	26	1.2	0.9	0.6	0.7	30	9.5
A_0093	PRPP	C00119	HMDB00280	N.D.	N.D.	N.D.	N.D.	N.D.	N.D.	N.D.	N.D.	N.D.	N.D.	2.5	N.D.	N.D.	N.D.	N.D.	N.D.	N.A.	N.D.	N.A.	2.5	N.A.
A_0095	dTDP	C00363	HMDB01274	N.D.	N.D.	N.D.	N.D.	N.D.	N.D.	N.D.	N.D.	N.D.	N.D.	N.D.	N.D.	N.D.	N.D.	N.D.	N.D.	N.A.	N.D.	N.A.	N.D.	N.A.
A_0096	CDP	C00112	HMDB01546	6.7	0.2	1.4	0.4	1.0	N.D.	0.11	0.4	N.D.	N.D.	4.5	N.D.	1.7	1.6	3.6	1.9	2.7	0.2	0.2	2.9	1.4
A_0097	UDP	C00015	HMDB00295	37	0.7	5.4	1.3	3.6	0.7	0.8	1.8	0.8	2.1	13	3.4	5.7	6.8	8.5	9.6	15	1.2	0.7	7.4	3.4
A_0098	Acetyl CoA_divalent	C00024	HMDB01206	0.2	N.D.	0.7	0.7	2.2	N.D.	N.D.	0.9	0.4	1.7	0.9	11	0.9	1.1	13	0.9	0.9	1.0	0.7	5.3	6.0
A_0101	Malonyl CoA_divalent	C00083	HMDB01175	N.D.	N.D.	N.D.	N.D.	N.D.	N.D.	N.D.	N.D.	N.D.	N.D.	N.D.	N.D.	N.D.	N.D.	N.D.	N.D.	N.A.	N.D.	N.A.	N.D.	N.A.
A_0103	ADP	C00008	HMDB01341	171	62	168	76	127	39	38	80	52	75	1,341	546	882	794	811	121	51	57	20	875	290
A_0104	GDP	C00035	HMDB01201	50	2.0	12	5.2	11	1.4	1.3	8.1	3.1	7.0	46	46	54	41	44	16	20	4.2	3.2	46	4.9
A_0109	dCTP	C00458	HMDB00998	N.D.	N.D.	N.D.	N.D.	N.D.	N.D.	N.D.	N.D.	N.D.	N.D.	N.D.	N.D.	N.D.	N.D.	N.D.	N.D.	N.A.	N.D.	N.A.	N.D.	N.A.
A_0110	dTTP	C00459	HMDB01342	N.D.	N.D.	N.D.	N.D.	N.D.	N.D.	N.D.	N.D.	N.D.	N.D.	N.D.	N.D.	N.D.	N.D.	N.D.	N.D.	N.A.	N.D.	N.A.	N.D.	N.A.
A_0111	CTP	C00063	HMDB00082	1.0	N.D.	N.D.	N.D.	N.D.	N.D.	N.D.	N.D.	N.D.	N.D.	N.D.	N.D.	N.D.	N.D.	N.D.	1.0	N.A.	N.D.	N.A.	N.D.	N.A.
A_0112	UTP	C00075	HMDB00285	1.9	N.D.	0.3	N.D.	0.3	0.2	N.D.	0.3	0.2	N.D.	2.8	0.8	N.D.	N.D.	2.0	0.9	0.9	0.2	0.09	1.9	1.0
A_0114	dATP	C00131	HMDB01532	N.D.	N.D.	N.D.	N.D.	N.D.	N.D.	N.D.	N.D.	N.D.	N.D.	N.D.	N.D.	N.D.	N.D.	N.D.	N.D.	N.A.	N.D.	N.A.	N.D.	N.A.
A_0115	ATP	C00002	HMDB00538	7.7	14	23	5.6	31	29	13	26	13	6.1	360	57	105	81	279	17	11	17	9.6	177	135
A_0116	GTP	C00044	HMDB01273	5.2	0.3	1.5	0.5	1.5	0.8	N.D.	2.2	0.4	0.4	20	11	11	8.9	25	1.8	2.0	1.0	0.8	15	7.1
A_0124	NAD^+	C00003	HMDB00902	78	60	72	7.3	46	31	18	71	6.8	35	44	9.3	84	2.5	59	53	28	32	24	40	34
A_0125	$NADP^+$	C00006	HMDB00217	8.7	2.7	7.2	1.4	5.2	3.0	1.7	8.1	0.9	3.9	N.D.	N.D.	N.D.	N.D.	N.D.	5.1	3.0	3.5	2.8	N.D.	N.A.
C_0004	Gly	C00037	HMDB00123	685	554	650	124	577	1,146	647	619	406	417	439	406	488	511	482	518	227	647	300	465	42
C_0007	Putrescine	C00134	HMDB01414	3.2	6.8	6.0	0.7	5.3	6.8	7.5	5.6	3.5	4.1	12	8.0	8.8	9.6	14	4.4	2.5	5.5	1.7	10	2.5
C_0008	Sarcosine	C00213	HMDB00271	2.5	1.1	3.3	N.D.	2.7	4.2	2.5	2.7	1.1	1.3	N.D.	N.D.	N.D.	N.D.	N.D.	2.4	1.0	2.3	1.2	N.D.	N.A.
C_0009	Ala	C00041,C0013	HMDB00161,H	190	272	266	55	149	347	287	252	168	112	2,157	3,136	3,741	3,395	1,796	186	90	233	93	2,845	831
C_0010	β-Ala	C00099	HMDB00056	18	27	30	4.2	26	46	39	38	17	22	8.8	6.6	10	10	8.9	21	10	33	12	8.9	1.4
C_0013	*N,N*-Dimethylglycine	C01026	HMDB00092	N.D.	N.D.	N.D.	N.D.	N.D.	N.D.	N.D.	N.D.	N.D.	N.D.	1.7	N.D.	1.4	N.D.	1.2	N.D.	N.A.	N.D.	N.A.	1.4	0.3
C_0015	GABA	C00334	HMDB00112	3.0	4.2	5.1	0.8	4.1	8.9	8.1	7.5	3.9	3.8	N.D.	1.7	N.D.	N.D.	N.D.	3.4	1.6	6.4	2.4	1.7	N.A.
C_0016	Choline	C00114	HMDB00097	115	205	191	69	148	252	211	154	165	113	134	145	68	90	102	146	56	179	54	108	32
C_0017	Ser	C00065,C0071	HMDB00187,H	94	113	117	32	91	192	135	117	101	69	339	280	353	325	194	89	34	123	46	298	64
C_0020	Cytosine	C00380	HMDB00630	N.D.	N.D.	N.D.	N.D.	N.D.	N.D.	N.D.	N.D.	N.D.	N.D.	N.D.	N.D.	N.D.	N.D.	N.D.	N.D.	N.A.	N.D.	N.A.	N.D.	N.A.
C_0022	Uracil	C00106	HMDB00300	N.D.	12	7.9	3.6	10	34	32	19	19	9.8	16	28	23	14	22	8.4	3.6	23	10	21	5.7
C_0023	Creatinine	C00791	HMDB00562	14	21	18	7.1	25	31	19	17	18	25	95	104	97	79	126	17	6.9	22	5.8	100	17
C_0024	Pro	C00148,C0076	HMDB00162,H	23	32	47	11	35	71	60	63	48	40	158	142	180	121	88	30	14	56	12	138	35
C_0026	Val	C00183,C0641	HMDB00883	37	49	63	18	42	79	72	76	67	43	87	134	167	110	84	42	17	67	14	116	35
C_0027	Betaine	C00719	HMDB00043	26	24	28	9.7	37	42	29	26	25	32	508	675	658	576	552	25	9.7	31	6.8	594	71
C_0028	Thr	C00188,C0082	HMDB00167	57	105	94	22	75	108	120	102	78	62	141	226	199	192	187	71	33	94	24	189	31
C_0029	Homoserine	C00263	HMDB00719	N.D.	N.D.	N.D.	N.D.	N.D.	0.5	N.D.	0.4	N.D.	N.D.	0.9	N.D.	1.5	1.3	1.0	N.D.	N.A.	0.4	0.12	1.2	0.3
C_0030	Betaine aldehyde_+H_2O	C00576	HMDB01252	N.D.	N.D.	N.D.	N.D.	N.D.	N.D.	N.D.	N.D.	0.6	N.D.	N.D.	N.D.	N.D.	N.D.	N.D.	N.D.	N.A.	0.6	N.A.	N.D.	N.A.
C_0032	Cys	C00097,C0073	HMDB00574,H	N.D.	N.D.	N.D.	1.8	4.4	N.D.	N.D.	4.3	2.0	2.5	N.D.	N.D.	N.D.	N.D.	N.D.	3.1	1.8	2.9	1.2	N.D.	N.A.
C_0038	Thymine	C00178	HMDB00262	N.D.	N.D.	N.D.	N.D.	N.D.	N.D.	N.D.	N.D.	N.D.	N.D.	N.D.	N.D.	N.D.	N.D.	N.D.	N.D.	N.A.	N.D.	N.A.	N.D.	N.A.
C_0043	Hydroxyproline	C01157	HMDB00725	3.3	6.3	6.2	1.3	5.8	11	8.6	6.8	6.1	5.6	19	14	14	13	16	4.6	2.2	7.6	2.2	15	2.5
C_0045	Creatine	C00300	HMDB00064	736	1,043	1,169	220	900	1,094	914	648	459	602	489	418	467	431	452	814	369	744	256	451	28
C_0046	Ile	C00407,C0641	HMDB00172	10	29	32	9.0	23	42	39	35	35	20	26	63	80	48	28	20	11	34	8.7	49	23
C_0047	Leu	C00123,C0157	HMDB00687	22	38	45	15	31	56	49	52	53	31	52	132	160	101	56	30	12	48	10	100	47
C_0050	Asn	C00152,C0190	HMDB00168	20	36	33	9.6	27	49	39	31	33	21	228	251	212	252	209	25	11	35	10	230	21
C_0051	Ornithine	C00077,C0051	HMDB00214,H	3.2	7.1	6.6	2.3	4.7	7.8	8.8	7.0	6.3	5.4	28	36	29	29	27	4.8	2.1	7.1	1.3	30	3.4
C_0053	Asp	C00049,C0040	HMDB00191,H	187	210	246	45	209	353	230	329	183	95	553	389	315	482	457	180	78	238	106	439	91
C_0054	Adenine	C00147	HMDB00034	0.5	1.5	2.0	0.4	1.6	1.9	2.6	2.9	1.0	1.6	4.1	24	12	7.8	2.4	1.2	0.7	2.0	0.8	10	8.5
C_0055	Hypoxanthine	C00262	HMDB00157	8.0	60	35	14	39	149	142	33	68	20	73	130	105	74	72	31	21	82	60	91	26
C_0056	Anthranilic acid	C00108	HMDB01123	N.D.	N.D.	N.D.	N.D.	N.D.	N.D.	N.D.	N.D.	N.D.	N.D.	N.D.	N.D.	N.D.	N.D.	N.D.	N.D.	N.A.	N.D.	N.A.	N.D.	N.A.
C_0058	Tyramine	C00483	HMDB00306	0.2	0.3	N.D.	N.D.	0.3	N.D.	N.D.	0.2	0.4	0.2	N.D.	N.D.	N.D.	N.D.	N.D.	0.3	0.05	0.3	0.10	N.D.	N.A.
C_0066	Spermidine	C00315	HMDB01257	54	28	60	18	49	35	42	28	28	39	79	114	95	90	120	42	18	34	6.5	100	17
C_0067	Gln	C00064,C0030	HMDB00641,H	477	480	659	105	427	797	547	522	316	249	8,513	8,200	7,796	7,136	8,368	430	202	486	216	8,002	554
C_0068	Lys	C00047,C0073	HMDB00182,H	42	58	77	17	51	91	76	95	63	56	67	147	165	117	80	49	22	76	17	115	42
C_0070	Glu	C00025,C0021	HMDB00148,H	2,016	2,401	2,314	379	1,685	3,366	2,905	2,261	1,538	1,158	4,010	3,150	2,251	2,841	4,852	1,759	821	2,246	918	3,421	1,021
C_0072	Met	C00073,C0085	HMDB00696	5.7	19	15	8.3	16	27	25	22	26	16	27	35	41	30	27	13	5.5	23	4.7	32	6.0
C_0074	Guanine	C00242	HMDB00132	0.6	7.0	2.8	2.0	4.6	16	16	5.9	9.8	3.7	N.D.	3.1	2.0	1.5	1.3	3.4	2.5	10	5.6	2.0	0.8
C_0075	His	C00135,C0076	HMDB00177	19	31	29	9.8	27	45	37	32	33	26	440	518	358	488	388	23	8.9	35	7.1	438	67
C_0081	Phe	C00079,C0205	HMDB00159	13	21	22	7.4	18	29	29	27	24	18	56	76	83	64	62	16	6.1	25	4.6	68	11
C_0090	Arg	C00062,C0079	HMDB00517,H	42	75	76	16	55	100	89	86	66	56	260	325	261	244	282	53	25	79	18	274	31
C_0091	Citrulline	C00327	HMDB00904	6.8	9.1	14	4.1	12	13	10	15	13	11	26	20	34	26	29	9.2	4.0	12	1.7	27	5.0
C_0094	Tyr	C00082,C0153	HMDB00158	12	23	22	8.7	17	35	31	29	29	18	48	55	72	48	40	16	6.1	28	6.4	53	12
C_0105	Spermine	C00750	HMDB01256	76	0.7	20	6.4	18	9.8	14	3.4	2.6	9.1	29	28	28	20	29	24	30	7.8	4.7	27	4.0
C_0107	Trp	C00078,C0052	HMDB00929	2.7	6.6	3.5	2.2	5.5	10	8.2	8.7	7.3	6.5	15	22	25	15	16	4.1	1.9	8.2	1.5	18	4.5
C_0113	Carnosine	C00386	HMDB00033	11	3.5	4.9	4.5	10	23	13	19	8.5	12	389	450	603	508	528	6.8	3.5	15	5.8	496	81
C_0119	Thymidine	C00214	HMDB00273	N.D.	N.D.	N.D.	N.D.	N.D.	N.D.	N.D.	N.D.	N.D.	N.D.	N.D.	N.D.	N.D.	N.D.	N.D.	N.D.	N.A.	N.D.	N.A.	N.D.	N.A.
C_0120	Cytidine	C00475	HMDB00089	2.3	20	14	5.2	14	34	28	23	18	14	28	38	31	28	46	11	7.2	24	8.2	34	7.7
C_0121	Uridine	C00299	HMDB00296	17	102	78	29	62	207	170	151	135	84	50	62	55	66	90	57	35	149	45	65	16
C_0130	Adenosine	C00212	HMDB00050	10	32	11	5.9	27	45	45	141	20	97	10	281	24	12	7.4	17	11	69	49	67	120
C_0131	Inosine	C00294	HMDB00195	329	973	869	238	701	1,133	1,029	847	567	495	322	1,325	660	521	358	622	326	814	279	637	407
C_0134	Guanosine	C00387	HMDB00133	9.7	118	78	26	77	118	103	121	71	66	2.4	20	9.1	5.1	3.4	62	44	96	26	8.0	7.2
C_0139	Glutathione (GSSG)_divalent	C00127	HMDB03337	23	369	45	18	95	250	215	177	146	92	578	378	513	391	345	110	148	176	61	441	100
C_0140	Glutathione (GSH)	C00051	HMDB00125	188	75	526	110	398	135	165	335	105	139	722	1,101	1,102	1,348	1,326	259	195	176	91	1,120	252
C_0146	*S*-Adenosylmethionine	C00019	HMDB01185	4.0	6.5	7.5	1.6	6.3	6.8	6.6	6.4	4.2	4.8	20	20	21	21	18	5.2	2.4	5.8	1.2	20	1.1

C and A indicate cation and anion mode, respectively.
N.D.: Not Detected
N.A.: Not Available
