# Supplementary material for: Altered choline level in atherosclerotic lesions: Upregulation of choline transporter-like protein 1 in human coronary unstable plaque
Source: PLoS One. 2023 Feb 17;18(2):e0281730. doi: 10.1371/journal.pone.0281730 (PMC9937458; doi:10.1371/journal.pone.0281730)
Supplement: S10 Table — (PDF) [file pone.0281730.s010.PDF]

Supplementary table 10. Clinical background of autopsy cases

	control (n=6)	OMI (n=6)	AMI (n=5)
Average age (years)	66.8 (62-75)	72.5 (55-83)	76.6 (71-85)
Male sex	3 (50 %)	5 (100%)	3 (60 %)
Obesity	2 (33 %)	2 (33%)	1 (20 %)
Smoking	2 (33 %)	3 (50%)	3 (60 %)
Diabates	1 (16 %)	1 (16%)	0 (0 %)
Dyslipidemia	1 (16 %)	2 (33%)	2 (40 %)
Hypertension	2 (33 %)	4 (66 %)	3 (75 %)
Medication			
Antidiabetic	1 (16 %)	1 (16%)	0 (0 %)
Antihypertensive	2 (33 %)	4 (66 %)	2 (40 %)
Anticoagulant	1 (16 %)	2 (33%)	0 (0%)
Antiplatelet	0 (0 %)	3 (50%)	0 (0 %)
Statins	1 (16 %)	2 (33%)	0 (0 %)
